# Screening of Xeroprotectants for Efficient Mammalian Cell Structural Preservation in Dry Conditions

**DOI:** 10.1021/acsomega.6c04138

**Published:** 2026-08-17

**Authors:** Valentin Diez-Cabanes, Takahiro Kikawada, Pasqualino Loi, Santiago Aparicio

**Affiliations:** † International Research Center in Critical Raw Materials for Advanced Industrial Technologies (ICCRAM), 16725 University of Burgos, Burgos 09001, Spain; ‡ Department of Chemistry, University of Burgos, Burgos 09001, Spain; § Department of Integrated Biosciences, Graduate School of Frontier Science, The University of Tokyo, Kashiwa 277-8562, Chiba, Japan; ∥ Division of Biomaterial Sciences, Institute of Agrobiological Sciences, National Agriculture and Food Research Organization, Tsukuba, Ibaraki 305-8634, Japan; ⊥ Department of Veterinary Medicine, 19031 University of Teramo, Teramo 64100, Italy

## Abstract

Anhydrobiosis, the ability of organisms to survive extreme conditions such as desiccation, may represent a sustainable alternative to liquid-nitrogen (liquid nitrogen)-based cryopreservation once it can be induced in mammalian cells and germplasm. While natural xeroprotectants (e.g., signature peptides of Late Embryogenesis Abundant proteins and tardigrade disordered proteins, and vitamin E) can stabilize cells under drying, their use is limited to the laboratory level due to the high cost of extraction and the incomplete understanding of the molecular mechanisms. Here we employed a multiscale modeling framework spanning quantum mechanics (QM) and molecular dynamics to investigate how these representative xeroprotectants interact with mammalian model membranes (POPC/DMPC) under desiccation and thermal cycling. We found that peptide-based xeroprotectants remain near headgroups and form dehydration-enhanced hydrogen bonds, whereas vitamin E partitions into the acyl chain region. This is consistent with suppressed pore formation in oxidized membranes. Across dry states, xeroprotectants generally increase acyl chain order and membrane rigidity, attenuate area shrinkage, and preserve bilayer thickness homogeneity. Notably, at higher peptide concentrations, chain overordering is mitigated, suggesting a tunable window for formulation. These results provide physicochemical guidelines for designing cost-effective, eco-friendly, and energy-efficient formulations of xeroprotectants and support the rational development of room-temperature preservation strategies with improved translational potential.

## Graphical abstract

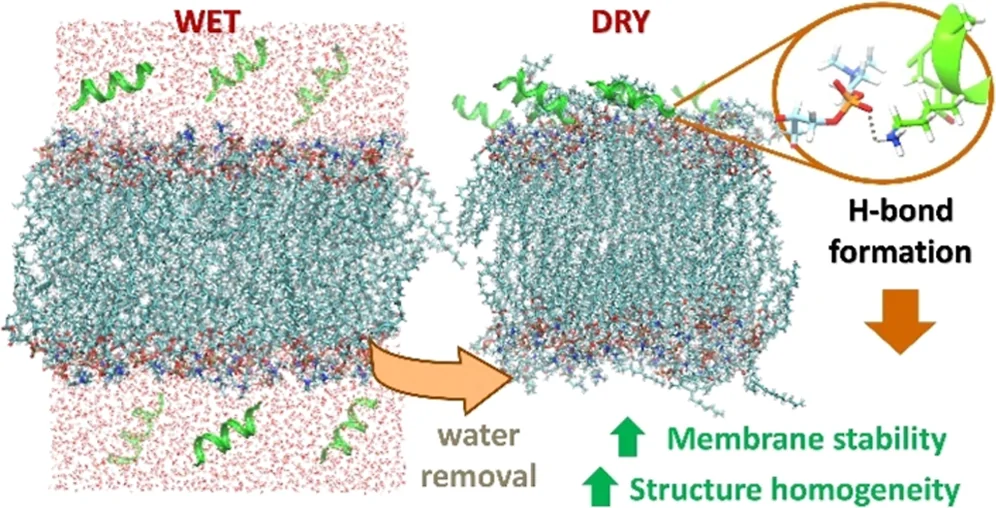

## Introduction

Long-term preservation of mammalian cells and gametes is a cornerstone of modern biomedicine, with cryopreservation in liquid nitrogen (LN_2_, −196 °C) serving as the current gold standard.^1,2^ However, reliance on LN_2_ poses several challenges: high operational costs, dependence on continuous energy and LN_2_ supply, biosafety risks, and a substantial environmental footprint.^3^ In fact, LN_2_ is relatively costly, with costs ranging from 0.13 dollars per liter for on-site production to 1.78 dollars per liter for bulk deliveries.^4^ In addition, commonly used cryoprotectants like DMSO and glycerol, essential for protecting samples during freezing,^5,6^ typically cost 50–200 dollars per liter and can be toxic at high concentrations.^7^ In the same vein, LN_2_ currently has an estimated climate footprint of 0.06 kg CO_2_e/kg.^8^ These issues highlight the urgent need for alternative preservation strategies that are both cost-effective and sustainable.

Despite the technological maturity of cryopreservation, numerous challenges still hinder its practical implementation, including cellular damage during freezing and thawing, limited scalability and reproducibility,^9^ the trade-off between loading efficiency and intracellular stress,^10^ or the long-term functionality.^11^ Natural sugars such as trehalose are the most studied chemical compounds in cryopreservation but suffer from several drawbacks, including poor membrane permeability,^12^ osmotic stress at high concentrations,^13^ incomplete protection in complex systems,^14^ and variable efficacy across cell types.^15^

In this context, anhydrobiosis, the natural ability of some organisms (such as animals, fungi, and plants) to survive in a completely desiccated state for decades without loss of viability,^16–18^ represents a promising and environmentally friendly alternative to conventional cryopreservation strategy. For instance, Late Embryogenesis Abundant (LEA) proteins, present in both plants and animals, play a crucial role in anhydrobiosis. Interestingly, their structure undergoes a reversible transformation from disordered to α-helices due to dehydration. LEA proteins protect cellular structures by stabilizing proteins and membranes, preventing aggregation, and maintaining cellular integrity in the absence of water.^19–21^ In practice, human liver cell proliferation during 1 week showed an 18-fold increase for cells dried with LEA proteins, which was only 33% lower when compared with the nondried control experiments.^22^ Similarly, tardigrades, microscopic invertebrates renowned for surviving extreme environmental conditions,^23^ synthesize tardigrade-specific intrinsically disordered proteins (TDPs) that undergo to disorder-to-order transitions upon desiccation like LEA proteins. This forms amorphous glass-like matrices that prevent the cells from aggregation or denaturation.^24,25^ Indeed, expressing CAHS, a TDP, in insect cells increased viability by approximately 50% under hyperosmotic stress.^26^

Low-molecular-weight compounds also function as protective agents. Vitamin E, for example, is a lipid-soluble antioxidant that stabilizes lipid bilayers and prevents lipid peroxidation. It also scavenges reactive oxygen species generated during drying and rehydration stress. Thus, vitamin E prevents the damage to dried tissues, liposomes, and protein formulations.^27–29^ Its hydrophobic, thermostable nature allows it to localize within bilayers of the membrane, enhancing the fluidity and preventing phase separation or collapse during desiccation.^30^ Vitamin E acts synergistically with other xeroprotectants as trehalose, of which vitrification behavior complements its membrane-stabilizing role.^31^ Maintaining cell survival through desiccation is challenging and risky, facing three major hurdles: (i) developing a technological platform for controlled water removal; (ii) formulating cell type-specific combination of xeroprotectants (from Greek, xero = dry); (iii) overcoming the permeability barrier of cellular membrane to xeroprotectants. A critical knowledge gap remains in understanding how xeroprotectants interact with cellular membranes to maintain structural and functional integrity during drying and rehydration. The lipid bilayers of mammalian cells are highly sensitive to temperature changes.^32,33^ For instance, prototypical phospholipid bilayers undergo transitions from a fluid or disordered phase at room temperature to ripple and ordered/gel phases upon cooling.^34–36^ While biological systems employ complex mixtures of xeroprotectants to stabilize membranes and proteins during desiccation and thermal stress, the molecular-level mechanisms underlying this protection have yet to be fully elucidated. In particular, research into desiccation membrane stabilization mechanisms in mammalian cells has not advanced, as these cells lack the ability of anhydrobiosis. In this regard, atomistic simulations have proven to be a powerful tool for shedding light on the interactions between the xeroprotectants and the cell membranes, thereby clarifying the physicochemical basis of desiccation tolerance. More in detail, classical molecular dynamics (MD) simulations have been widely employed to reproduce how biological membrane systems respond to dehydration and other stress phenomena (i.e., temperature). Such studies have revealed that dehydration causes lipids to pack more tightly,^37^ acyl chains to straighten,^38^ headgroup to reorient,^39,40^ gel/ordered phases to dominate, lipid dynamics to decrease,^37^ and lipid domains to redistribute.^41^ Regarding theoretical studies of xeroprotectants, Sakurai et al. demonstrated that hydrophilic interaction between GLEA3 protein chains promote the formation of a left-handed α-helical coiled coil in the dry state, thus confirming the desiccation-induced aggregation.^42^ Similarly, Boonnoy et al. confirmed vitamin E exerts antioxidant activity by trapping the polar groups of the oxidized lipids,^43^ whereas other works examines how trehalose/glucose modulate the structural properties of human cell membranes.^44,45^ Similarly, other theoretical works on the stabilization effects on dehydrated bilayers have demonstrated that certain molecules, such as trehalose, glucose, and DMSO preserve the membrane structure by replacing original water hydrogen bonds (H-bonds) or altering hydration patterns.^46–48^ To our knowledge, no systematic computational screening of xeroprotectants in mammalian membranes has been done until date.

To address this gap, we implemented a multiscale modeling platform that incorporates Quantum Mechanical (QM) calculations on molecular systems and classical atomistic MD simulations at the nanoscale. This platform has two primary objectives: (i) to provide a detailed physicochemical picture of the mechanisms by which current xeroprotectants protect membranes against desiccation and (ii) to screen representative agents from each xeroprotectant family to identify and optimize their protective properties in terms of physico-chemistry. This workflow combines the accuracy of QM-level electrostatics and statistical thermodynamics with dynamic and realistic heterogeneous membrane models, thereby capturing solute–membrane interactions beyond simple partitioning models. Similar computational strategies have been successfully applied to assess the dermatologic toxicity of natural solvents by analyzing their interaction with realistic membrane models of skin cell lines.^49^ As a realistic representation of mammalian cells, we modeled lipid bilayers composed of 1-palmitoyl-2-oleoyl-*sn*-glycero-3-phosphocholine (POPC) and 1,2-Dimyristoyl-*sn*-glycero-3-phosphocholine (DMPC), containing 16:0/18:1 and 14:0/14:0 acyl chains, respectively. Indeed, POPC and DMPC are the most abundant phospholipids in mammalian cells^50^ and are widely used in membrane-biophysical studies due to their well-characterized properties, extensive experimental data as a benchmark, low phase transition temperatures, and its ability to form stable bilayers.^51^ Moreover, their different chain lengths and packing densities allow us to explore complementary fluidity profiles.^52^ Nevertheless, it is important to highlight that POPC/DMPC bilayers are minimal mechanistic models intended to capture fundamental membrane–xeroprotectant interactions rather than reproduce the full complexity of biological membranes, which often integrate cholesterol groups, lipid asymmetry, membrane proteins, and heterogeneous lipid domains. Thus, we investigated three types of representative xeroprotectants: the group 3 LEA (G3LEA) proteins,^53,54^ heat-soluble TDPs,^55^ and vitamin E.^27^ For G3LEAs, we modeled a series of 11-mer motif peptides designed from different anhydrobiotic organisms: PvLEA (from *P. vanderplanki*), NeLEA (from nematodes), and PlLEA (from plants),^56,57^ along with a control peptide having the same amino-acid composition as PvLEA but a scrambled sequence.^58^ As representative models for TDPs, we adopted 11-mer motifs from Cytoplasmic and Secreted Abundant Heat Soluble (CAHS and SAHS) according to S. Woutersen et al.^59^ The corresponding amino acid sequences for the model peptides are summarized in Table S1. α-Tocopherol, the most biologically active form of vitamin E, was used to represent vitamin E, which is retained in the human body.^60^ In fact, α-tocopherol is usually used in the theoretical studies on vitamin E.^43,61^ To distinguish peptide-specific effects from those due to generic hydrophobic residues, we also analyzed an 11-mer model peptide composed solely of alanine (ALA11) containing neutral (methyl) side chains, and compared its behavior with representative LEA and TDP models (PvLEA and CAHS11, respectively). Bioinspired artificial peptides are more economical than the full-length natural proteins as xeroprotectants. Therefore, the rational design of such minimal model peptides is a crucial step toward the practical application of xeroprotectant technologies. Because full-length LEA proteins and tardigrade disordered proteins are typically much larger than the model peptides used here, the present simulations should be interpreted as representing simplified structural motifs rather than the complete molecular behavior of these proteins. Overall, the originality of this work lies in the following points: (i) it provides a systematic comparative screening of representative xeroprotectants from multiple families under identical membrane, hydration, concentration, and thermal conditions; (ii) the development of a coupled multiscale workflow which integrates QM-based permeability calculations and explicit atomistic MD simulations of the membrane interface, meanwhile reproducing complex phenomena such as dehydration, concentration effects and thermal cycling; (iii) the investigation of membrane interfaces with minimal motif-based peptide models, which allows isolation of sequence-dependent physicochemical effects relevant for rational peptide design to enhance their interaction with the membranes; (iv) it provides mechanistic design principles which, apart from investigating solvent dynamics and membrane permeability, are also able to identify distinct membrane stabilization mechanisms under dehydration and thermal stress.

In a first stage, we assessed the permeability of each xeroprotectant across the model membranes by relying on electrostatic potential landscapes. Subsequently, we implemented simulations reproducing the interactions of the xeroprotectants and the mammalian cell membrane under varying stress conditions, including hydration level, temperature, and concentration. These simulations allowed us to derive design principles to enhance the shielding efficiency of the xeroprotectants and elucidate the molecular mechanisms underlying their protective behavior.

## Methodology

### Electrostatic Potential Membrane Permeability Calculations

The model peptides were built based on the amino acid sequence reported in Table S1 by using the ChimeraX software.^62^ Due to the large size of the model peptides considered here, their geometries were optimized at the density functional theory (DFT) level by employing the Perdew–Burke–Ernzerhof (PBE) functional,^63^ including the Periodic Boundary Conditions and employing a combination of Gaussian and plane wave basis set as implemented in CP2K package.^64^ To avoid interactions between periodically repeated images of the xeroprotectants, the size of the unit cell was set to ensure a minimum vacuum distance of 10 Å between neighboring molecular systems. The calculations were carried out with a Double-zeta Valence Polarized and an energy cutoff for the auxiliary plane wave basis equal to 500 Ry, whereas the core electrons were described by means of Goedecker-Teter-Hutter (GTH) pseudopotentials.^65^ Afterward, the electrostatic potential (ESP) of the resulting structures was estimated in the DFT framework within the COnductor-like Screening MOdel for Realistic Solvents model^66^ by relying on the BP86 functional within a Triple-Zeta Valence Polarized basis set, as implemented in Gaussian16 package.^67^ The computed ESP files were thus employed to evaluate the interactions of these xeroprotectant molecular systems with the homogeneous lipidic cell membranes by adopting the COSMOperm model,^68^ as it has been developed in COSMOtherm software (version 24.1.0). All thermodynamic permeability properties were calculated at room temperature (*T* = 300 K).

### Molecular Dynamics Simulations

To finely reproduce the structural properties of mammalian cells embedded in solvated environments, we build a solvated interface of a mammalian phosphatidylcholine bilayer model (POPC/DMPC) commonly used as a model of mammalian membranes. This model consists of lipid bilayers with two leaflets containing 64 lipids per leaflet, and by considering 50 water molecules per lipid group to mimic the solvation environment. The resulting simulation box presented cell dimensions of 6.4 × 6.4 × 10 nm, and it is represented in Figure S1. These lipid bilayer simulation cells have been probed to fairly reproduce the structural behavior of representative mammalian cells in previous theoretical works.^69–72^ These models, together with their corresponding force field (FF) parameters, were constructed by employing the Membrane Builder module of CHARMM-GUI program.^73^ Afterward, in order to build the interface of the membranes solvated with the distinct xeroprotectant agents, we introduced a given number of molecular systems in order to reach a concentration about 5 wt % in water, which can be representative of the presence of such systems in aqueous media. We relied on the FF converter module of CHARMM-GUI,^74^ to automatically assign the CHARMM36 FF^75^ parameters from both xeroprotectants and phospholipids, whereas the TIP3P model was used to treat water molecules. The geometries of the resulting solvated cells were first relaxed at the molecular mechanics (MM) level by using a steepest descent algorithm until the computed energies reached a cutoff equal to 100 kJ/mol/nm. Once the interfaces between the lipid bilayers and the solvated xeroprotectants have been relaxed at the MM level, the desiccation process is reproduced by randomly removing a certain percentage of water molecules (0, 10, 25, 50, 75 and 95%) from the interface, being the resulting dried interfaces relaxed again by MM simulations. It is noteworthy to mention that water removal protocol based on random deletion of water molecules to reproduce dehydration processes presents certain limitations, such as the lack of a real drying pathway (i.e., diffusion and evaporation at the interface), the disruption of hydrogen-bond networks, loss of water structuring near interfaces, the introduction of nonequilibrium and structural rearrangement artifacts, or lack of control of drying kinetics. However, this protocol was selected because it enables systematic comparison across multiple xeroprotectant families under controlled hydration conditions; further extensive equilibration steps were performed after water removal to reduce transient nonequilibrium artifacts and, more importantly, similar drying MD protocols have been successfully applied to track the membrane structural changes upon water removal, being further corroborated via experimental measurements.^37^ For the setup of the equilibration and production runs we adopted the simulation protocol developed by E. Lindah et al. refined to fit cryogenic electron microscopy membrane densities.^76^ The equilibration procedure consisted of six simulation steps where certain restraints to the lipid geometries were applied and gradually removed to avoid undesired changes in system size and conformation. The first two *NVT* equilibration steps consisted of 150 ps simulation runs with a time step of 1 fs where all velocities were assigned based on a random Maxwell–Boltzmann distribution at 300 K, and the temperature kept constant through the velocities rescale thermostat of Bussi et al.,^77^ with a coupling constant equal to 1 ps. The remaining four equilibrations steps were conducted in the *NPT* ensemble at 300 K and 1 bar, coupling the thermostated system to a semi-isotropic C-rescale barostat,^78^ with a coupling constant of 1 ps and compressibility value of 4.5 × 10^–5^ bar^–1^. The first *NPT* equilibration run was pursued during 150 ps with a time step of 1 fs, the second and the third for 1 ns, and the fourth one during 20 ns with time steps equal to 2 fs. Finally, for the *NPT* production runs all geometrical restraints were completely lifted and were performed within the same thermodynamic conditions as before during 100 ns with a time step of 2 fs. For carrying out the quenching annealing process, we relied on the protocol employed in ref 44 where the system initially equilibrated at 350 K was gradually cooled up to 250 K in steps of 10 K. Although the initial temperature (350 K) exceeds physiological conditions, it was selected to ensure complete equilibration within the fluid membrane phase, avoid trapping the systems in metastable ordered states, and reproduce previously established membrane annealing protocols from the literature.^44^ *NPT* productions runs of 100 ns were performed for each temperature step, thus leading to a total annealing simulation run of 1.1 μs. Note that this cooling protocol was intended to probe membrane structural responses to thermal stress under varying hydration levels, rather than a full molecular model of cryopreservation since these models neglect ice nucleation, crystallization, osmotic freezing effects, or water phase transitions. For all MD simulations short-range electrostatic and van der Waals cutoff distances were set to 1.2 nm, whereas long-range electrostatic interactions were accounted by means of the Particle Mesh Ewald method.^79^ The resulting trajectory was stored every 50 ps and used for a further analysis. In this context the membrane thickness and the lateral area occupied per lipid were computed by relying on the GridMAT-MD module.^80^ All reported quantities were averaged over equilibrated trajectory segments, and time-averaged properties were monitored for stability during production runs. Statistical errors are provided for the main quantities estimated in the Tables of the text.

## Results and Discussion

The multiscale approach that we have developed to evaluate the membrane-level stabilization mechanisms of the distinct xeroprotectants displayed in Figure 1 comprises the following steps: analysis of the permeability of the xeroprotectants into the membranes, screening of the xeroprotectants in realistic xeroprotectant/membrane interfaces, study of the influence of the xeroprotectant concentration, and investigation of the combination of desiccation and thermal cycles into the xeroprotectant performance.

**Figure 1:**
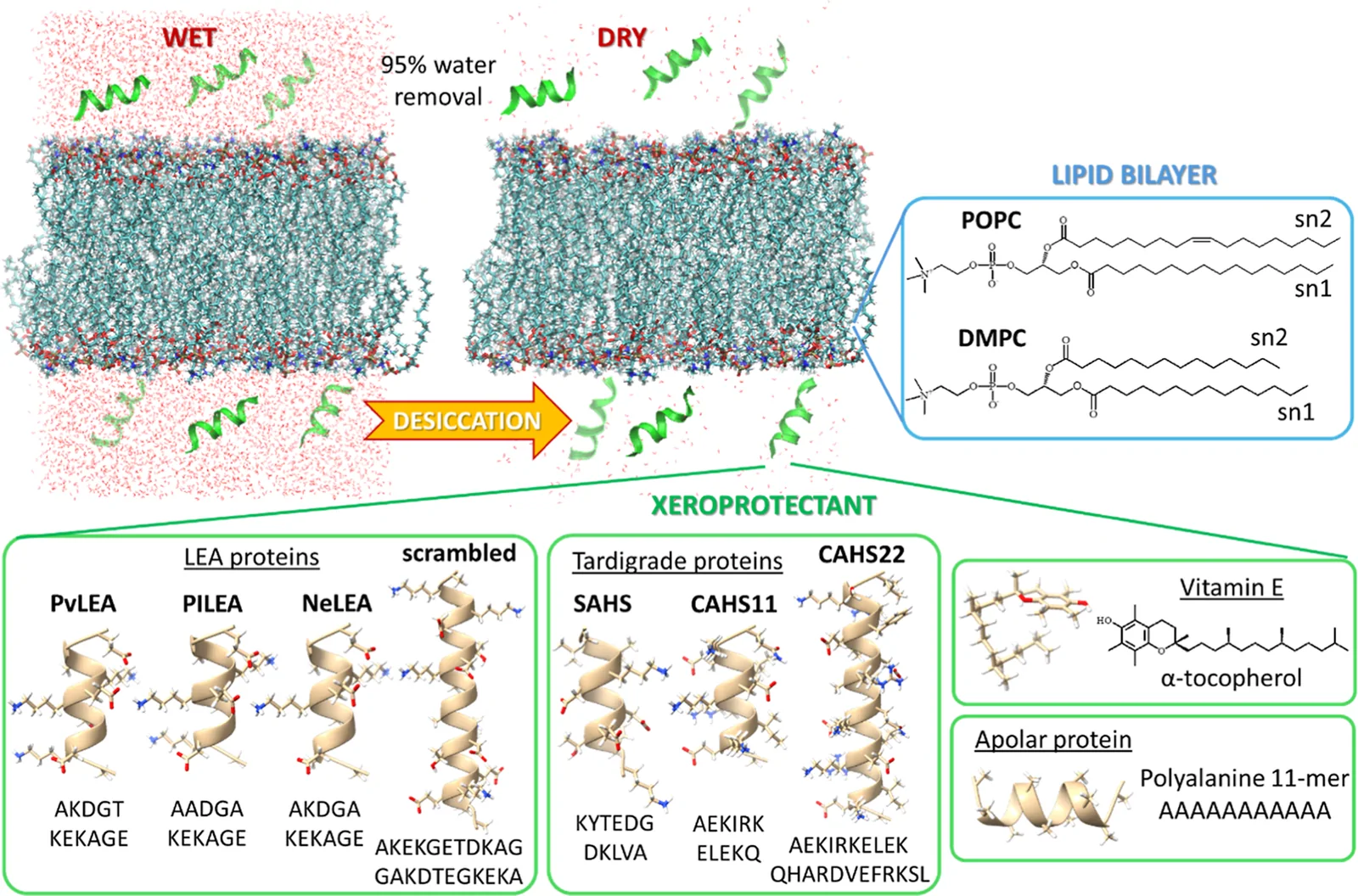
Lateral views of the solvated xeroprotectant/lipid bilayer cell membrane interfaces in their fully solvated (wet) and desiccated (dry) environments. The chemical structures of the POPC and DMPC lipid groups composing the lipid bilayers including the nomenclature of their sn1 and sn2 acyl chains; and the atomistic representation of the xeroprotectants with their respective amino acid sequence for the model peptides; are depicted in the squares delimited with blue and green colors, respectively.

### Xeroprotectant Permeability into Mammalian Cells

We started our analysis by evaluating the permeability of the xeroprotectant molecular systems into the two prototype bilayers (POPC and DMPC) which are able to capture key structural and electrostatic features relevant for mammalian cells, such as headgroup charge, hydration, and membrane fluidity. To this aim, we adopted the COSMOperm technique, which employs COSMO-RS-derived chemical potentials across membrane segments to compute position-dependent diffusion coefficients and partitioning, enabling the calculation of passive permeability coefficients through inhomogeneous lipid bilayers based on solute–membrane free energy profiles. The first property that can be accessed with this approach is the probability distribution of the molecular systems across the membrane depth, which provides an estimation of the most stable location where the model peptides/molecules will be found when crossing the lipid layers. The distributions of the distinct LEA, TDPs and vitamin E families as a function of the membrane penetration depth are presented in Figure S1. Notably, all peptide-based xeroprotectants (LEA and TDPs) lie in the areas near the phospholipid headgroups, being PvLEA and CAHS22 the ones penetrating in a lower extent into the membrane and being located on the vicinity of the headgroup side chains. On the contrary, vitamin E locates along the areas close to the phospholipid acyl chains, being this distribution broader with respect to LEA and TDP xeroprotectants. As a matter of fact, this can be explained due to both the small size of the α-tocopherol molecules and the lack of high polar groups in their chemical structures, which facilitate smooth penetration into membranes. This result is consistent with previous works which demonstrated that the deep embedding of the α-tocopherol into the lipid bilayers enables the interaction with oxidized lipid groups, thus suppressing pore formation in lipid membranes.^43^ Interestingly, this picture is in agreement with the free-energy profiles computed along the membrane depth, which are depicted in Figure S2. In the case of peptide xeroprotectants, the free energy minima is located in the area surrounding the phospholipid headgroups, thus being necessary to overcome an energy barrier in the order of 30–110 kcal/mol to fully penetrate into the membrane. On the other hand, free energy profiles of vitamin E are mostly flat and decreased in energy when approaching the acyl chains. As a result, the diffusion of the xeroprotectants across the membrane differs significantly depending on the family, being the diffusion of vitamin E 1 order of magnitude higher with respect to the rest of the peptide systems (see Figure S3). Finally, we have computed the permeability values for the entire series of xeroprotectants, which are collected in Table 1. Due to their larger size, the peptide xeroprotectants displaying the lowest permeabilities for LEA and TDP families corresponded to the scrambled and CAHS22 systems, respectively. Then, as a general trend, LEA peptides exhibited lower permeability values with respect to TDPs, which can be attributed to the presence of more charged/polar residues in their amino acid sequence. In line with previous findings, vitamin E displayed the largest permeability, showing even positive values, consistent with strong partitioning of α-tocopherol into the hydrophobic region of POPC/DMPC bilayers within the COSMOperm framework. Only in the case of 11-mer TDPs, DMPC permeability values were found slightly lower when compared with POPC, whereas for the rest of the systems no significant differences between the two types of lipid layers were found.

**Table 1:** Computed Values in Logarithmic Scale for the Permeability of LEA Peptides, TDPs, Vitamin E and Reference Apolar Peptide (ALA11) into the POPC and DMPC Membranes, as Estimated by Following the COSMOperm Approach

	Log P (cm/s)	
name	POPC	DMPC
PvLEA	–28.1	–29.6
PlLEA	–28.8	–26.8
NeLEA	–26.3	–29.9
scrambled	–31.6	–27.0
SAHS	–23.7	–19.9
CAHS11	–21.7	–16.8
CAHS22	–29.1	–30.1
vitamin E	1.6	1.6
ALA11	–23.0	–16.5

Subsequently, we compared the thermodynamic permeability properties of two representative xeroprotectant peptides (PvLEA and CAHS11) with those from the reference apolar peptide ALA11 (see Figure S4). Interestingly, ALA11 peptides showed similar probability distribution and free energy profiles as the ones found for CAHS11. However, the lack of polar groups inducing an electrostatic repulsion with the membrane headgroups enhances the diffusivity of ALA11 along the membranes, leading to diffusivities in the areas close to the headgroups, which were around two times larger with respect to the xeroprotectant peptides (see Figure S4 bottom). Due to this, the permeability of ALA11 was found to be consistently higher than the ones computed for the xeroprotectant peptides, thus corroborating that specific polar groups from xeroprotectants peptides hinder their penetration into the mammalian cells, thus maintaining their integrity. In summary, whereas penetration of vitamin E into DMPC and POPC bilayers was found spontaneous, high-energy barriers were obtained for peptide penetration into the membrane. Then, to further evaluate the consequences of these energy landscapes in the membrane structures, we conducted MD simulations at finite temperatures. Note that the cellular uptake of exogenous compounds is governed by multiple processes—including adsorption, endocytosis, aggregation, membrane perturbation, concentration effects, and charge state—and that passive permeation through lipid bilayers represents only one of these mechanisms. Therefore, while the high membrane permeability predicted for vitamin E may contribute to its efficient cellular incorporation, the uptake of peptide-based xeroprotectants likely involves additional mechanisms beyond simple membrane permeation. In addition, it is important to highlight that while POPC and DMPC homogeneous bilayers provide well-established model systems to investigate fundamental membrane–solute interactions and permeability mechanisms, we acknowledge that mammalian cell membranes display significantly more complex lipid compositions and heterogeneity; therefore, our results should be interpreted as mechanistic insights at the molecular level, and further experimental validation in biological membranes will be necessary to fully confirm the behavior of vitamin E in cellular environments. Before closing this section, we should stress that semiempirical QM-permeability calculations provide complementary descriptors that help rationalize the membrane interaction mechanisms observed at the atomistic MD level (see next sections).

### Screening of Xeroprotectant Families in Wet and Dry Environments

Note that all results presented in the previous section were obtained by relying on an implicit treatment of chemical potentials, as computed at the semiempirical QM level. Nonetheless, to get a complete picture of the effect of xeroprotectants on the membrane structure during the dehydration process, one should rely on a full atomistic explicit consideration of both membrane and xeroprotectants in both wet and dry environments.^81^ To fill this gap, we conducted a series of MD simulations at room temperature for the different xeroprotectants depicted in Figure 1 in two types of interfaces differing on their water content: the wet environment with the fully solvated interfaces and the dry environment where 95% of the initial water molecules were randomly removed (see Figure 1 top). Note that these simulations should be considered as controlled computational perturbations aimed at probing how reduced hydration alters membrane–xeroprotectant interactions, rather than as exact representations of experimental drying pathways. In addition, with the aim of getting a reference of the intrinsic behavior of the membranes during the drying process, we carried out control simulations where no xeroprotectants (only water molecules) were present in the interface with the lipid bilayers. We have started our analysis by computing the density profiles along the membrane normal direction for the distinct groups integrating the interface: membrane components (headgroups, glycerol, acyl chains), xeroprotectants, and water molecules; in order to track their average position during the simulation run. The density profiles of the interfaces with some representative xeroprotectants of each family, the apolar peptide, and the control simulations are depicted in Figure 2. In the wet scenario (Figure 2a) the lack of overlap between the peptide and membrane headgroup distribution evidenced the absence of peptide penetration during the simulation. Similarly, no overlap between the water solvent and the hydrophobic acyl chains was observed. Indeed, the similar membrane component distributions found in the peptide vs control simulations evidenced the absence of a major impact of the presence of the xeroprotectant on the intrinsic membrane structure. Thus, when moving to the dry environment (Figure 2b) the distribution of the membrane components was found broader than in the wet scenario, thus indicating an increase in the structural disorder within the lipid layers. Nevertheless, the shape and characteristics of the membrane distribution were largely preserved upon the drying process, thus corroborating the integrity of the lipid bilayer structure. Interestingly, the dehydration process led to an enhancement of the overlap between peptide and membrane headgroup distribution, thus evidencing a stronger interaction between the xeroprotectant and the membrane upon water removal. This strong xeroprotectant–membrane interaction can be easily observed in the last snapshots of the simulations performed in the presence of PvLEA and vitamin E within the dry scenario depicted in Figure S5, where xeroprotectant molecular systems remained in close contact with the membrane headgroups. Moreover, the differences between the distinct xeroprotectant types and between the peptide and control simulations were more marked in the dry environment. As a matter of fact, similar density profile characteristics were observed in both wet and dry environments for the different LEA (see Figure S6) and TDPs (Figure S7) xeroprotectants studied here. Then, with the aim of verifying that our results are robust against the randomness of the geometrical space explored during the MD simulation, we estimated the density profiles along the membrane direction over two-halves of the trajectory (0–50 ns, 50–100 ns), which are presented in Figures S8 and S9. Notably, the similar trends in the density profiles observed within the distinct xeroprotectant families, the apolar protein and water, with respect to the full trajectories clearly evidenced the reproducibility and consistency of our results.

**Figure 2:**
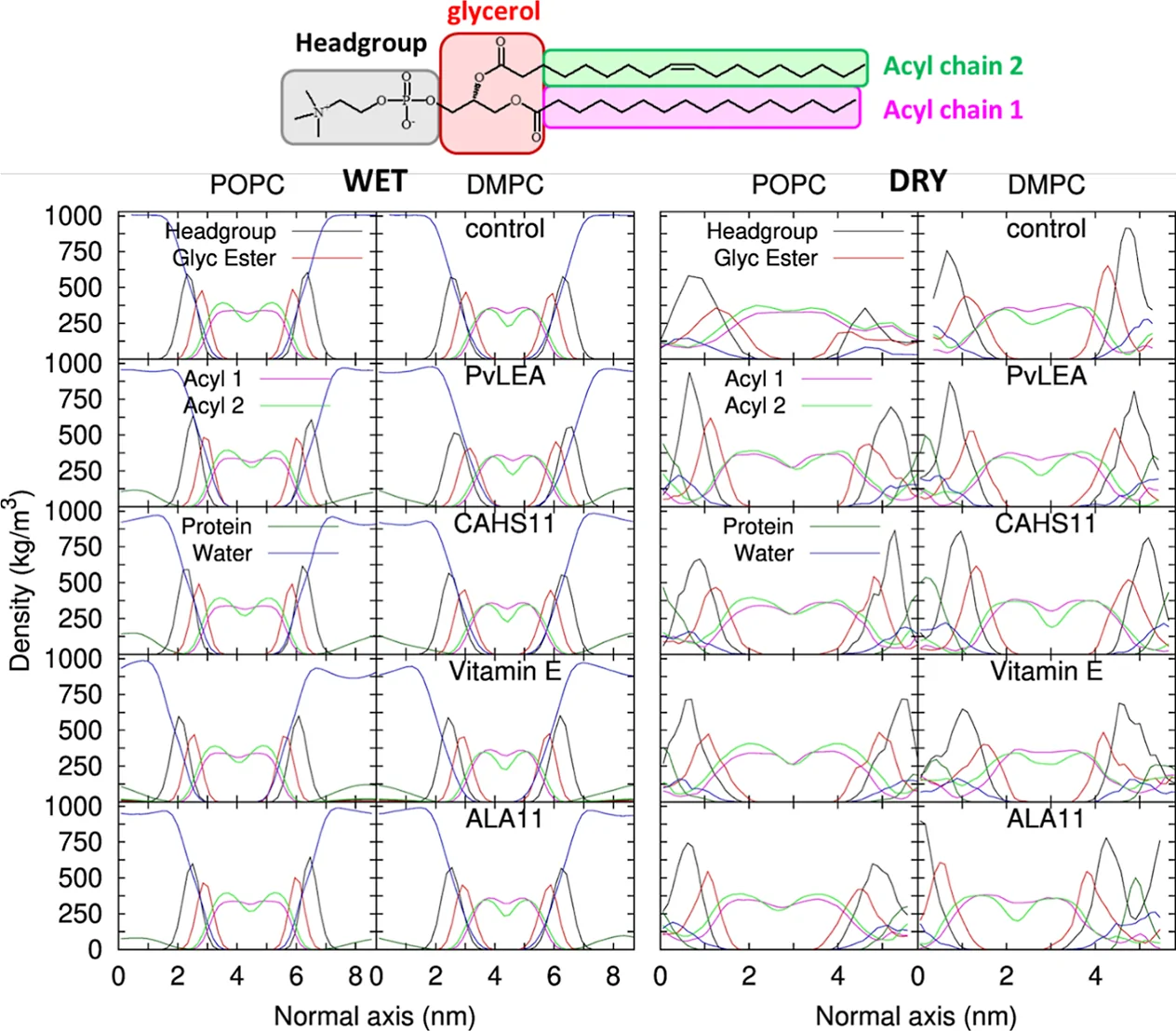
Average density profiles along the normal direction to the membrane plane of the POPC and DMPC lipid bilayer (phospholipid headgroups, glycerol ester, two acyl chains) and water solvent of the membranes embedded in pure water (control) and solvated representative xeroprpotectant (LEA peptides, TDPs, vitamin E and apolar peptide) environments, within their respective fully solvated (left) and dry (right) scenarios. All simulations were performed at room temperature (*T* = 300 K). A schematic representation of the different parts of the phospholipid groups conforming the lipid bilayers is presented at the top of the graphs.

Afterward, with the objective of further rationalizing the nature of the interaction between the membrane, the xeroprotectants and the water environment, we conducted the radial distribution function (RDF) analysis taking as reference the atoms belonging to the lipid bilayers. Figure 3 displays the RDF plots for the representative xeroprotectants of each family, the apolar peptide and the control simulations. As one may expect, in the water environment, RDF plots are dominated by the interactions with water molecules, which exhibited a marked peak located at 1.8 Å corresponding to the first solvation shell, whereas the interactions with the xeroprotectants are rather weak in the areas surrounding the membrane. On the other hand, as already pointed in the density profiles, the desiccation process resulted in an enhancement of the interaction with the membranes, not only with the xeroprotectants, but also with the solvent molecules. Furthermore, in the dry scenario, peptide models (PvLEA and CAHS11) showed a similar peak to the one exhibited by water molecules at 1.8 Å. All these features were also reproduced in the rest of xeroprotectant RDF plots (see Figures S10 and S11). Concerning the differences between lipid membranes, while both POPC and DMPC exhibited similar RDF plots in the wet environment, the enhancement of the interaction with water molecules driven by the dehydration procedure was found stronger in the case of POPC. Moreover, in order to discard that the differences among the distinct RDF plots are simple artifacts derived from random geometries, we further computed the RMSF plots divided into two simulation blocks (two-halves of the trajectory) for the representative xeroprotectants employed in Figure 3, which are represented in Figures S12 and S13, respectively. Interestingly, by comparing the RMSF plots from the full production trajectories (Figure 3) and those from the two halves of the trajectory (Figures S12,S13), one can observe that the same impact of desiccation appears in all considered RDF plots and that the RMSF variations across windows are significantly smaller than the differences between DESs.

**Figure 3:**
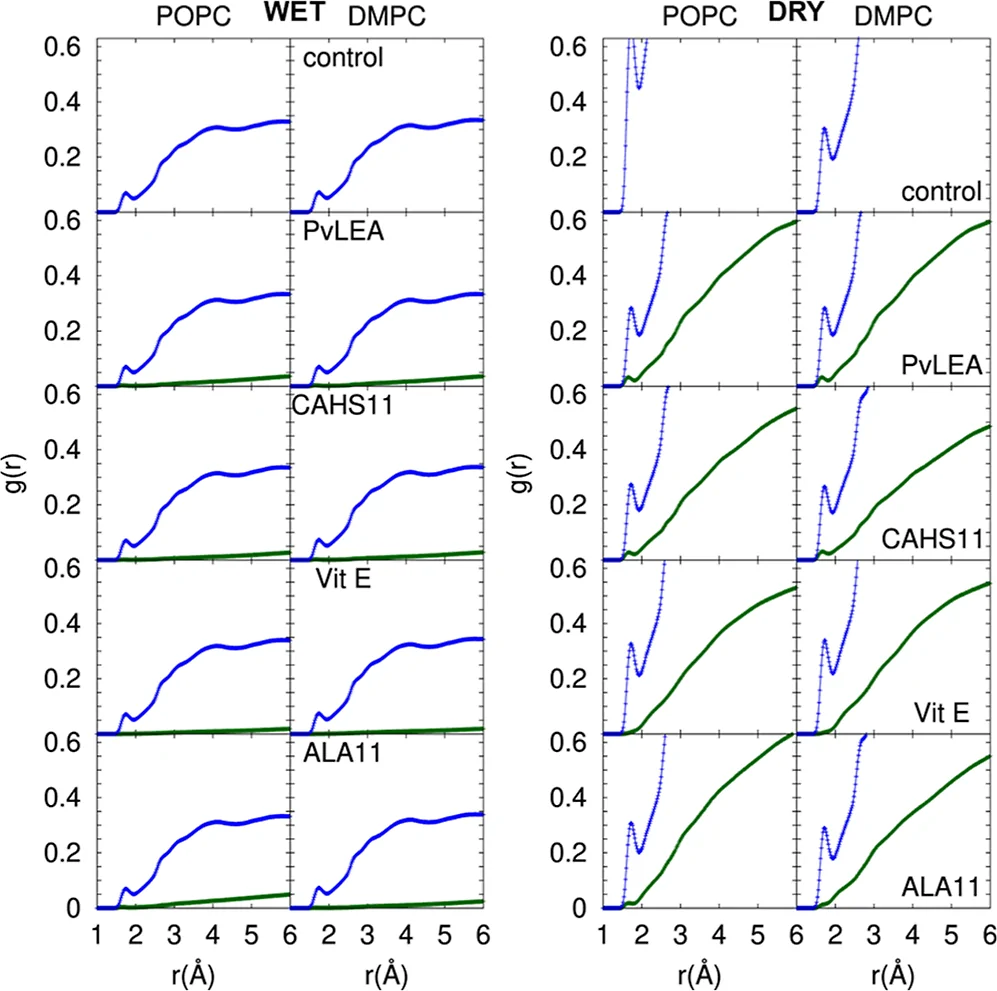
RDF plots between the water solvent molecules (blue) and the xeroprotectant (green), with respect to the atoms from the POPC and DMPC lipid bilayers for the membranes embedded in pure water (control) and solvated representative xeroprotectant (LEA, TDPs, vitamin E) and apolar peptide environments, within their respective fully solvated (left) and dry (right) scenarios. All simulations were performed at room temperature (*T* = 300 K). Only distances from the membrane atoms lower than 6 Å were plotted here.

Then, in order to get more quantitative data to characterize these interactions, we computed the average number of H-bonds formed between the membrane and the xeroprotectants and compared it with those formed with the solvent molecules (see Table 2). Interestingly, the number of H-bonds formed by the xeroprotectant in the hydrated environment is minimal (only a few), being about two or 3 orders of magnitude lower with respect to the number of H-bonds conformed by water molecules. Strikingly, when moving to the dry scenario, the number of H-bonds with xeroprotectants was boosted by 1 or 2 orders of magnitude, being now only about 1 order of magnitude lower than the number of bonds formed with the solvent. Regarding the distinct xeroprotectants, the ones yielding to a stronger interaction with the membrane in the dry scenario corresponded to PlLEA, CAHS22, and vitamin E. Furthermore, the number of H-bonds found in the apolar peptide was consistently lower than the ones observed in the rest of xeroprotectants, thus pointing to the important role played by the specific polar amino acid groups of the xeroprotectants in promoting the interaction with the membranes. Overall, our calculations demonstrated that the shielding action of the xeroprotectants is certainly screened by the presence of a water environment.

**Table 2:** Number of H-Bonds Formed between the Xeroprotectant ((*H*_b_)_mol_), the Water Solvent Molecules ((*H*_b_)_wat_), and the Lipid Groups from the POPC and DMPC Cell Membranes Embedded in Solvated LEA Peptides, TDPs, Vitamin E, Reference Apolar Peptide (ALA11) and Pure Water (Control) Environments, within the Fully Solvated (Wet) and Desiccated (dry) Scenarios^a^

		POPC				DMPC			
		wet		dry		wet		dry	
Xero	wt %	(*H*_b_)_mol_	(*H*_b_)_wat_	(*H*_b_)_mol_	(*H*_b_)_wat_	(*H*_b_)_mol_	(*H*_b_)_wat_	(*H*_b_)_mol_	(*H*_b_)_wat_
PvLEA	5.5	1.6	918	11.4	368	1.1	877	8.1	342
PvLEA	25.9	1.8	904	27.8	334	4.0	864	28.7	318
PlLEA	5.1	0.5	914	75.0	121	1.3	873	63.4	121
NeLEA	5.4	0.5	919	20.5	327	2.9	885	15.4	302
scrambled	7.2	0.8	918	29.1	331	1.3	886	26.0	310
SAHS	6.0	2.3	913	22.8	306	1.3	889	24.4	306
CAHS11	6.6	1.1	918	19.6	311	1.5	884	18.5	318
CAHS22	8.5	2.6	914	34.7	318	1.0	890	38.7	303
Vitamin E	5.6	0.1	923	34.6	298	0.1	890	41.0	297
ALA11	4.0	1.4	918	5.5	366	0.4	894	5.3	380
control	0	-	921	-	368	-	896	-	373

a All these simulations were conducted at room temperature (*T* = 300 K). The standard deviations for the average magnitudes presented here are reported in Table S2.

Nonetheless, a rational design of the xeroprotectant chemical structure requires discerning which are the specific chemical groups holding a stronger interaction with the membrane cells. To fill this gap, we have disentangled the number of H-bonds formed between the membrane cells and the distinct amino acid residues integrating the 11- and 22-motif peptide models, as it is represented in Figures S14 and S15, respectively. Strikingly, for all LEA 11-mer peptides, amino acid residues 2, 6, and 8 exhibited the largest number of H-bonds, which in almost all cases corresponded to the lysine amino acid group (K in the peptide sequence). Likewise, for TDP 11-mer peptides residues 1 and 8 for SAHS; and residues 3, 5, 6, and 10 for CAHS11 showed the strongest interactions with the membrane which, again, correlated with lysine groups. In the case of the apolar peptide, only the side alanine groups display a significant interaction with the membrane. For the 22-mer peptides, the most pronounced interactions were shown by residues 4 and 19 for the scrambled LEA and by residues 6, 10, 14, and 19 for CAHS22, which are conformed by lysine and arginine groups. Therefore, we can conclude that the xeroprotectant behavior of LEA and TDP peptides is related to their high content in amino acids containing primary amino groups.

In a second stage, we have evaluated the impact of the drying process on the structural features of the membranes, and the role played by the presence of the xeroprotectants in those properties. With this aim, we have subtracted a series of geometrical descriptors of the membrane structure from the MD trajectory snapshots.^82,83^ In this viewpoint, such descriptors as acyl-chain order, bilayer thickness, membrane compressibility, and hydrogen bonding are widely used membrane biophysics descriptors associated with membrane stability under dehydration stress,^84,85^ which should not be interpreted as direct predictors of cellular survival or viability. The first one of these descriptors are the deuterium order parameters (*S*_CD_), which provide an estimation of the alignment of the lipid acyl chains (sn1 and sn2 in Figure 1) with respect to the direction normal to the membrane bilayer, and have been calculated by following this formula

1 $$S_{CD}=\frac{1}{2}\left\langle 3\cos^{2}\theta -1\right\rangle$$

where θ is the angle formed between the lipid groups and the normal direction to the membrane. Values close to 1 are usually related to aligned acyl chains, whereas those close to −0.5 depict antialignment. The estimated S_CD_ values for the C atoms conforming the sn1 and sn2 acyl chains from POPC- and DMPC-based membranes in the presence of the xeroprotectants in both wet and dry environments are displayed in Figure 4. In line with the density profiles from Figure 2, the distinct xeroprotectants showed identical S_CD_ values when compared with the ones from the control simulations. Therefore, we can conclude that the alignment of the lipid chains is not highly affected by the presence of the xeroprotectants in the hydrated systems. Only small deviations with respect to the S_CD_ values of the control simulations are observed in the DMPC lipid bilayers. In spite of this, that picture is completely changing in the dry environment. Concerning the control simulations, while the alignment POPC lipid chains remained almost unaffected by dehydration, DMPC chains become more aligned (ordered) upon water removal. This phenomena was previously observed in DMPC layers when removing one-third of the initial water molecules of the MD simulation.^37^ However, *S*_CD_ values increased in the dry scenario due to the interaction with the xeroprotectants, being this feature especially remarkable for the sn1 chains from POPC lipid bilayers. More in detail, the xeroprotectants yielding to the highest enhancement of the lipid chain alignment were PlLEA and SAHS for POPC, and NeLEA and CAHS22 for DMPC. Surprisingly, the behavior of acyl chains in contact with vitamin E differs as a function of the lipid bilayer considered, whereas for POPC it shows the same trend as the rest of the xeroprotectants (increase of *S*_CD_), for DMPC vitamin E decreased the *S*_CD_ values with respect to the control simulations. This could be attributed to a certain penetration of α-tocopherol molecules in the DMPC lipid bilayers during the simulations in the dry environment and can be effectively employed to hamper the formation of ordered (gel) phases in DMPC layers. As a general trend, our simulations evidenced that, in the presence of xeroprotectants, membranes undergo a more ordered structure in their dry state. Furthermore, with the aim of confirming that the trends observed in the deuterium order parameters are not artifacts associated with the specific geometries sampled during the MD trajectories, we additionally computed the S_CD_ plots over two independent simulation windows corresponding to the two-halves of the trajectories (0–50 ns and 50–100 ns), which are presented in Figures S16 and S17. Remarkably, by comparing the S_CD_ profiles obtained from the full production trajectories (Figure 4) with those derived from the two trajectory blocks (Figures S16 and S17), the same qualitative effects of dehydration and xeroprotectant family on the lipid acyl-chain ordering were consistently reproduced for both POPC and DMPC bilayers. In particular, the characteristic increase in lipid-chain alignment observed in the dry environments, as well as the distinct behavior displayed by vitamin E in DMPC membranes, remained preserved across both time windows.

**Figure 4:**
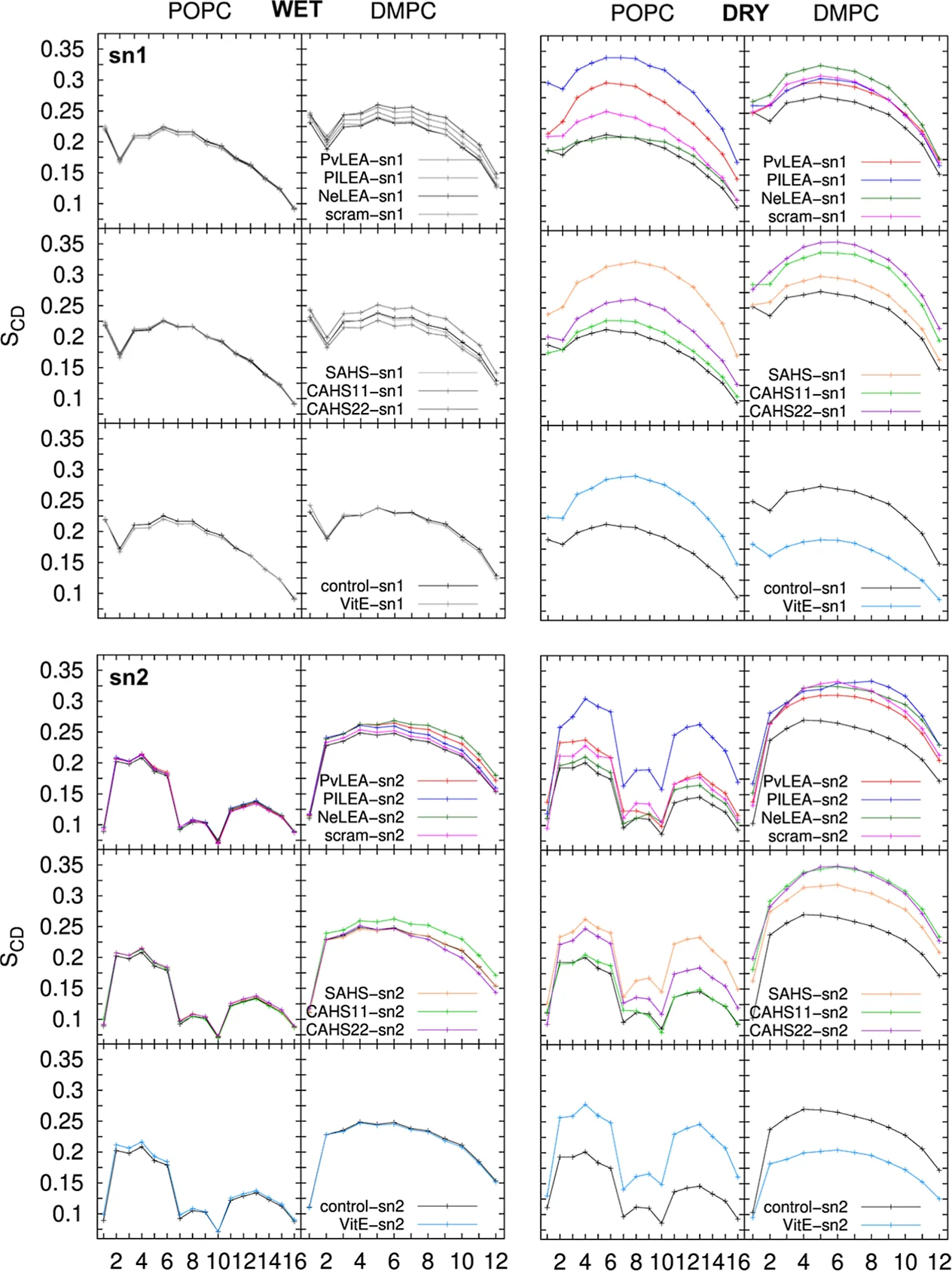
Deuterium order parameters S_CD_ of the carbon atoms belonging to two lipid acyl chains (sn1 and sn2 for top and bottom graphs, respectively) from the POPC and DMPC lipid bilayers integrating the membrane cell models embedded in pure water (control) and solvated xeroprotectant (LEA peptides, TDPs and vitamin E from the top to the bottom panels) environments, within their respective fully solvated (wet, right part) and desiccated (dry, right part) scenarios. All these simulations were carried out at room temperature (*T* = 300 K).

Other geometrical descriptors are the area occupied per lipid group (*A*_*l*_) and the diffusivity of the headgroup P atoms along the normal direction (*D*_*l*_), which are listed in Table 3. Interestingly, both POPC and DMPC lipid bilayers are contracted during dehydration, thus resulting in a decrease in the *A*_*l*_ amounting to 25–33% and 15–30% for POPC and DMPC, respectively. This goes in line with reference work which reported a decrease of 7–8% in the *A*_*l*_ when reducing by 6 the initial content of water.^37^ Whereas this membrane shrinkage is slightly mitigated due to the action of the xeroprotectants for POPC bilayers, this trend is not so straightforward in the case of DMPC. On the other hand, the mobility of lipid groups from the membrane is hampered in their dry state, resulting in diffusivity coefficients one or 2 orders of magnitude lower with respect to the fully solvated environments. This outcome is in agreement with previous works which found that all dynamic properties (diffusion, rotational, and water dynamics) were dramatically reduced when removing the water content of the DMPC bilayer.^37^ Indeed, the mobility of the membranes was further reduced in the presence of the xeroprotectants for the POPC layers, while in DMPC no direct connection between the addition of xeroprotectants and the diffusivity values of the control simulations was found. In view of this, we can ensure that, especially for POPC, membranes in their dry state become more rigid under the effect of the xeroprotectants, which in turn diminished the shrinking of the membranes triggered by desiccation.

**Table 3:** Structural Properties Extracted from the MD Simulations Performed at Room Temperature (*T* = 300 K) of the POPC and DMPC Cell Membranes Embedded in Solvated LEA Peptides, TDPs, Vitamin E, Reference Apolar Peptide (ALA11), and Pure Water (Control) Environments, within the Fully Solvated (Wet) and Desiccated (dry) Scenarios: Area Occupied per Lipid Group (*A*_*l*_), Lateral Diffusion Coefficients of the P Atoms from the Lipid Headgroups (*D*_*l*_)­^a^

		POPC				DMPC			
		wet		dry		wet		dry	
Xero	wt %	*A*_*l*_ (Å^2^)	D*10^–9^ (cm^2^/s)	*A*_*l*_ (Å^2^)	D*10^–9^ (cm^2^/s)	*A*_*l*_ (Å^2^)	D*10^–9^ (cm^2^/s)	*A*_*l*_ (Å^2^)	D*10^–9^ (cm^2^/s)
PvLEA	5.5	64.1 ± 0.8	273	48.1 ± 1.2	5.1	57.6 ± 1.2	378	50.4 ± 4.1	1.5
PvLEA	25.9	63.0 ± 1.0	270	45.6 ± 0.8	1.4	57.3 ± 1.6	56	42.7 ± 0.9	2.8
PlLEA	5.1	63.5 ± 1.8	72	46.6 ± 0.8	1.8	58.6 ± 1.2	80	48.3 ± 0.8	3.3
NeLEA	5.4	63.9 ± 1.5	261	46.6 ± 0.1	2.1	58.1 ± 1.2	105	41.8 ± 1.3	1.7
scrambled	7.2	63.5 ± 1.0	280	49.8 ± 1.6	1.2	59.0 ± 1.2	118	42.5 ± 0.8	1.9
SAHS	6.0	64.5 ± 1.3	357	48.3 ± 1.0	5.4	59.0 ± 1.1	387	47.9 ± 1.6	1.2
CAHS11	6.6	64.4 ± 1.4	189	43.3 ± 0.4	4.9	58.9 ± 2.2	218	42.4 ± 1.2	4.0
CAHS22	8.5	64.0 ± 1.2	304	47.4 ± 0.6	0.6	59.2 ± 1.0	355	40.9 ± 1.1	2.6
Vitamin E	5.6	63.9 ± 1.9	112	48.4 ± 1.2	0.5	59.2 ± 1.1	153	41.5 ± 0.5	2.3
ALA11	4.0	64.2 ± 1.1	424	47.5 ± 1.2	7.5	59.3 ± 1.2	114	44.8 ± 1.0	1.4
control	0	63.3 ± 1.3	90	43.0 ± 0.9	6.8	58.6 ± 1.5	490	47.3 ± 2.9	5.3

a The error interval values correspond to the standard deviations along the trajectory.

Another relevant structural feature of the membranes is the average thickness of the lipid bilayer. To tackle this property, we have plotted the 2-D maps corresponding to the sections formed by the normal plane to the membrane direction where each polygon domain represents the average height of each lipid group integrating the membrane layer. The 2-D thickness maps for POPC and DMPC lipid bilayers with the different xeroprotectants in both full-solvated water and dry environments are represented in Figures S18 and S19. Within the solvated water scenario, the average thickness of the lipid bilayers oscillated between 3 and 5.5 nm for POPC and 2–5 nm for DMPC. Indeed, the thickness of POPC lipid bilayers was consistently higher than that of DMPC because of their longer acyl chains. Then, when reducing the water content, the lipid bilayers become less homogeneous, reaching an average thickness of up to 2.5–6 nm for POPC and 1.5–5.5 nm for DMPC. This increase in the heterogeneity of the bilayer thickness is a fingerprint of the transition to an ordered (gel) phase of the membrane structure.^86,87^ As a general trend, the thickness of both POPC and DMPC layers overall increases upon reducing the water content, with this effect being more pronounced for POPC. Similar findings were previously reported in DMPC bilayers, where the membrane thickness was elongated by 6–9% upon removal of 83% of the initial water molecules.^37^ Interestingly, the modifications on the lipid bilayer thickness observed upon water removal are weakened by the presence of the xeroprotectants for both POPC and DMPC. Only in the peculiar case of the apolar peptide did the desiccation process lead to a significant drop in the thickness for both POPC and DMPC lipid bilayers, thus highlighting the role played by the peptide amino acid groups in preserving the membrane thickness and homogeneity upon desiccation.

We further analyzed the mechanical properties of the membranes by relying on two parameters: the lateral tension γ, which provides an estimation of the out-of-plane strain and is calculated as the conjugate to the membrane fluctuating contour area *A*; and the area compressibility modulus *K*_*A*_ which determines the membrane’s ability to compress and expand; which were estimated by employing the following formulas

2 $$\gamma =\frac{1}{2}L_{z}\left(P_{zz}-\frac{P_{xx}+P_{yy}}{2}\right)$$

3 $$K_{A}=\frac{k_{B}T\left\langle A\right\rangle }{\sigma_{A}^{2}}$$

where *L*_*z*_ represents the normal dimension of the cell, *P*_*ii*_ the diagonal elements of the pressure tensor, the *A* the projected membrane area and its corresponding variance σ_*A*_. The computed values of γ and *K*_*A*_ are collected in Table S3. When comparing the values computed in the wet and dry states of the membrane, no straightforward trends were observed in the lateral tensions γ, whereas the compressibility coefficients *K*_*A*_ were enlarged in the dry scenario due to the dehydration driven shrinking of the cells. More importantly, while for the control simulations and apolar peptides the increase in *K*_*A*_ driven by the drying process was found rather small, it was further boosted due to the action of the xeroprotectants (between 2 and 21 and 3–35 times in the case of POPC and DMPC, respectively), being CAHS11 the system with the highest enhancement of *K*_*A*_. In general, we can stand that the presence of xeroprotectants in the dry membrane’s state led to more ordered and rigid structures.

As highlighted in the introduction, the structural modifications affecting xeroprotectant agents during the desiccation phenomena have been pointed to be at the origin of their outstanding preservation in extreme conditions. In this viewpoint, we have computed a series geometrical descriptors to further characterize the structural dynamics of the xeroprotectant peptides during the simulation: the root-mean-square displacement (rmsd) of the peptide, which gives an idea of the peptide mobility along the trajectory; the radius of gyration (*R*_g_), which represents the average distance of a peptide’s atoms from its center of mass and provides an estimation of the peptide compactness; and the internal number of H-bonds formed within the peptide structure. These three quantities for the LEA, TDP and apolar peptides are presented in Table 4. Interestingly, possibly due to the enhancement of the interactions with the membrane, the mobility of the peptides is hindered under the dry conditions, leading to a decrease in the rmsd values about 2–5 times when compared with the ones from the initial water solvated scenario. Moreover, water removal induced a shrinking of all peptides, which was translated into more compact structure (i.e., in a reduction of *R*_g_ about 5–20% with respect to the wet environment), and in the formation of additional internal H-bonds within the peptide (about 1.1–2.7 times higher when compared to the hydrated systems), thus further enhancing the stability of the peptides.

**Table 4:** Structural Properties of the LEA Peptides and TDPs in the Solvated Interfaces with the POPC and DMPC Lipid Bilayers within the Fully Solvated (Wat) and Desiccated (Dry) Environments: Root-Mean-Square Displacement (rmsd) in Å, Radius of Gyration (*R*_g_) in Å, and Number of H-Bonds Formed within the Peptide Structure^a^

	POPC						DMPC					
	wat			dry			wat			dry		
Xero	RMSD	*R*_g_	H_b_	RMSD	*R*_g_	H_b_	RMSD	*R*_g_	H_b_	RMSD	*R*_g_	H_b_
PvLEA	4.98	7.59	29	1.07	6.28	79	5.22	7.63	30	1.24	6.28	75
PlLEA	4.88	7.45	27	1.05	6.05	70	4.57	7.13	29	1.19	6.05	65
NeLEA	4.21	6.99	40	1.15	6.14	79	5.42	7.83	26	1.18	6.18	69
scrambled	3.75	10.67	167	1.83	10.57	227	3.97	11.06	149	1.09	10.61	228
SAHS	4.12	6.96	54	1.36	6.63	64	4.07	7.37	45	3.13	6.90	74
CAHS11	3.55	6.74	62	1.18	6.19	105	4.14	7.16	48	1.53	6.13	113
CAHS22	3.51	10.48	176	1.39	10.42	238	3.33	10.42	181	1.43	10.46	200
ALA11	3.80	7.09	22	1.06	5.72	53	3.37	6.92	27	1.96	6.40	33

a The standard deviations for the average magnitudes presented here are reported in Table S4.

Apart from the structural characteristics of the individual peptides, the shielding effect of xeroprotectants is also influenced by the intermolecular interactions driving their supramolecular organization. In this context, we have studied the aggregation of the xeroprotectants by estimating the number and size (i.e., number of peptide/molecules conforming the cluster) of the clusters formed during the simulation run, which are presented in Table 5. As a matter of fact, both the decrease in the peptide mobility and the strength of the xeroprotectant/membrane interactions inhibit the interaction between xeroprotectants in the dry state, thus being detrimental for aggregation. Because of this, water removal produces the formation of a higher number of clusters holding a small size for all types of xeroprotectants studied here and for both POPC and DMPC layers. In view of this, our simulations evidenced that desiccation of the xeroprotectants led to more compacted individual structures but to more intercalated supramolecular organization.

**Table 5:** Aggregation Properties of the LEA Peptides, TDPs, Vitamin E Xeroprotectants in the Solvated Interfaces with the POPC and DMPC Lipid Bilayers within the Fully Solvated (Wet) and Desiccated (Dry) Environments: Number of Clusters (*N*_clust_), Averaged (*N*_avg_) and Maximum (*N*_max_) Cluster Size in Terms of Number of Molecules Integrating the Cluster^a^

	POPC						DMPC					
	wat			dry			wat			dry		
Xero	*N*_clust_	*N*_avg_	*N*_max_	*N*_clust_	*N*_avg_	*N*_max_	*N*_clust_	*N*_avg_	*N*_max_	*N*_clust_	*N*_avg_	*N*_max_
PvLEA	2.05	3.75	4.84	3.64	1.68	2.37	2.39	3.15	4.33	4.29	1.42	2.71
PlLEA	3.33	2.13	2.98	4.03	1.49	2.97	2.89	2.54	3.59	5.00	1.20	2.00
NeLEA	2.16	3.42	4.44	3.89	1.58	2.31	2.99	2.33	3.78	3.12	1.95	2.97
scrambled	1.28	3.58	3.70	2.93	1.46	2.07	1.97	2.43	3.01	1.18	3.66	3.80
SAHS	2.53	2.89	4.13	4.67	1.31	2.30	2.36	3.17	4.15	3.87	1.65	2.63
CAHS11	2.30	3.42	4.30	5.01	1.20	1.99	2.35	3.28	4.46	3.97	1.53	2.10
CAHS22	2.32	2.01	2.55	2.23	1.85	2.00	1.76	2.97	3.21	1.92	2.19	2.22
vitamin E	11.70	1.41	3.44	13.57	1.20	2.52	11.74	1.41	3.40	13.63	1.19	2.40
ALA11	3.96	1.65	2.72	5.69	1.07	1.29	4.04	1.61	2.61	3.38	1.97	2.98

a The standard deviations for the average magnitudes presented here are reported in Table S5.

### Effect of Xeroprotectant Concentration

All the data presented in the previous section were obtained by considering fixed xeroprotectant concentrations of 4–8 wt % in the initial water-solvated environment. Then, with the aim of investigating the effect of the initial xeroprotectant concentration on all properties studied during the previous section, we have selected PvLEA as a model system and increased its initial concentration up to 26 wt % by increasing the number of peptide models present in the simulation cells by 6 times. We have thus compared the membrane/xeroprotectant interactions and the membrane structural and mechanical properties for PvLEA in their low (5 wt %) and high (26 wt %) concentrations, with the control simulations without the presence of xeroprotectant. The density profiles depicted in Figure S20 demonstrated that the introduction of more xeroprotectants in the simulation cell does not impact the overall distribution of the membrane components, thus resulting in similar profiles as the ones presented by the lower xeroprotectant concentrations and by the control simulations. As a result, the interaction with the membranes in the water environment was not altered upon addition of the new xeroprotectant peptides. Nonetheless, in the dry state, higher xeroprotectant concentrations promote the intermolecular interactions between molecules, and due to this, the interactions of both peptides and water molecules with the membranes are weakened when compared to the low concentrations; as can be observed in the RDF plots collected in Figure S21. Then, due to the higher number of xeroprotectants present in the cell, their number of H-bonds with the membranes increases in both the wet and dry scenarios (see Table 2). Still, the number for H-bonds per xeroprotectant molecule formed with the membrane is lower than in the case of the low xeroprotectant concentrations, thus confirming that the formation of H-bonds with the membranes was already saturated when a certain xeroprotectant concentration was reached. Concerning their impact on the structural properties of the membranes, the alignment of the acyl chains in the wet environment was not influenced by the presence of new xeroprotectants, as deducted from the S_CD_ plots presented in the top part of Figure S22. Nevertheless, when moving to their dry state, the increase in xeroprotectant concentration translates to a lowering in the alignment of the acyl chains (i.e., computed S_CD_ values) for both POPC and DMPC layers (see bottom part Figure S22). Thus, increasing the xeroprotectant concentration proved to be an effective strategy for preventing transitions to highly ordered phases. As a matter of fact, moderate increases in acyl chain order may help preserve membrane structural integrity during dehydration, whereas excessive ordering could promote highly rigid gel-like phases that may impair membrane functionality. Therefore, our concentration-dependent simulations suggest the existence of a tunable regime in which xeroprotectants reduce dehydration-induced membrane collapse, while avoiding excessive overordering. Furthermore, the remaining structural (area per lipid and lateral diffusivity) and mechanical (lateral tension and area compressibility modulus) properties of the membranes did not appear to be significantly affected by the xeroprotectant concentration (see Table 2 and S3). When compared to low xeroprotectant concentrations, the lipid bilayer thickness slightly increased when moving to higher concentrations in both wet and dry environments (see Figure S18). To sum up, our simulations showed that when reaching a certain concentration of xeroprotectants, the interaction between the individual molecules and the membranes is weakened and, what is more, the ordering of membrane acyl chains starts to decrease without inducing a major impact on the overall membrane structure.

### Effects of Thermal Stress and Desiccation Performance

Up to now, we have analyzed solvated xeroprotectant/membrane interfaces working at constant room temperature and embedded in extreme hydrated environments: fully solvated and almost dry scenarios. However, as a matter of practice, xeroprotectants must preserve membrane integrity against the thermal stress induced by several heating/cooling cycles and upon different contents of water during the desiccation process. In this regard, with the aim of getting a deeper understanding of the influence of the temperature on the integrity of the membrane under the effect of the xeroprotectants, we have carried out a cooling quench process where the interfaces initially equilibrated at 350 K were gradually cooled up to 250 K, thus being able to identify the possible phase transitions that may occur in the membrane structure due to thermal stress. Note that this cooling process aims to mimic real cryopreservation protocols, where gradual cooling allows water to leave cells before ice forms, reducing the level of intracellular ice damage. However, these simulations represent simplified thermal perturbation models relevant to membrane stabilization rather than direct simulations of biological freezing processes. Moreover, in order to finely track the desiccation phenomena, we have considered different percentages of water removal: 0 (wet scenario), 10, 25, 50, 75 and 95% (dry scenario). Due to the high load of simulations required, we selected one representative xeroprotectant for each family (PvLEA, CAHS11, and vitamin E) and compared their results against the control simulations. In more detail, for these systems, we implemented the same procedure as the one employed for the room-temperature simulations including the analysis of membrane/xeroprotectant interactions and the structural characteristics of the membranes and the xeroprotectants.

Starting with the overall interface picture provided by the density profile analysis, Figure 5 contains the densities for the different interface components of the extreme cases of the quench simulations: the wet (0%) and dry (95% of water removal) solvated environments for the starting high (*T* = 350 K) and final low (*T* = 250 K) temperatures of the cooling process. Within the fully solvated scenario (left part Figure 5), the density profiles for the LEA and TDP systems are very similar to the ones of the control simulations and to those from Figure 2 left. Note that for low temperatures (*T* = 250 K) the constraint in the mobility reduces the broadening of the density distributions, thus appearing various maxima per distribution. However, for vitamin E thermal motion promotes the penetration of the α-tocopherol molecules inside both POPC and DMPC lipid bilayers, which remain inside the membrane during the entire quench process (from 350 to 250 K). As a matter of fact, the permeability calculations (Section 3.1) provided a thermodynamic rationale for the different membrane localization behaviors observed during MD simulations. The predicted high permeability and hydrophobic partitioning of vitamin E are consistent with its insertion into the acyl-chain region during MD. Conversely, the large free-energy barriers predicted for peptide xeroprotectants explain their preferential localization near membrane headgroups and dehydration-enhanced hydrogen bonding. Then, when moving to the dry scenario the domains for each interface component are less marked, leading to broader distributions with several peaks. This feature is especially notable for the control simulations where the acyl chain distribution extends to the entire normal axis, with the remaining water molecules located close to the lipid headgroups. Interestingly, due to the presence of the xeroprotectants the density profiles features remained closer with respect to the hydrated scenario, especially in the case of PvLEA and CAHS11. In the case of vitamin E due to the shrinking of the lipid lateral dimensions in the dry environment, the penetration of α-tocopherol molecules inside the membranes was lower.

**Figure 5:**
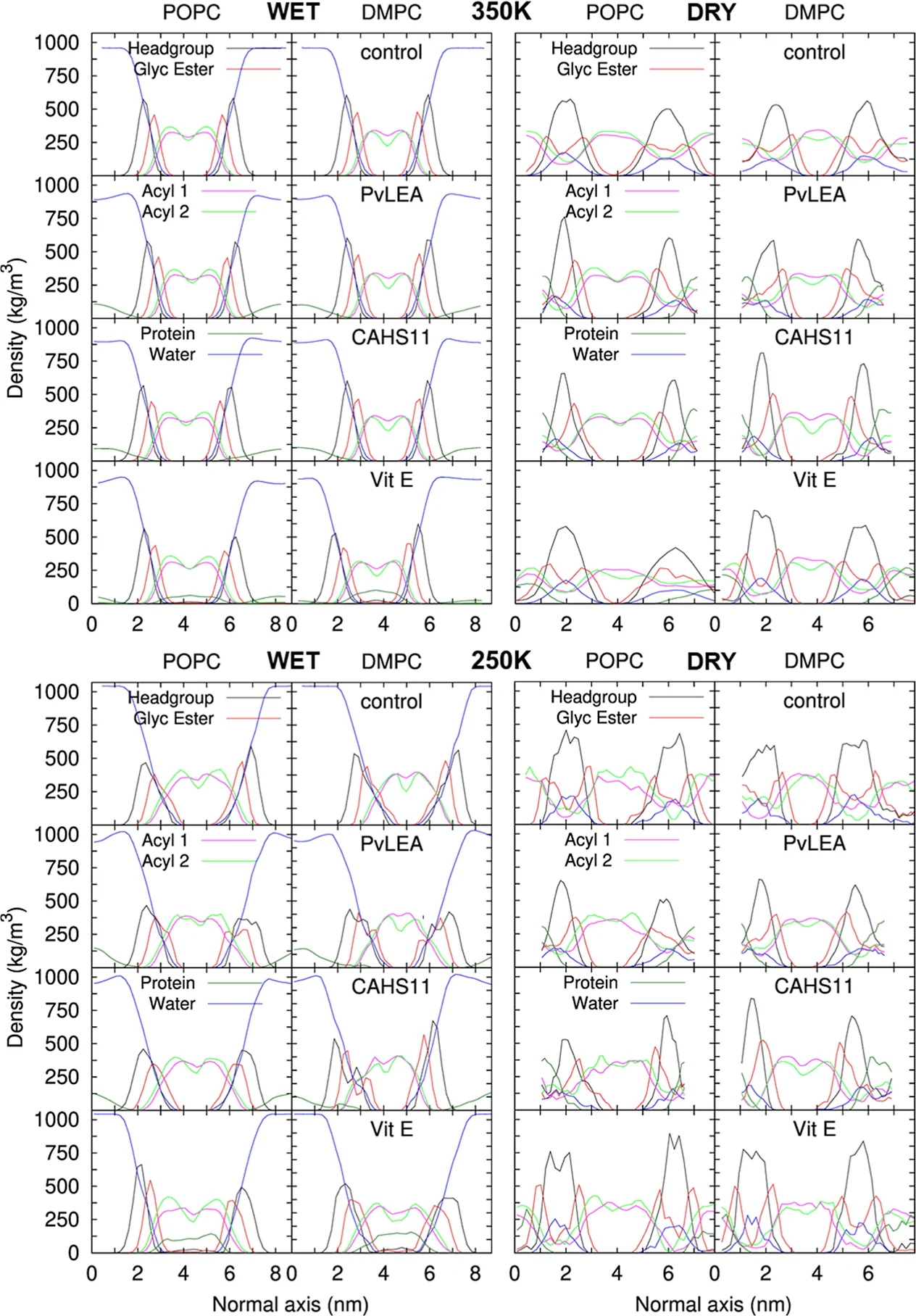
Average density profiles along the normal direction to membrane plane of the POPC and DMPC lipid bilayer (phospholipid headgroups, glycerol ester, two acyl chains) and water solvent of the membranes embedded in pure water (control) and solvated PvLEA, CAHS11 and vitamin E environments (from the top to the bottom), within their respective fully solvated (left) and dry (right) scenarios, and for the two extreme temperatures from the quench simulations: 350 K (top) and 250 K (bottom panels).

Moving now to the xeroprotectant/membrane interactions, Figures S23 and S24 shows the RDF plots of the water and xeroprotectant molecules with respect to the lipid bilayer atoms in both wet and dry interfaces, for 350 and 250 K, respectively. The RDF plots in the hydrated environments for the control simulation, PvLEA and CAHS11 at 350 K remained very similar to the ones plotted in Figure 4 left. Note that in the case of vitamin E, the penetration of α-tocopherol molecules inside the membrane significantly enhanced the interactions with the lipid bilayer groups. During the cooling down process at 250 K, with the exception of vitamin E, the interactions between xeroprotectant and membranes were lower. On the other hand, dehydration led to a similar enhancement of the water/membrane and xeroprotectant/membrane interactions as the one observed in Figure 2 right. Once more, vitamin E represents the only exception for that, displaying similar interaction strength in both wet and dry scenarios. In this case, the nature of the xeroprotectant/membrane interactions remained unaltered during the cooling steps (350 K vs 250 K). In addition, in order to get a more qualitative and detailed analysis, we computed the number of H-bonds formed between the xeroprotectant molecular systems and the membrane lipid groups for all cooling steps, and for all considered dehydration ratios, as they are represented in Figure S25. For dehydrated ratios up to 50% of water removal (0, 10, 25 and 50%), the number of H-bonds remained quite similar and decreased nearly linearly with the temperature. Strikingly, for high ratios of water removal (75 and 95%), the number of H-bonds formed with the membrane is significantly boosted (especially for 95%) and remained almost independent of the temperature step. Despite their penetration inside the membrane, the number of H-bonds formed by the vitamin E xeroprotectants and the lipid groups was found 1 order of magnitude lower with respect to the peptide systems (PvLEA and CAHS11). Indeed, the highest number of H-bonds was found in the interactions between CAHS11 and DMPC bilayers for the dry scenario. Finally, with the aim of discerning which amino acid groups presented the strongest interactions with the membrane, we computed the number of H-bonds formed by the distinct residues of PvLEA and CAHS11 in the dry scenario at both 250 and 350 K (see Figure S26). The resulting H-bond plots were identical to the ones shown in Figure S14, thus confirming that lysine amino acid groups presented the more marked interaction with the membranes independently of the simulation temperature.

Regarding the structural properties of the membranes, Figure 6 displays the deuterium order parameters S_CD_ of the POPC and DMPC acyl chains for the extreme dehydrated cases (0 and 95%) and for the initial and final temperatures of the cooling simulation, 350 and 250 K, respectively. In the wet environment at high temperatures (350 K), the *S*_CD_ plots of the solvated xeroprotectant interfaces were identical to the one from the control simulation, in line with the trends observed in Figure 4 left. However, when moving to low temperatures (250 K), the acyl chains undergo to more ordered (gel) phase, thus resulting in higher alignment and *S*_CD_ values. However, at 250 K the presence of xeroprotectant produces a marked effect in the acyl chain alignment depending on the lipid bilayer considered: whereas for POPC layers the *S*_CD_ values of the xeroprotectants were slightly similar to the ones from the control simulation (even lower in the case of CAHS11 and vitamin E); for DMPC the presence of xeroprotectants translated to more aligned acyl chains, only with the exception of vitamin E where this effect was minor. Surprisingly, due to loss of structural integrity derived from the combination of thermal and desiccation stress, acyl chains in the dry scenario were consistently lower with respect to the hydrated interfaces, which differs from the results of Figure 4 and previous observables from the literature.^37^ As it was found in Figure 4 right, the introduction of xeroprotectants led to an increase in the *S*_CD_ values when compared to the control simulations. Once more, this increase in the chain alignment was found more pronounced in DMPC when compared to POPC and, contrary to the wet environment, CAHS11 presented the largest deviations with respect to the control simulations.

**Figure 6:**
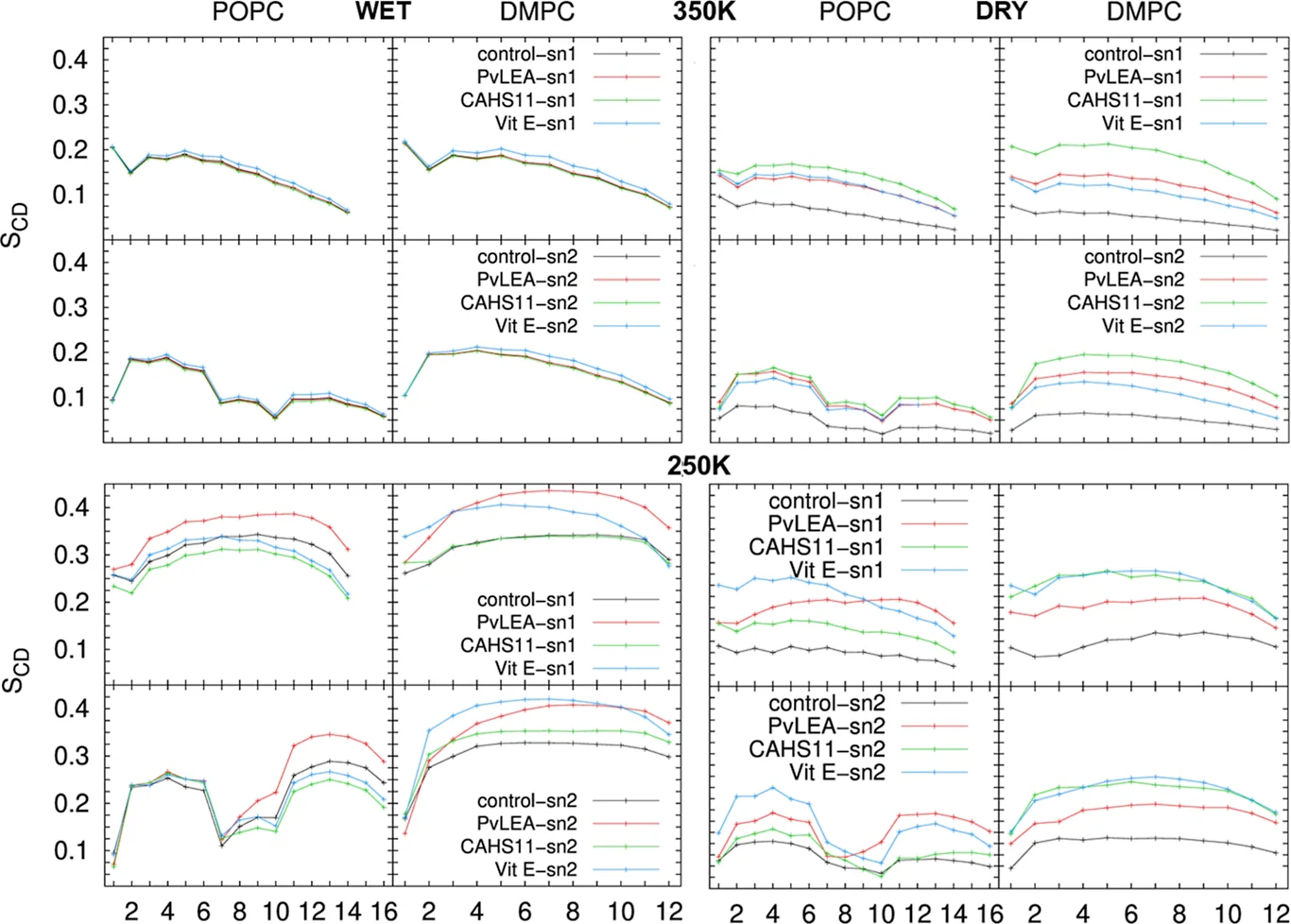
Deuterium order parameters *S*_CD_ of the carbon atoms belonging to two lipid acyl chains (sn1 and sn2) from the POPC and DMPC lipid bilayers integrating the membrane cell models embedded in pure water (control) and solvated xeroprotectant (PvLEA, CAHS11 and vitamin E) environments, within their respective fully solvated (wat, right part) and desiccated (dry, right part) scenarios; and for the two extreme temperatures from the quench simulations: 350 K (top) and 250 K (bottom panels).

Concerning the rest of structural properties, Figures S27 and S28 display the evolution of the computed area per lipid *A*_*l*_ and lateral diffusivity *D*_*l*_, respectively, for the interfaces with distinct water contents as a function of the temperature of the cooling step. Note that areas from the interfaces with high dehydration ratios (75% and 95%) were not included here since the instability of the lipid bilayers hindered the estimation of reliable lipid areas. Due to the ordering of the acyl chains triggered by the cooling process the lipid bilayers shrink when decreasing the temperature (see Figure S27), being in agreement with previous works.^37^ In the case of POPC bilayers, all interfaces experimented an almost linear decrease of *A*_*l*_ with the temperature of about 15 Å^2^ from 350 to 250 K. For these lipid layers the influence of the xeroprotectant, or the dehydration ratio were minor. Nevertheless, for DMPC layers the drop of *A*_*l*_ with the temperature (about 15–20 Å^2^ from 350 to 250 K) started to saturate at 290 K and more importantly, the impact of the xeroprotectants and the water removal were more pronounced. In some cases, such as PvLEA or vitamin E, the presence of the xeroprotectants led to an increase of the *A*_*l*_ with respect to the values showed by the control simulation. Furthermore, as a general trend the increase of the dehydration percentages translated to a decrease of *A*_*l*_, being the interfaces with 50% of dehydration ratio the ones exhibiting the lower *A*_*l*_ in almost all cases, thus going in line with the outcomes from Table 2. Regarding the membrane mobility, both the lowering of the temperature and the water content slowed down the lipid bilayer dynamics, as previously observed in former works.^37^ However, the drop in the diffusivities starts to be especially prominent at dehydration ratios of 95%, which shows *D*_*l*_ values about two or 3 orders of magnitude lower than the rest of water contents and furthermore, for these dry interfaces the decrease of *D*_*l*_ during the cooling process was not so pronounced. Then, the dry interfaces exhibiting the slowest dynamics were those where PvLEA and CAHS11 were present.

Afterward, we evaluated the evolution of the thickness of the bilayer with the temperature and water removal by plotting their corresponding section 2-D maps of the extreme conditions: fully solvated and dry scenarios at the final snapshots from the simulations carried out at 350 K (see Figure S29) and 250 K (Figure S30). For the fully solvated interfaces, due to the alignment and ordering increase of the acyl chains during the cooling process, the thickness of the bilayers is enhanced when moving from 350 K (thickness values of 3–5 nm for POPC and 2.5–4.5 nm for DMPC) to 250 K (3.5–6 nm for POPC and 3–5.5 nm for DMPC), being in line with the reference theoretical study which observed an increase of about 1 Å from 50 to 30 °C.^37^ Note that the homogeneity in the bilayer thickness is overall decreasing due to the cooling process as well. In this wet scenario, the presence of the introduction of the xeroprotectants induced an increase in the bilayer thickness when compared to the control simulations, which agrees with the xeroprotectant induced enhancement of the acyl chain alignment (i.e., *S*_CD_ values) observed in Figures 2 and 6. More in detail, interfaces containing vitamin E exhibited the largest thickness, which may be related with the penetration of these molecules into the lipid bilayers. Turning to the dry scenario, at 350 K the impact of the dissection process was similar to the one observed previously at room temperature (Figures S18 and S19), showing an increase of both height and inhomogeneity (especially for DMPC) of the bilayer thickness. In this case, the presence of xeroprotectants was mitigating the drop in the thickness homogeneity derived from the water removal. Remarkably, for 250 K the trends in the 2-D maps were different due the loss of integrity during the cooling process in the dry environment, thus resulting in thin and high heterogeneous lipid bilayers, being both effects significantly pronounced for DMPC. Indeed, the 2-D maps of the control interfaces of DMPC in the dry scenario at 250 K were dominated rather thin lipid domains (1–2.5 nm), which were partially disappearing under the action of the xeroprotectants, especially for PvLEA whose lipid domain heights were in the order of 2.5–5 nm. In summary, despite the presence of xeroprotectants may lead to ordered (gel) elongated acyl chains distributions, they prevent the membrane cell against the inhomogeneity and the structure integrity induced by the combination of thermal and desiccation stress.

With the aim of studying the role of thermal stress in the structural dynamics of the xeroprotectant peptides (PvLEA and CAHS11) at distinct levels of hydration, we have analyzed the changes in the peptide geometrical descriptors (RMSD, *R*_g_, and H-bonds formed within the peptide structure) due to the thermal motion induced during the cooling cycles; which are represented in Figures S31–S33. For all studied interfaces, the cooling quenching produced a slowing of the peptide mobility (drop in rmsd values), and as was the case of the lipid chains, it induced an elongation of the peptide backbone which translated to a loss of its compactness (increase in the gyration ratios *R*_g_) and hampered the formation of internal H-bonds. These trends are peculiarly remarkable for lower dehydration percentages (up to 50%), whereas in the particular case of interfaces with 95% dehydration ratios, all peptide geometrical descriptors were almost independent of the temperature step. As a matter of fact, all interfaces displayed comparable peptide geometries at low dehydration percentages (<50%), which were then completely modified when moving to highly desiccated environments, which are characterized by high compact and rigid peptide structures (low rmsd and *R*_g_ with high number of internal H-bonds), as was also observed at room temperature (see Table 4). In particular, the xeroprotectant exhibiting the more compact structure in the dry scenario corresponded to CAHS11 in contact with the POPC bilipid layer.

In the final step, we focused on the influence of the xeroprotectant thermal motion and the desiccation environment. in the organization of supramolecular aggregated structures. To this end, we plotted the number and size of the clusters formed during the cooling process at different temperatures and dehydration percentages (see Figures S34–S36). As a general rule, the decrease in the xeroprotectant mobilities experimented during the cooling process hindered the formation of novel clusters and enabled the development of larger and more ordered supramolecular peptide structures (i.e., increase in the cluster size). Again, these effects were more evident for low ratios of water removal, while in the case of 95% dehydration percentages, both the number and size of clusters remained constant during the cooling cycles. As previously reported, when moving to the dry scenario (95% water removal), the intermolecular interactions between xeroprotectants are lowered in favor of the interactions with the membrane, thus reducing the size (and increasing the number) of the clusters found (see Table 5). In this case, the dry environment CAHS11 embedded in DMPC layers exhibited the smallest aggregated structures. For the rest of dehydration ratios, this drop in the cluster number and size was not that significant. On the same footing, the effect of both thermal motion and water removal in the number and size of the clusters formed by vitamin E molecules was much less marked. To conclude, our findings evidenced the capability of xeroprotectants to maintain the membrane integrity in a wide range of temperatures (±50 °C from *T* = 300 K) even in shortly packed lipid bilayers as DMPC, thus being able to operate at room temperature under extreme conditions and avoiding highly costly cryopreservation processes at extremely low temperatures.

It should be noted that the LEA and TDP systems investigated here were modeled as short-motif-derived peptides rather than full-length proteins. Native LEA and TDP proteins typically exceed 10 kDa and may exhibit multivalent and cooperative interactions that are not captured in minimal peptide models. Therefore, while the simulations isolate fundamental interaction mechanisms, they may not fully represent higher order assembly or crowding effects present in native systems.

## Conclusions

In this computational study we have analyzed the different factors behind the performance of a series of natural peptides (such as LEA or TDPs) and molecular antioxidants (i.e., vitamin E) to mimic the process of anhydrobiosis in mammalian cells exposed to thermal stress and/or desiccation phenomena. The outcomes provided by this multiscale modeling platform allowed us to set a series of design principles: (i) mammalian cell stabilization in the presence of vitamin E is achieved via molecular partitioning of α-tocopherol into the hydrophobic region of POPC/DMPC bilayers, which was found thermodynamically favorable, following an opposite trend as LEA and TDPs, whose polar groups hindered their diffusion into the membrane, thus assuring the membrane integrity; (ii) for LEA/TDP xeroprotectants, the formation of H-bonds between the peptide amino groups (i.e., lysine residues) with the membrane stabilizes mammalian cells in their dry state, as evidenced by the mitigation of some of the structural modifications triggered by the desiccation process, such as the shrinking or the loss in homogeneity of the membrane layers; (iii) the enhancement of the ordering/gel in the lipid chains associated with the presence of the xeroprotectants can be significantly reduced by increasing the initial concentration of xeroprotectants which, on the other hand, should be carefully monitored to avoid overpacking and maintain function; (iv) avoiding intermolecular aggregation and promoting xeroprotectant stabilization and compaction can be used to enhance its interaction with the membrane in its dry state; (v) the loss of integrality observed in less packed bilayers (i.e., DMPC) under the combined action of desiccation and thermal stress is hindered by the presence of the xeroprotectants (mainly LEA polypeptides), whereas for highly dry environments (water removal >50%) the effect of the temperature was found almost negligible.

The peptide models employed here represent minimal functional motifs of LEA and TDP proteins and do not capture the full structural complexity or multivalency of the native proteins. Consequently, the quantitative magnitudes of stabilization effects may differ for full-length proteins, although the underlying interaction principles identified in this study are expected to remain relevant. In this Viewpoint, the mechanistic molecular-level insights provided here require future validation in more realistic models (i.e., specific membrane compositions, full proteins) and experimental cellular systems. In the same vein, despite the present study not directly simulating permeability leakage, rupture, or viability, where future experimental validation will be required to establish quantitative links between these molecular descriptors and biological preservation outcomes, it provides valuable mechanistic insight into how xeroprotectants modulate membrane structural responses during dehydration and thermal stress.

Overall, our theoretical models provide a detailed picture of the mechanisms underlying the shielding action of the state-of-the-art xeroprotectant agents currently employed in the field of cryopreservation. Then the application of the design rules for the xeroprotectants’ chemical structure mentioned above in the synthesis of artificial functional biosystems opens the door for a new generation of low-cost, environmentally friendly, and efficient xeroprotectant agents operating at room temperature. For instance, building artificial peptides with a high content of lysine amino acids, or further exploring other positively charged terminated amino acids with enhanced interaction with negatively charged phospholipids, can further boost mammalian cell resilience, thus pushing cell preservation technologies based on anhydrobiosis strategies, to go beyond the preclinical stage.

## Supplementary Material



## Notes

Structural characteristics of xeroprotectant models; standard deviations of computed H-bonds; mechanical properties of membrane interfaces; structural and aggregation standard deviations of peptides and xeroprotectants; COSMOPerm probability distributions, free energy profiles, and diffusivities; simulation snapshots; average density profiles; radial distribution functions; residue-based H-bond distributions; lipid acyl chain order parameters; 2D membrane thickness maps; H-bonds formed as a function of temperature; area per lipid and lateral diffusivity during cooling; and structural/aggregation metrics during cooling quenching processes. COSMO files containing the coordinates and electronic properties of the xeroprotectant models employed in this work can be found in the following GitHub repository (https://github.com/vdiezcabanes/COSMO-files-xeroprotectant-models-) (PDF)
