# Supplementary material for: Screening of Xeroprotectants for Efficient Mammalian Cell Structural Preservation in Dry Conditions
Source: ACS Omega. 2026 Aug 17;11(34):51254–71. doi: 10.1021/acsomega.6c04138 (PMC13625561; doi:10.1021/acsomega.6c04138)
Supplement: Supplementary file 1 [file ao6c04138_si_001.pdf]

Supplementary Information

Screening of Xeroprotectants for Efficient Mammalian Cell Structural Preservation in Dry Conditions

Valentin Diez-Cabanes[1,2], Takahiro Kikawada[3,4], Pasqualino Loi[5], Santiago Aparicio[1,2*]*

[1] International Research Center in Critical Raw Materials for Advanced Industrial Technologies (ICCRAM), University of Burgos, 09001, Burgos, Spain

[2] Department of Chemistry, University of Burgos, 09001, Burgos, Spain

[3] Department of Integrated Biosciences, Graduate School of Frontier Science, The University of Tokyo, Kashiwa, Chiba 277-8562, Japan

[4] Division of Biomaterial Sciences, Institute of Agrobiological Sciences, National Agriculture and Food Research Organization, Tsukuba, Ibaraki 305-8634, Japan

[5] Department of Veterinary Medicine, University of Teramo, Teramo 64100, Italy

Table S1. Structural characteristics of the xeroprotectant models employed in this work, as extracted from existing literature.

Family	Name	Sequence	Ref
LEA	PvLEA	AKDGTKEKAGE	26,27
	PILEA	AADGAKEKAGE	26,27
	NeLEA	AKDGAKEKAGE	26,27
	scrambled	AKEKEGTDKAGGAKDTGEKEKA	28
TDPs	SAHS	KYTEDGDKLVA	29
	CAHS11	AEKIRKELEKQ	29
	CAHS22	AEKIRKELEKQHARDVEFRKSL	29
Vitamin E	α-tocopherol	-	18,31
Alanine	ALA11	AAAAAAAAAAA	-

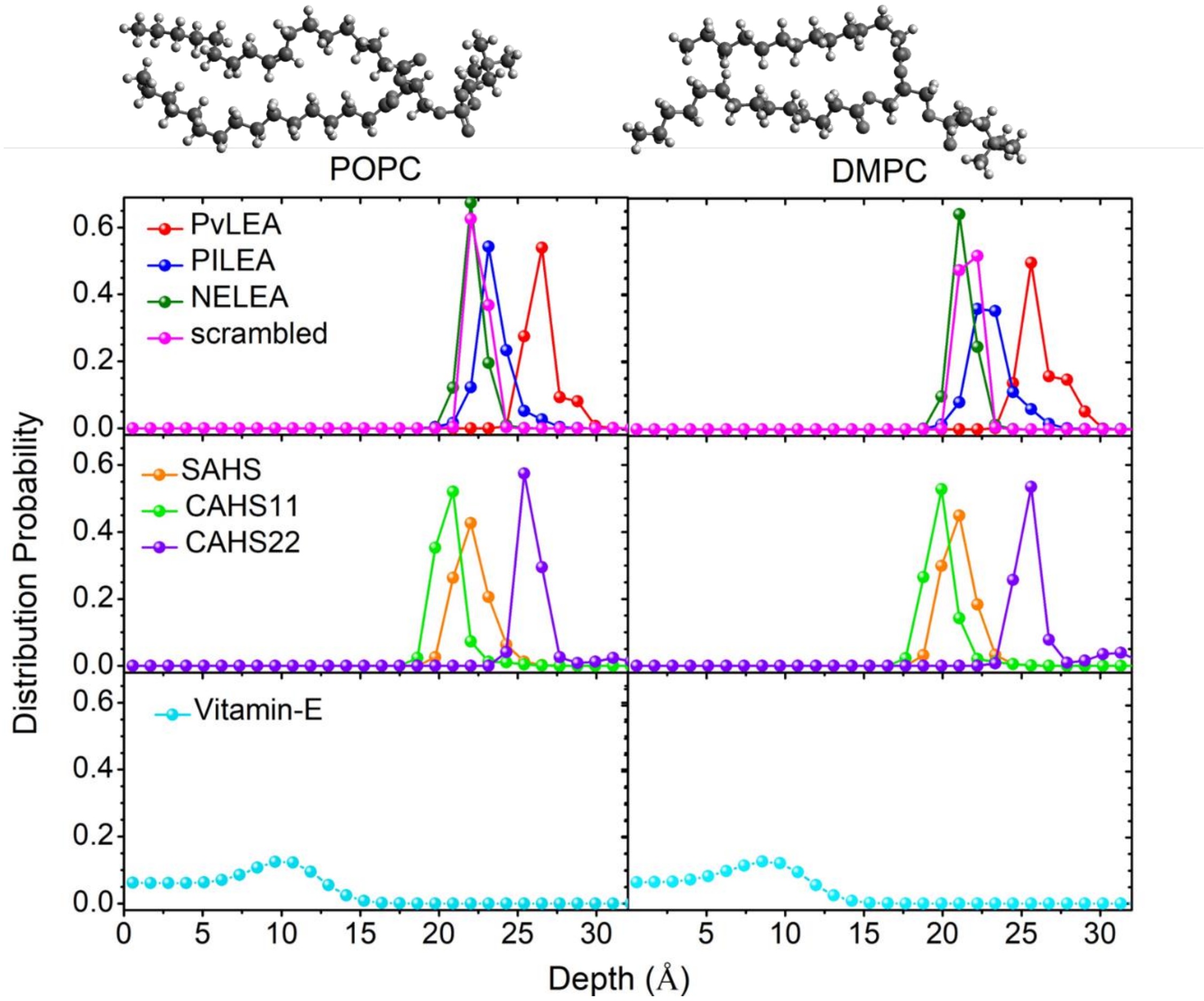

Figure S1. Distribution probability profile of the xeroprotectant models of LEA proteins, TDPs and vitamin E (from the top to the bottom panels) as a function on the depth penetration through the POPC (left) and DMPC (right part) lipid layer (from the acyl chains to the headgroups), as estimated by employing the thermodynamic COSMOPerm analysis at T=300K.

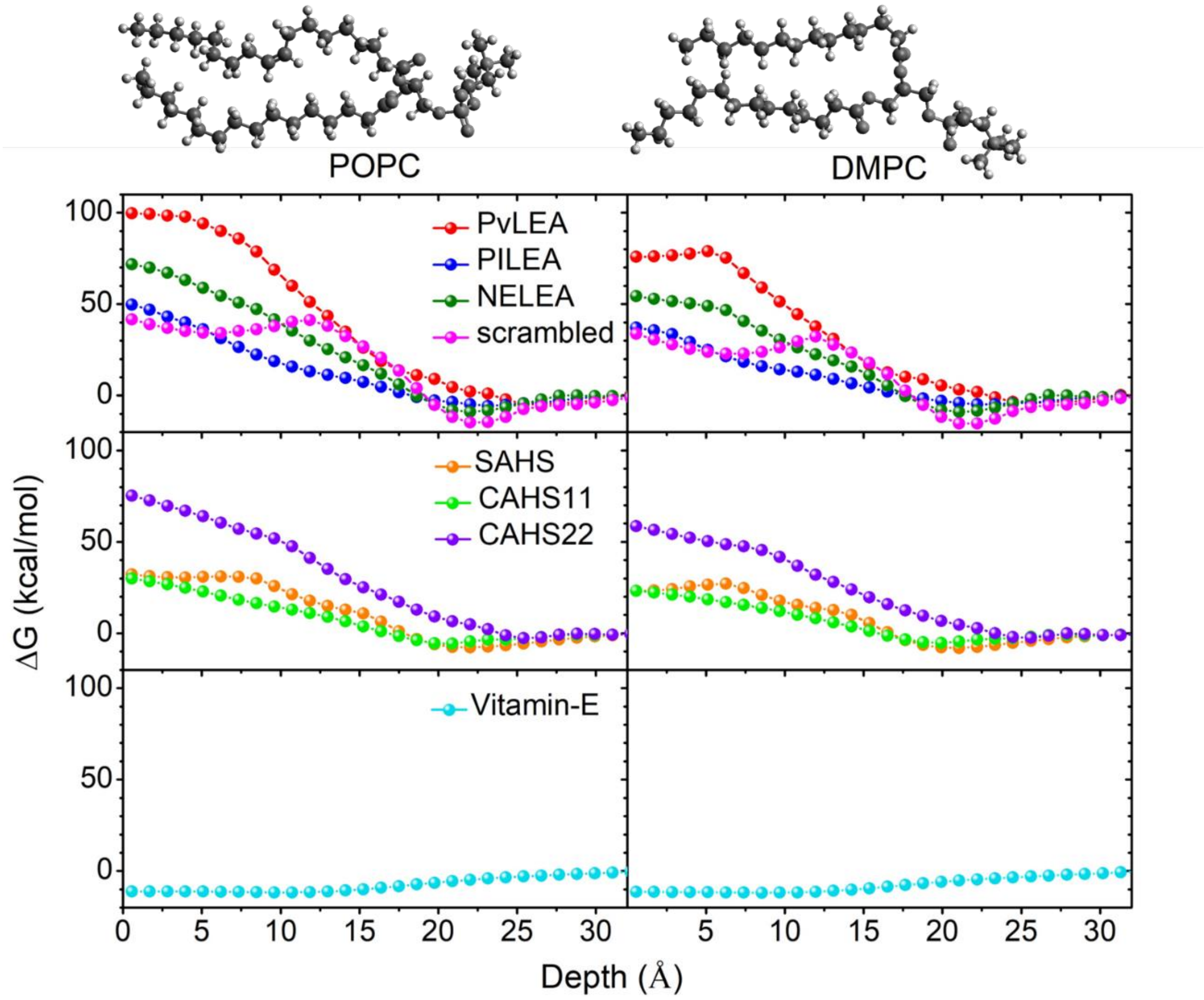

Figure S2. Free energy profile of the xeroprotectant models of LEA proteins, TDPs and vitamin E (from the top to the bottom panels) as a function on the depth penetration through the POPC (left) and DMPC (right part) lipid layer (from the acyl chains to the headgroups), as estimated by employing the thermodynamic COSMOPerm analysis at T=300K.

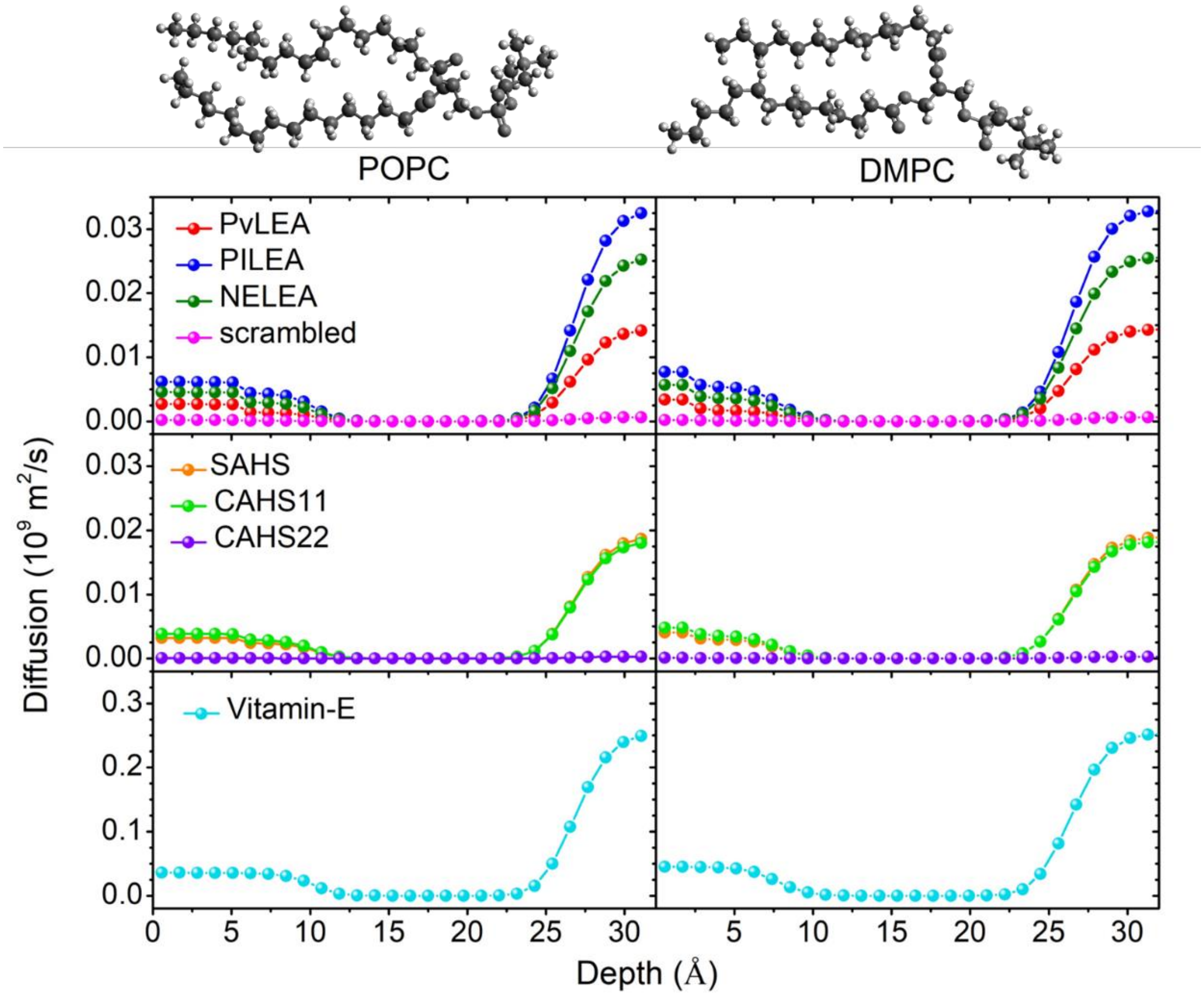

Figure S3. Diffusivity profile of the xeroprotectant models of LEA proteins, TDPs and vitamin E (from the top to the bottom panels) as a function on the depth penetration through the POPC (left) and DMPC (right part) lipid layer (from the acyl chains to the headgroups), as estimated by employing the thermodynamic COSMOPerm analysis at T=300K.

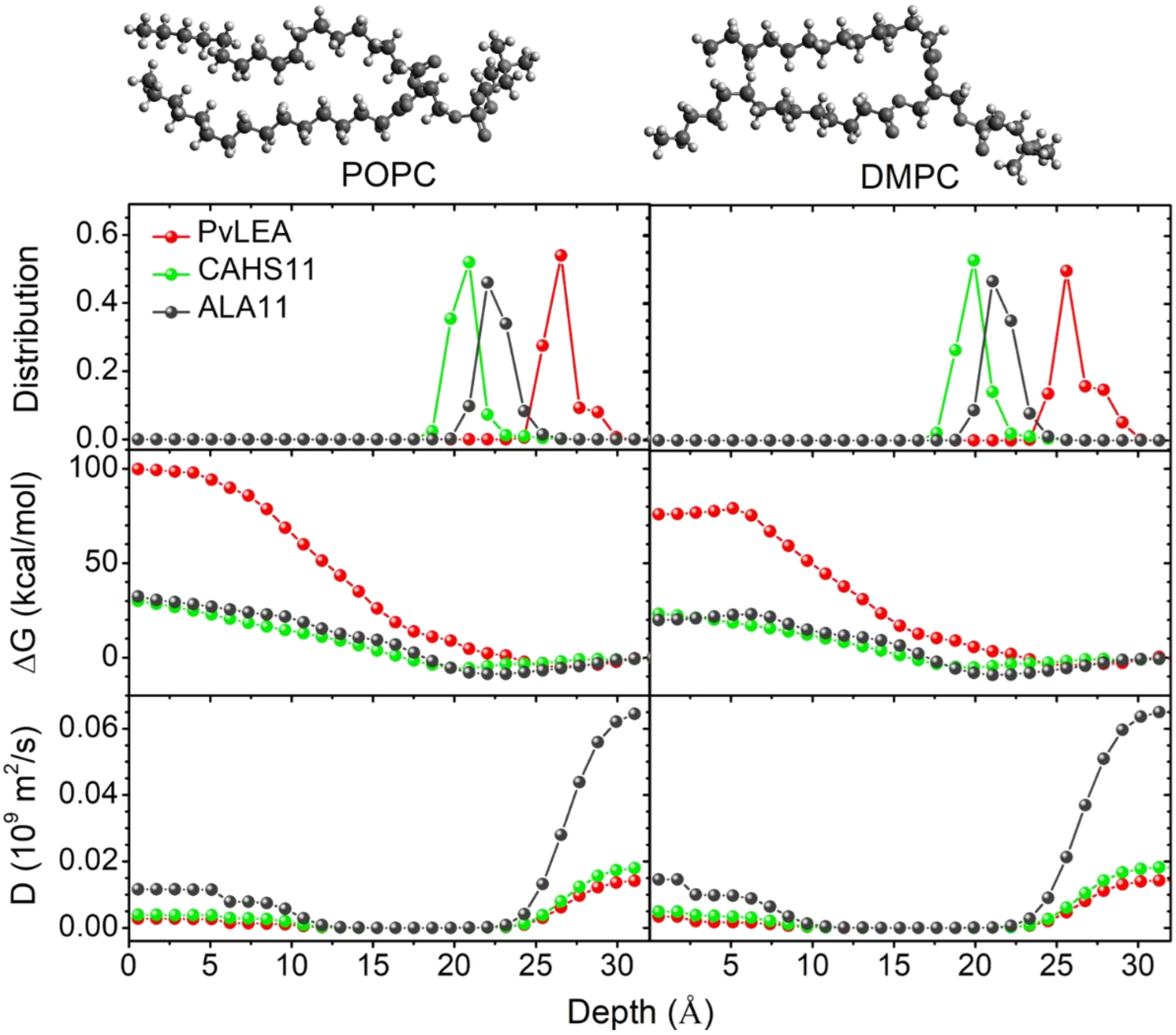

Figure S4. Probability distribution (top), free energy (center) and diffusivity (bottom) profiles of the PvLEA, CAHS11 and ALA11 protein models as a function on the depth penetration through the POPC (left) and DMPC (right part) lipid layer (from the acyl chains to the headgroups), as estimated by employing the thermodynamic COSMOPerm analysis at T=300K.

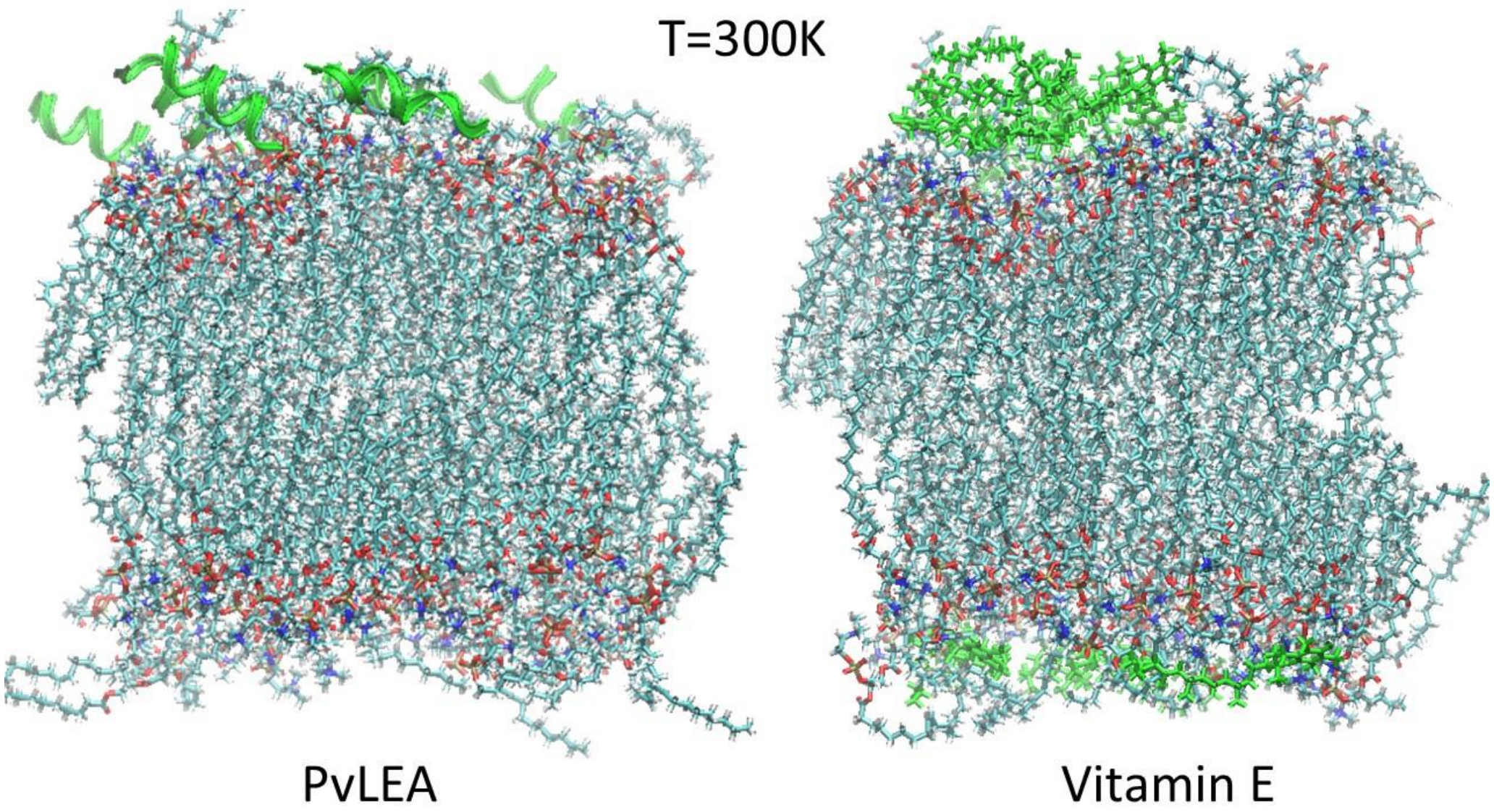

Figure S5. Lateral view of the final snapshot of the simulations performed at room temperature (T=300K) for the POPC lipid bilayers embedded in PvLEA (left) and vitamin E (right, green colors) in their respective dry environment.

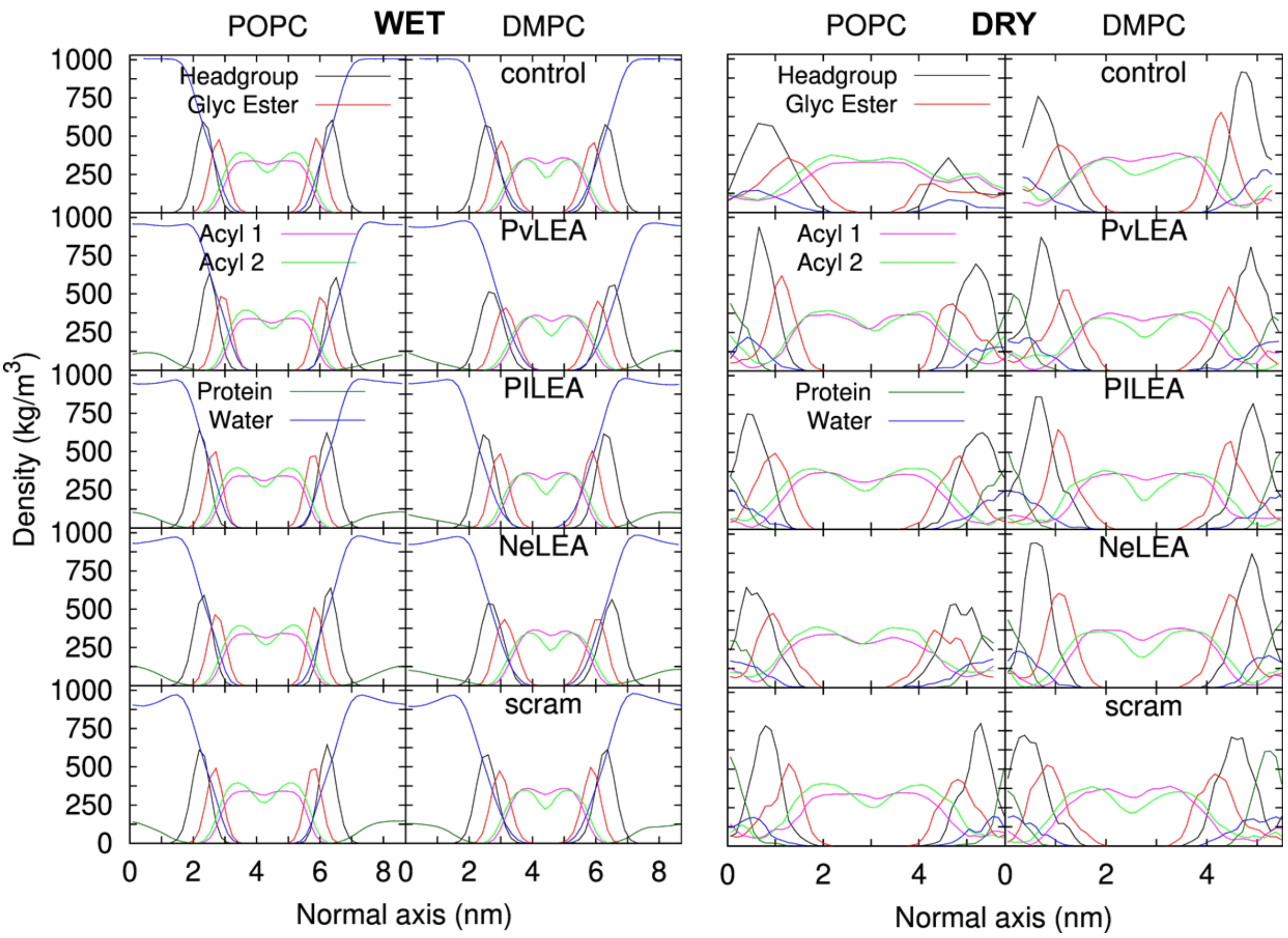

Figure S6. Average density profiles along the normal direction to membrane plane of the POPC and DMPC bilipid layer (phospholipid headgroups, glycerol ester, two acyl chains) and water solvent of the membranes embedded in pure water (control) and LEA protein solvated environments, within their respective full solvated (left) and dry (right) scenarios. All simulations were performed at room temperature (T=300K).

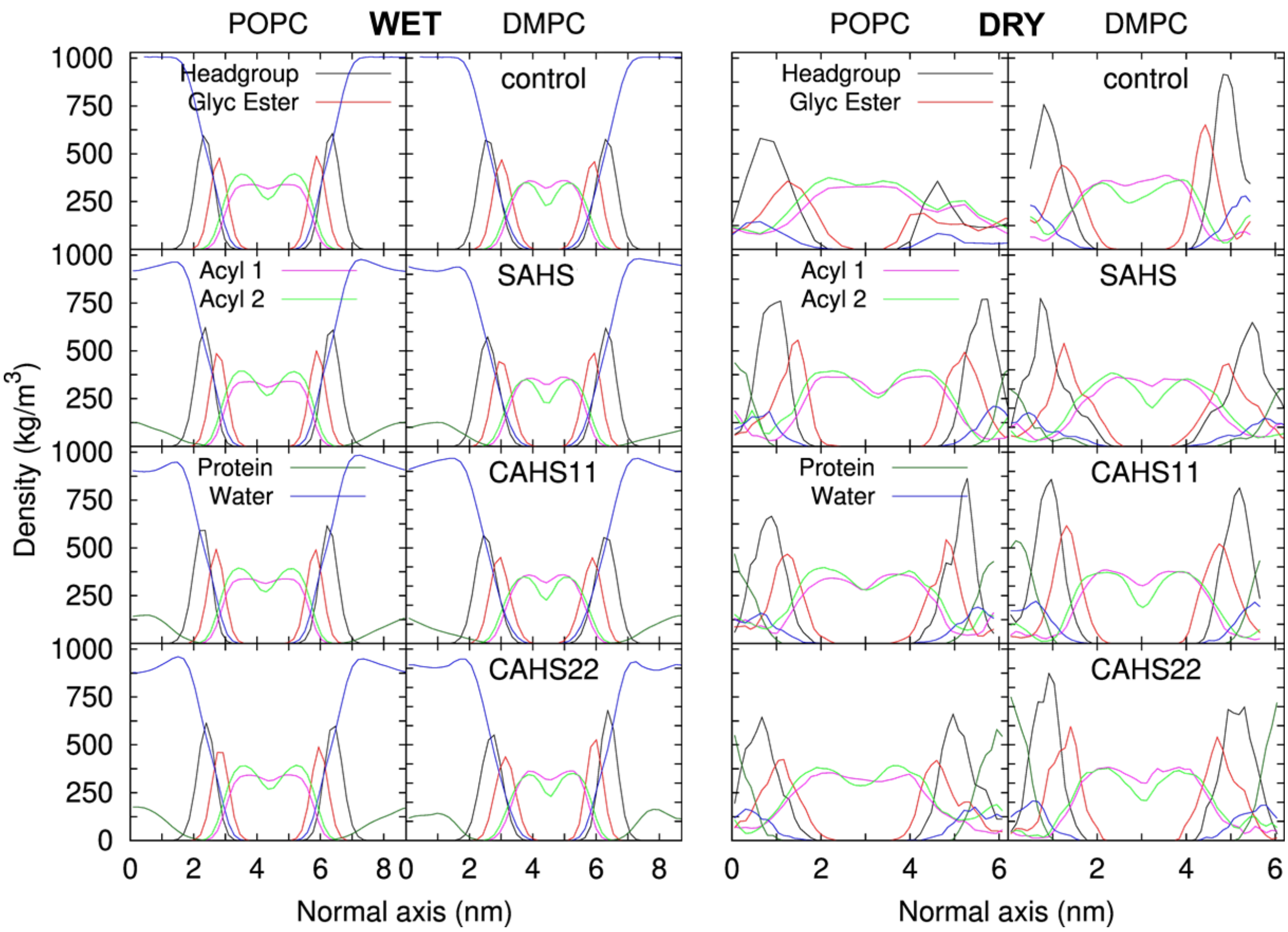

Figure S7. Average density profiles along the normal direction to membrane plane of the POPC and DMPC bilipid layer (phospholipid headgroups, glycerol ester, two acyl chains) and water solvent of the membranes embedded in pure water (control) and TDP solvated environments, within their respective full solvated (left) and dry (right) scenarios. All simulations were performed at room temperature (T=300K).

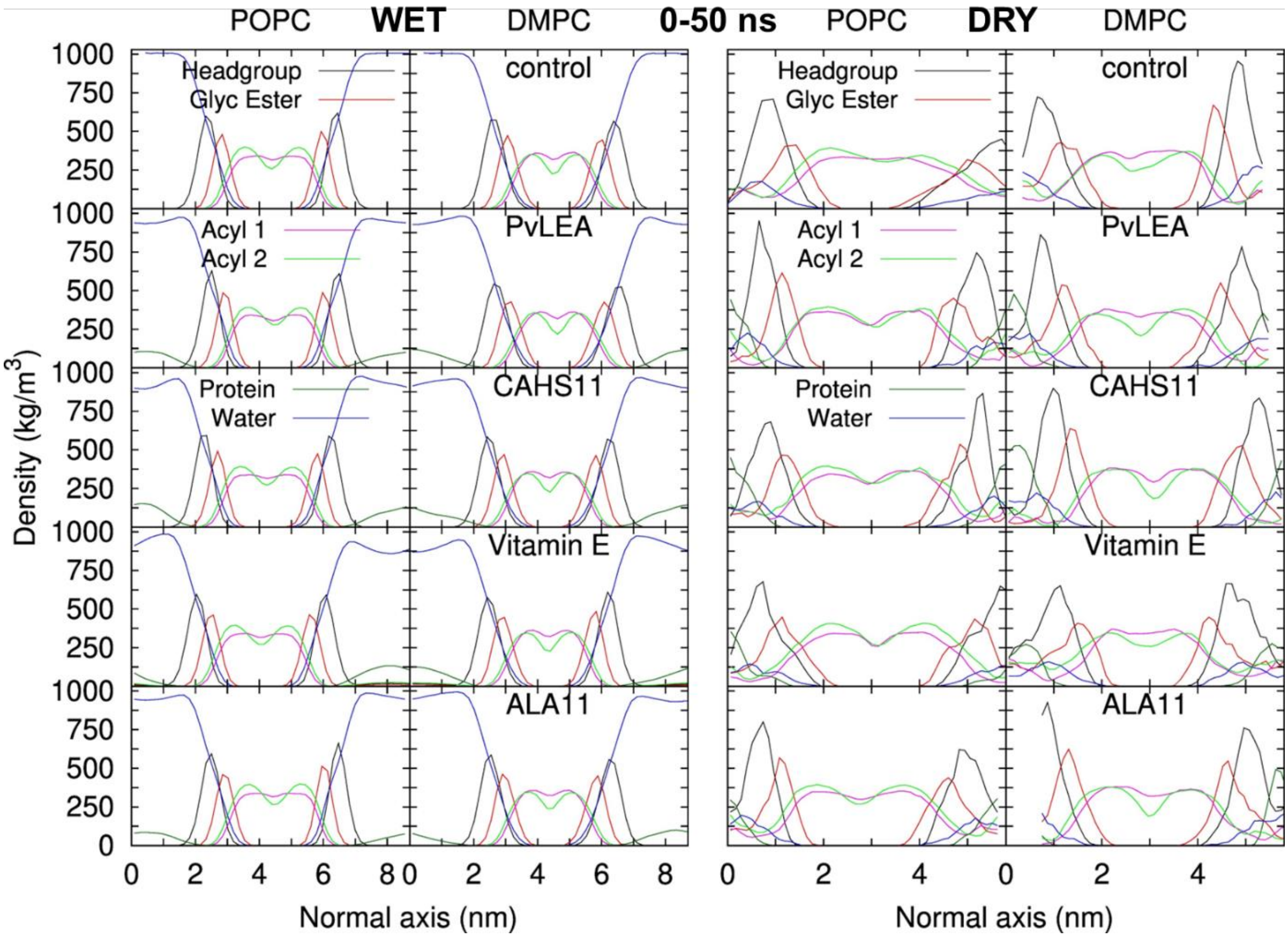

Figure S8. Average density profiles along the normal direction to membrane plane of the POPC and DMPC lipid bilayer (phospholipid headgroups, glycerol ester, two acyl chains) and water solvent of the membranes embedded in pure water (control) and solvated representative xeroprpotectants (LEA peptides, TDPs, vitamin E and apolar peptide) environments, within their respective full solvated (left) and dry (right) scenarios, as considering the first halve (0-50 ns) of the MD trajectory. All simulations were performed at room temperature (T=300K).

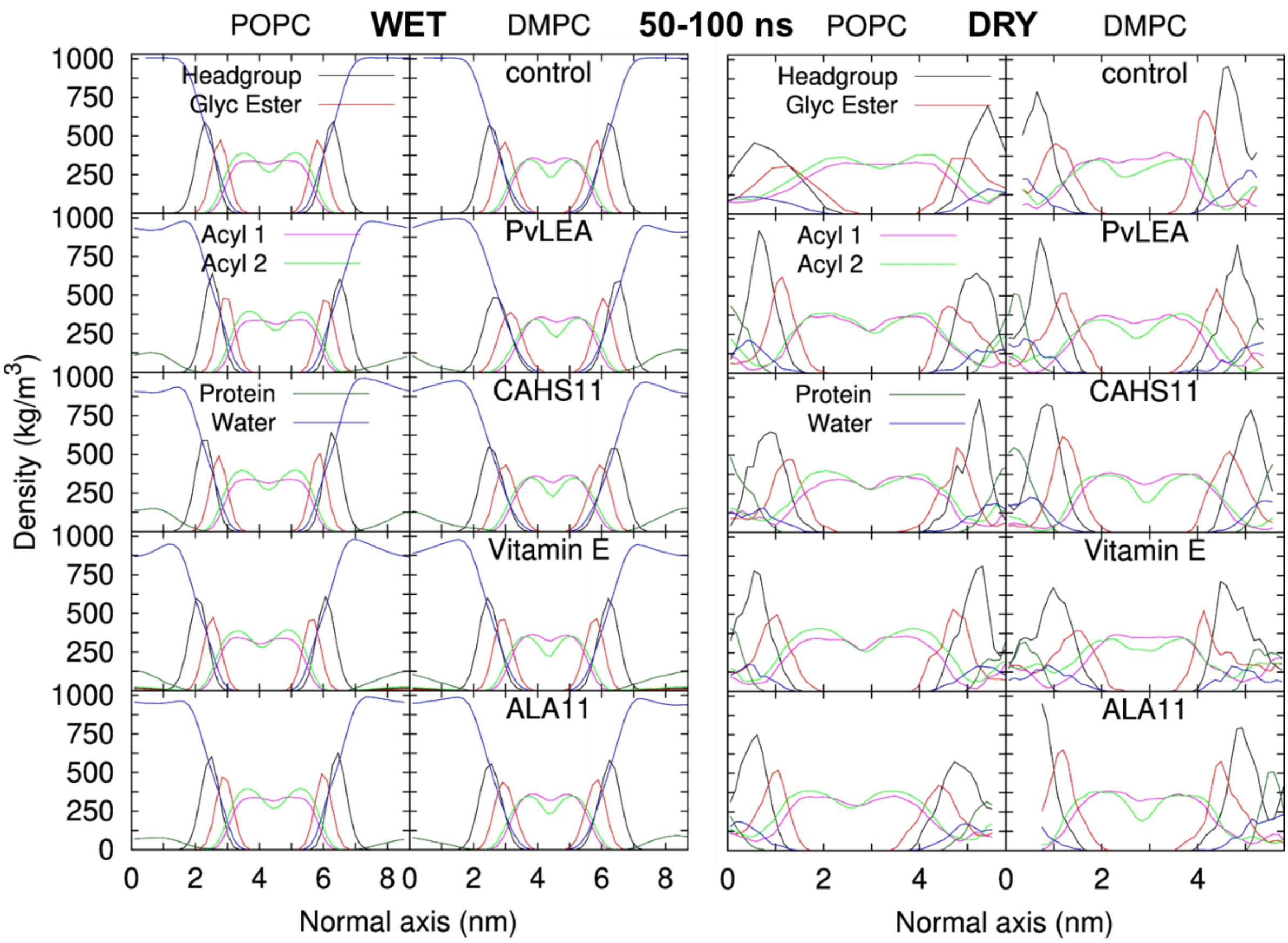

Figure S9. Average density profiles along the normal direction to membrane plane of the POPC and DMPC lipid bilayer (phospholipid headgroups, glycerol ester, two acyl chains) and water solvent of the membranes embedded in pure water (control) and solvated representative xeroprpotectants (LEA peptides, TDPs, vitamin E and apolar peptide) environments, within their respective full solvated (left) and dry (right) scenarios, as considering the second halve (50-100 ns) of the MD trajectory. All simulations were performed at room temperature (T=300K).

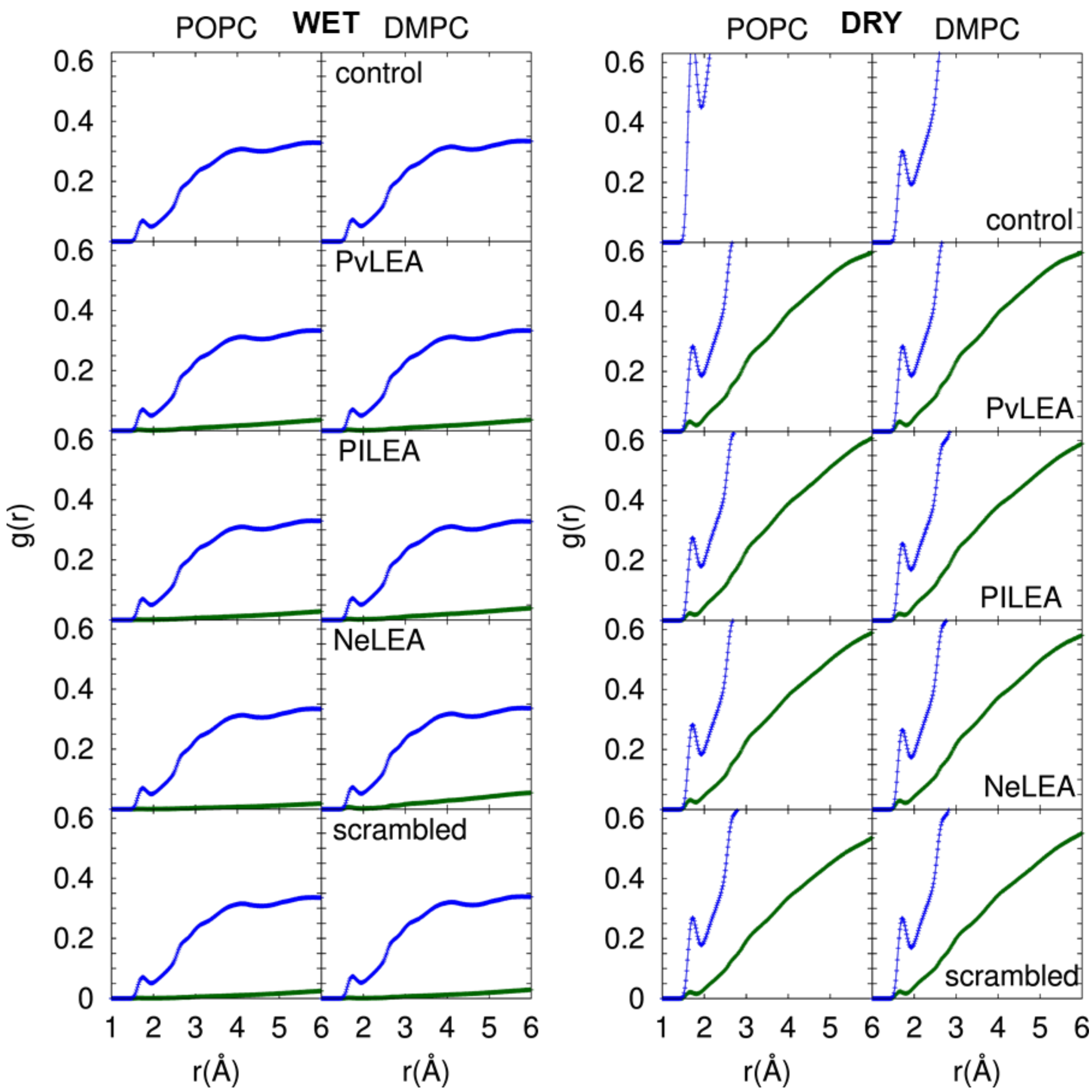

Figure S10. Radial distribution function (RDF) plots between the water solvent molecules (blue) and the LEA proteins (green), with respect to the atoms from the POPC and DMPC bilipid layers for the membranes embedded in pure water (control) and solvated LEA protein environments, within their respective full solvated (left) and dry (right) scenarios. All simulations were performed at room temperature (T=300K). Only distances from the membrane atoms lower than 6Å were plotted here.

Table S2. Standard deviations for the computed number of H-bonds formed between the xeroprotectant ($(H_b)_{mol}$), the water solvent molecules ($(H_b)_{wat}$); within the full solvated (wet) and desiccated (dry) scenarios simulated at T=300K, as reported in Table 2 of the main text.

		POPC				DMPC			
		wet		dry		wet		dry	
Xero	wt%	$(H_b)_{mol}$	$(H_b)_{wat}$	$(H_b)_{mol}$	$(H_b)_{wat}$	$(H_b)_{mol}$	$(H_b)_{wat}$	$(H_b)_{mol}$	$(H_b)_{wat}$
PvLEA	5.5	1.5	17.0	1.8	13.0	1.7	17.9	2.0	11.1
PvLEA	25.9	1.7	17.4	3.0	8.1	2.5	20.6	3.0	8.6
PlLEA	5.1	1.1	20.2	2.1	8.2	1.4	17.9	1.9	6.1
NeLEA	5.4	0.9	19.3	4.3	7.0	1.5	17.9	2.7	7.0
scrambled	7.2	1.1	17.7	1.9	9.4	1.7	17.5	1.9	8.3
SAHS	6.0	2.5	20.0	2.0	8.5	1.3	19.7	4.8	10.3
CAHS11	6.6	1.5	18.3	4.1	8.0	1.8	26.9	5.2	9.5
CAHS22	8.5	2.6	18.7	2.2	12.1	1.9	18.1	3.9	8.6
Vitamin E	5.6	0.2	17.8	1.8	11.2	0.4	17.4	1.4	9.1
ALA11	4.0	1.8	17.1	1.4	12.3	0.8	17.8	2.0	8.0
control	0	-	18.7	-	8.7	-	19.5	-	14.2

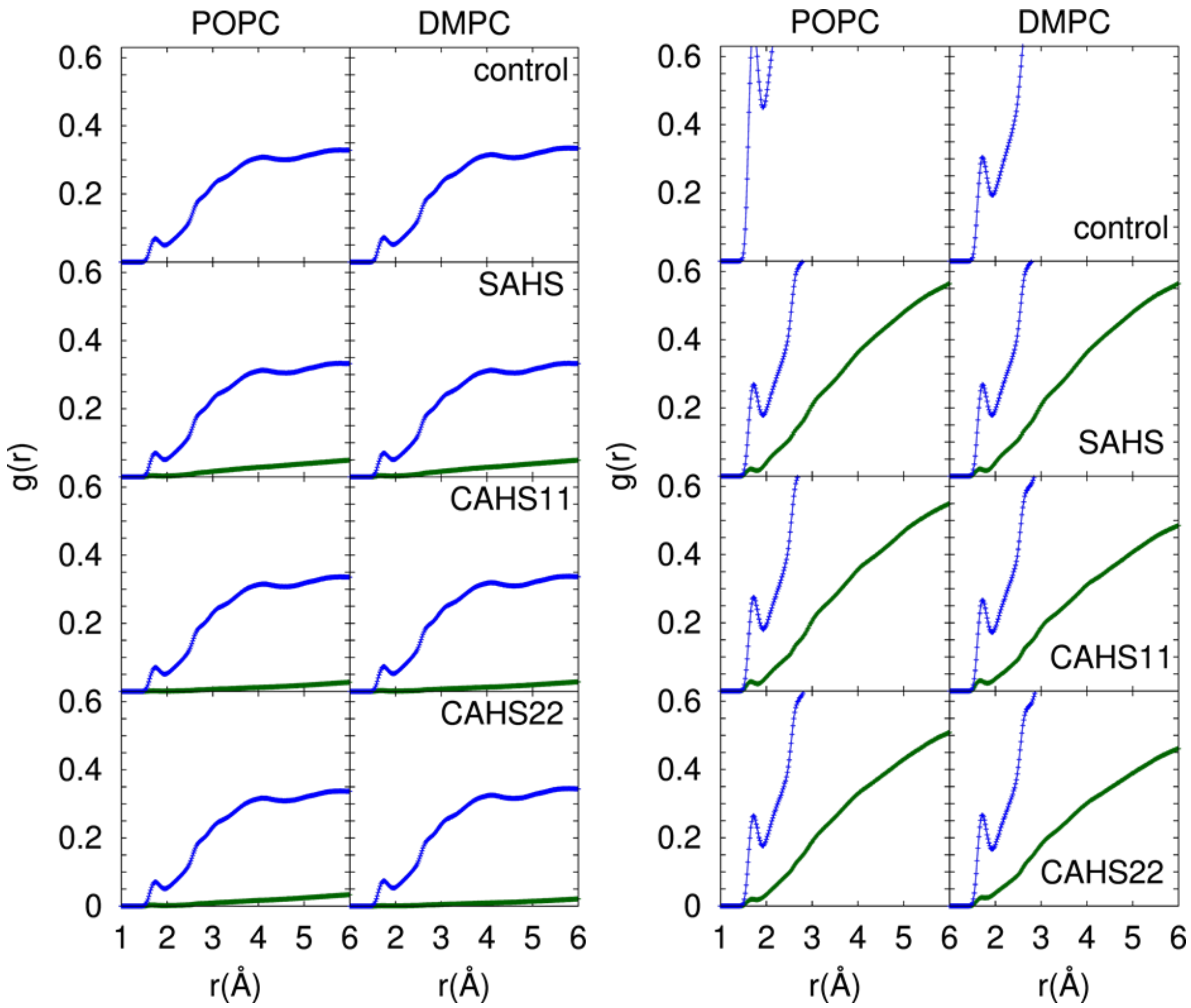

Figure S11. Radial distribution function (RDF) plots between the water solvent molecules (blue) and the TDPs (green), with respect to the atoms from the POPC and DMPC bilipid layers for the membranes embedded in pure water (control) and solvated TDP environments, within their respective full solvated (left) and dry (right) scenarios. All simulations were performed at room temperature (T=300K). Only distances from the membrane atoms lower than 6Å were plotted here.

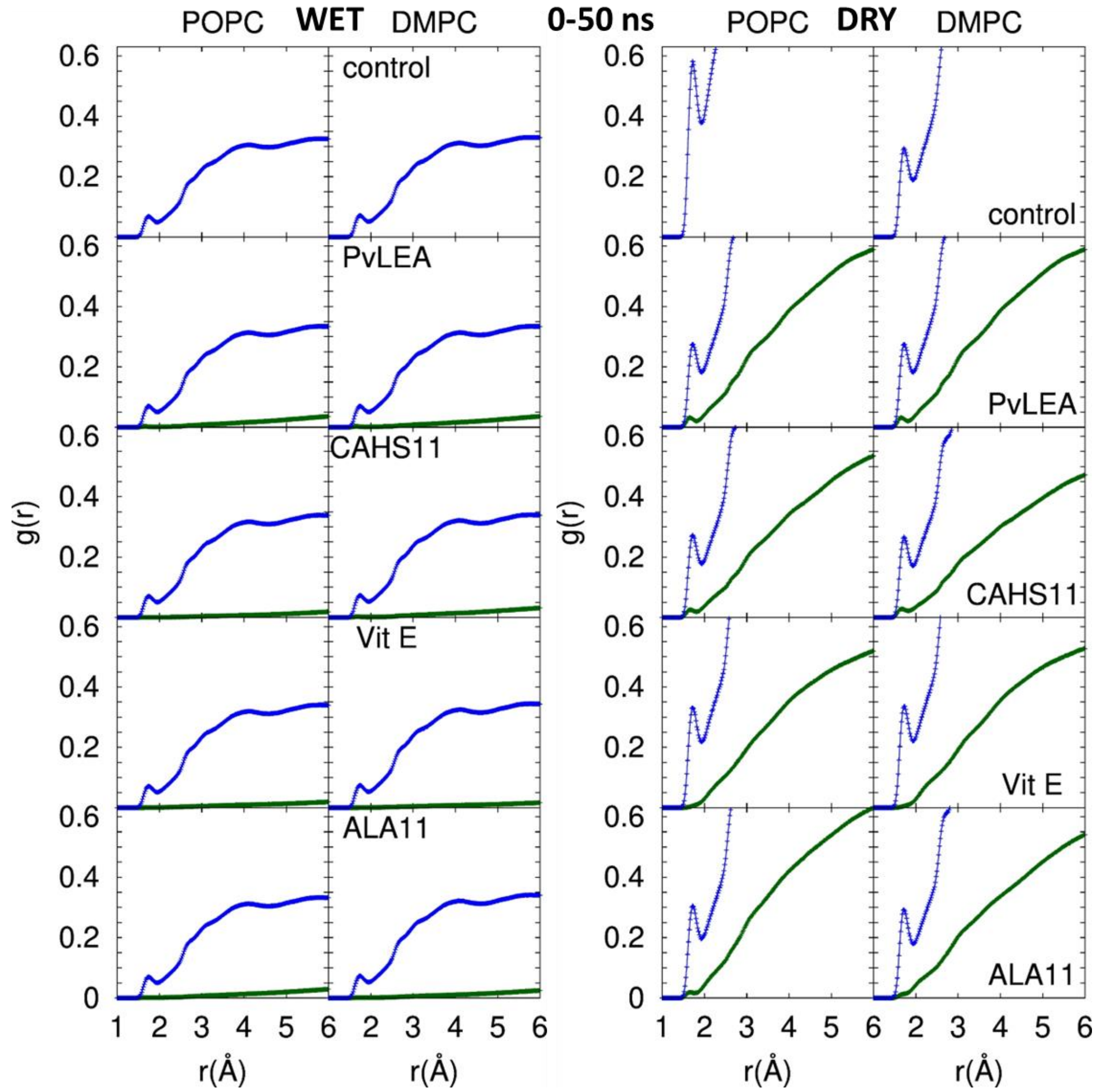

Figure S12. Radial distribution function (RDF) plots between the water solvent molecules (blue) and the xeroprotectant (green), with respect to the atoms from the POPC and DMPC lipid bilayers for the membranes embedded in pure water (control) and solvated representative xeroprotectant (LEA, TDPs, vitamin E) and apolar peptide environments, within their respective full solvated (left) and dry (right) scenarios, as considering the first halve (0-50 ns) of the MD trajectory. All simulations were performed at room temperature (T=300K). Only distances from the membrane atoms lower than 6Å were plotted here.

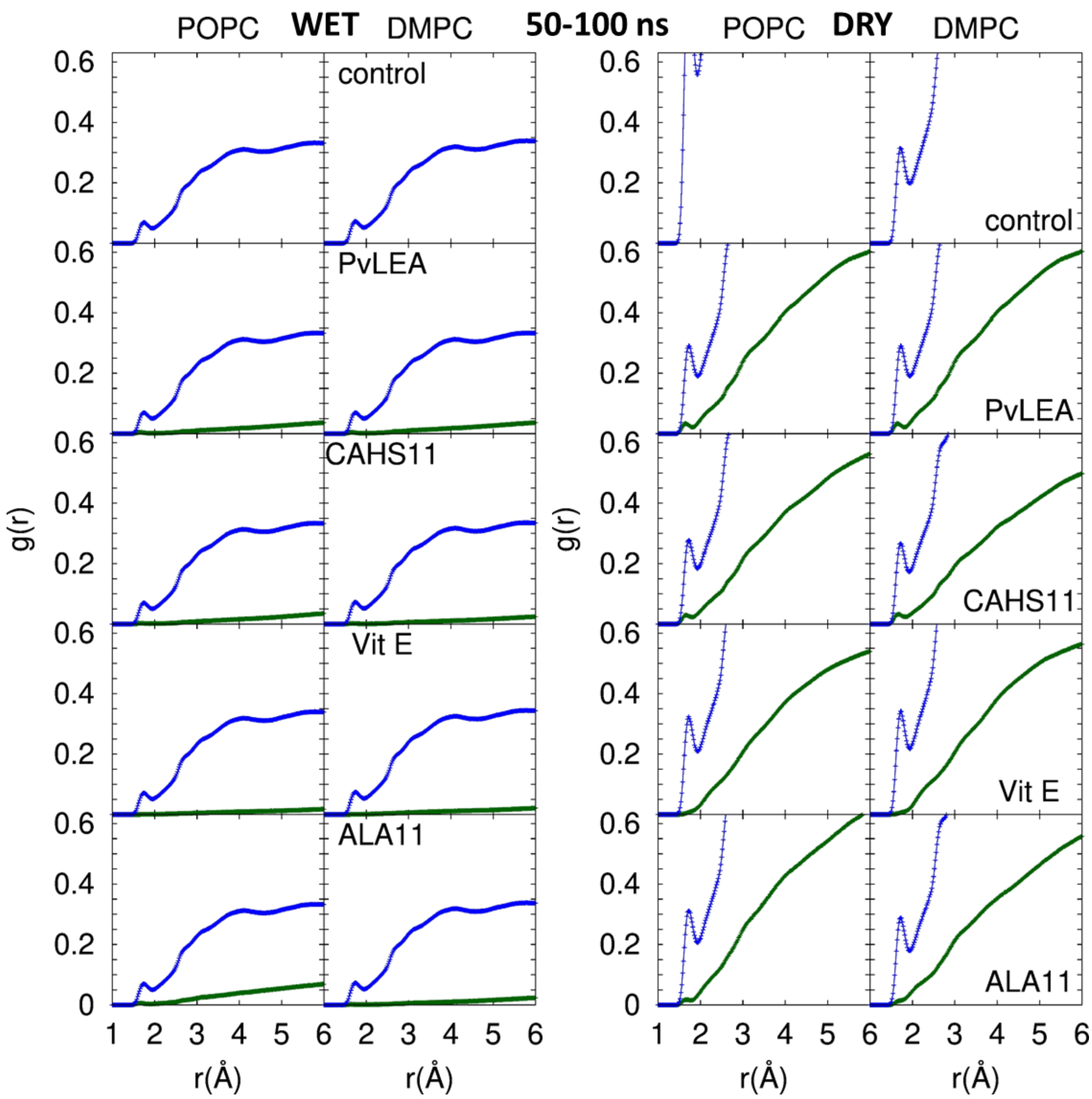

Figure S13. Radial distribution function (RDF) plots between the water solvent molecules (blue) and the xeroprotectant (green), with respect to the atoms from the POPC and DMPC lipid bilayers for the membranes embedded in pure water (control) and solvated representative xeroprotectant (LEA, TDPs, vitamin E) and apolar peptide environments, within their respective full solvated (left) and dry (right) scenarios, as considering the second halve (50-100 ns) of the MD trajectory.. All simulations were performed at room temperature (T=300K). Only distances from the membrane atoms lower than 6Å were plotted here.

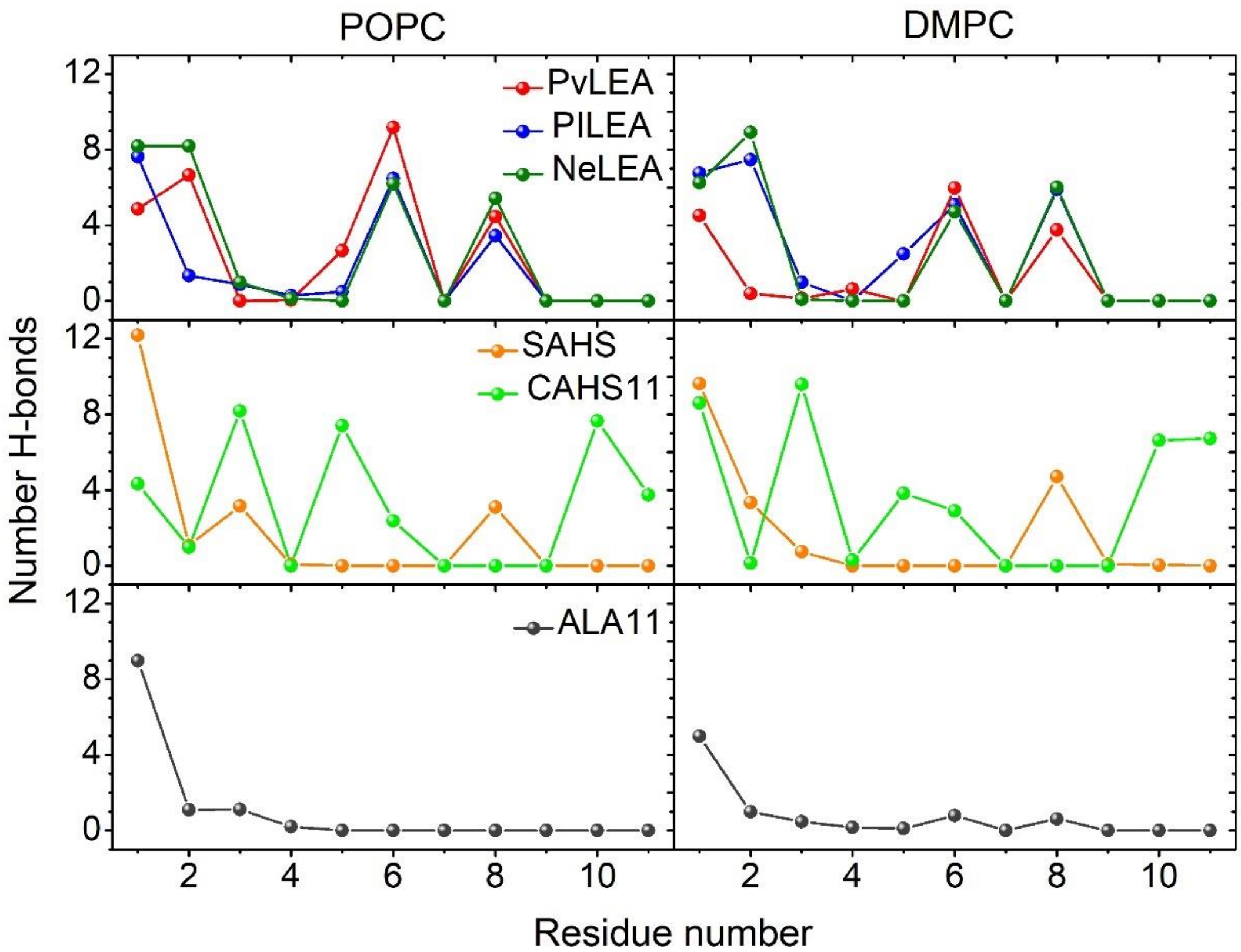

Figure S14. Number of H-bonds formed between the POPC (left) and DMPC (right) bilipid layer atoms and the amino acid residues conforming the 11-mer model peptide proteins (LEA, TDP and reference apolar proteins).

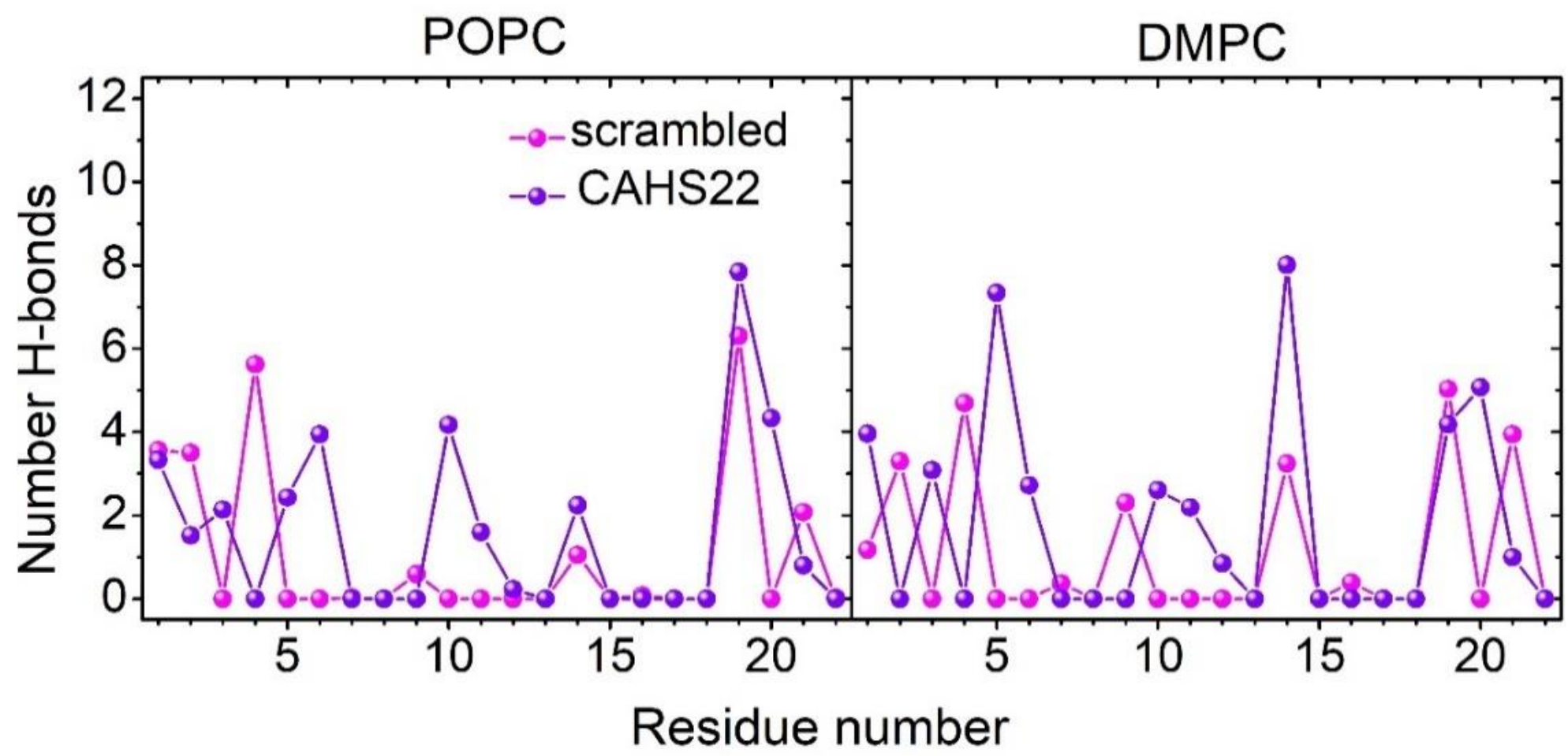

Figure S15. Number of H-bonds formed between the POPC (left) and DMPC (right) bilipid layer atoms and the amino acid residues conforming the 22-mer model peptide proteins (scrambled LEA and CAHS22 TDP).

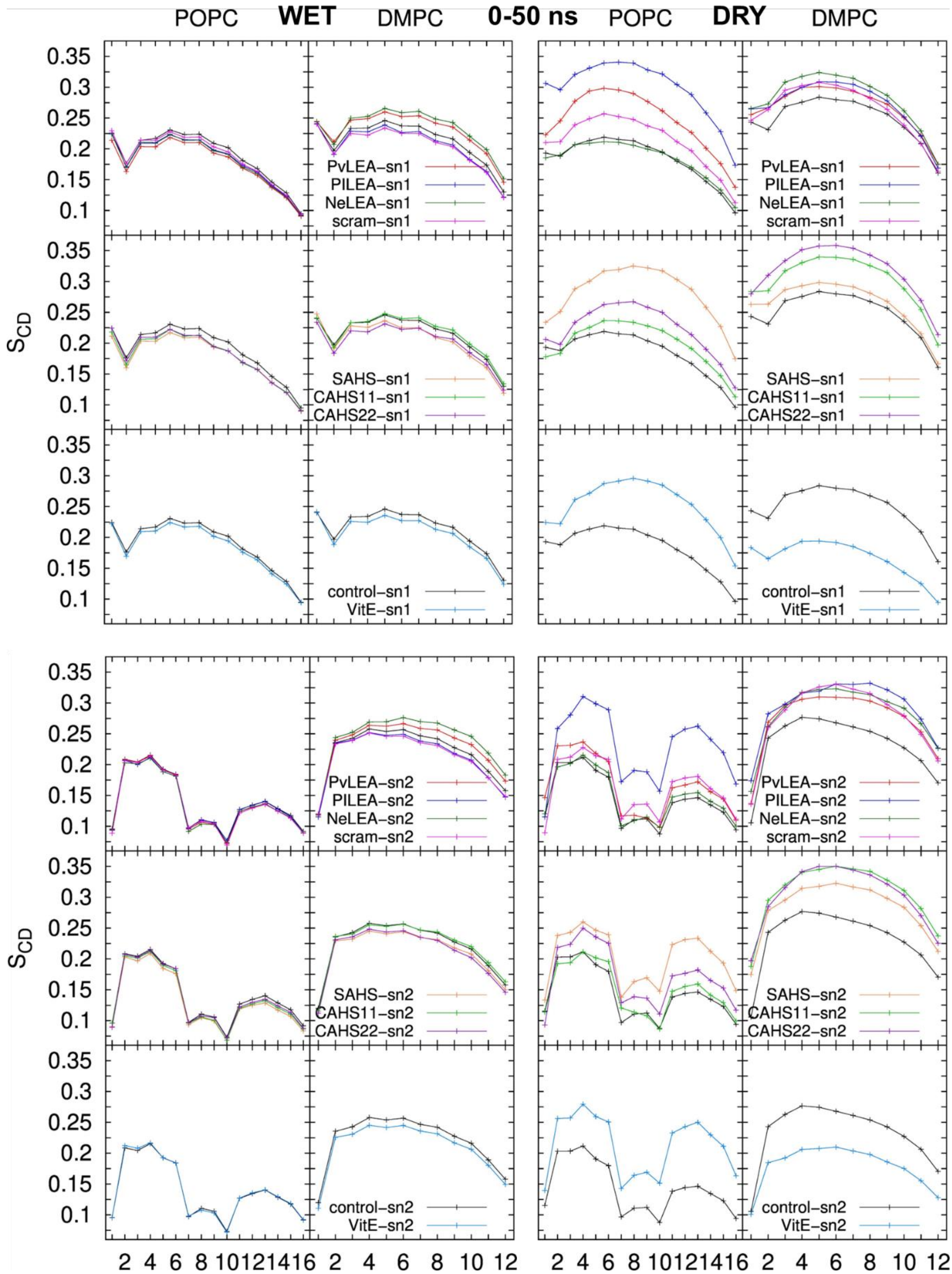

Figure S16. Deuterium order parameters S_{CD} of the carbon atoms belonging to two lipid acyl chains (sn1 and sn2 for top and bottom graphs, respectively) from the POPC and DMPC lipid bilayers integrating the membrane cell models embedded in pure water (control) and solvated xeroprotectant (LEA peptides, TDPs and vitamin E from the top to the bottom panels)

environments, within their respective full solvated (wet, right part) and desiccated (dry, right part) scenarios, as considering the first halve (0-50 ns) of the MD trajectory. All these simulations were carried out at room temperature (T=300K).

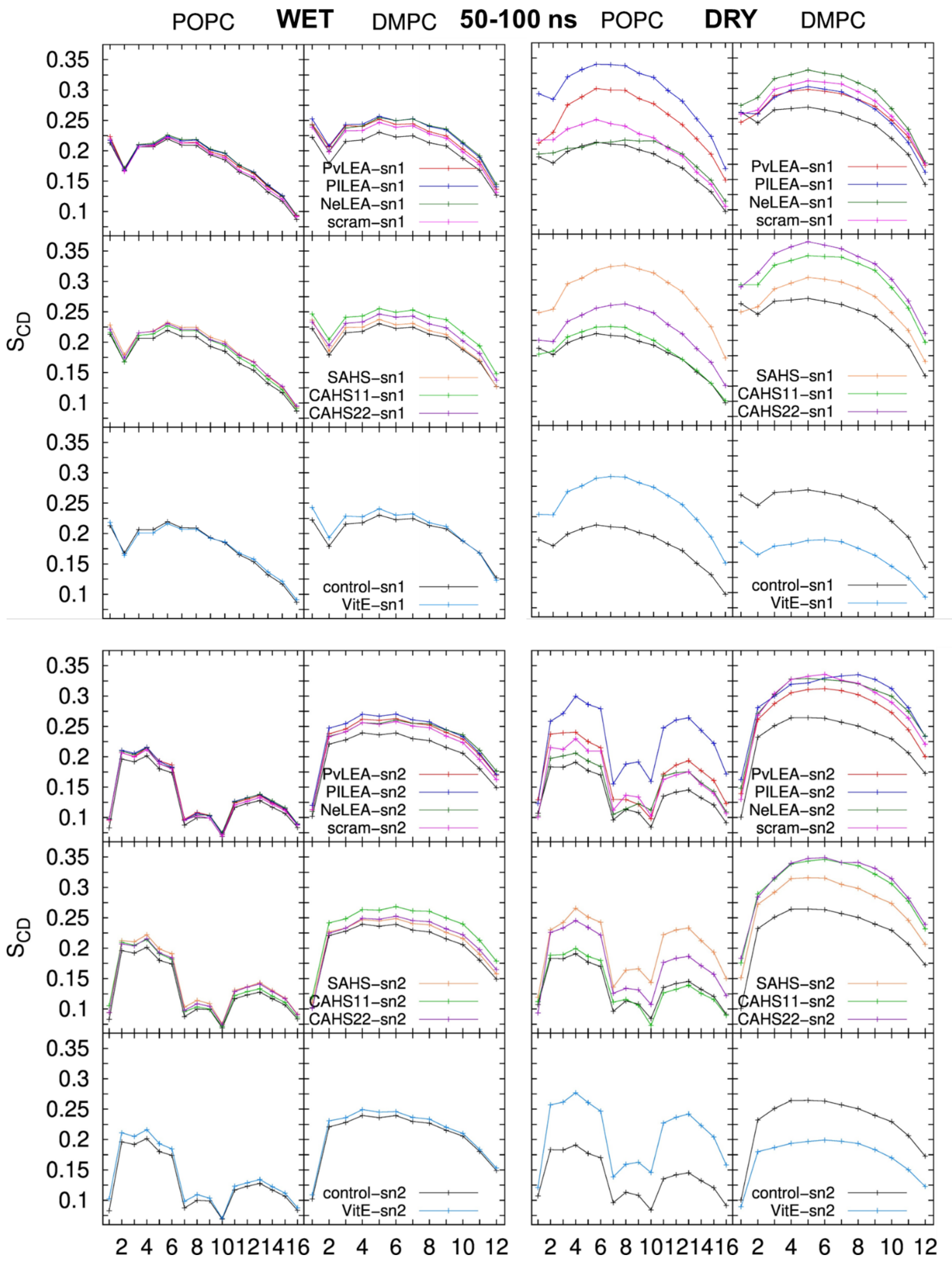

Figure S17. Deuterium order parameters S_{CD} of the carbon atoms belonging to two lipid acyl chains (sn1 and sn2 for top and bottom graphs, respectively) from the POPC and DMPC lipid bilayers integrating the membrane cell models embedded in pure water (control) and solvated

xeroprotectant (LEA peptides, TDPs and vitamin E from the top to the bottom panels) environments, within their respective full solvated (wet, right part) and desiccated (dry, right part) scenarios, as considering the second halve (50-100 ns) of the MD trajectory. All these simulations were carried out at room temperature (T=300K).

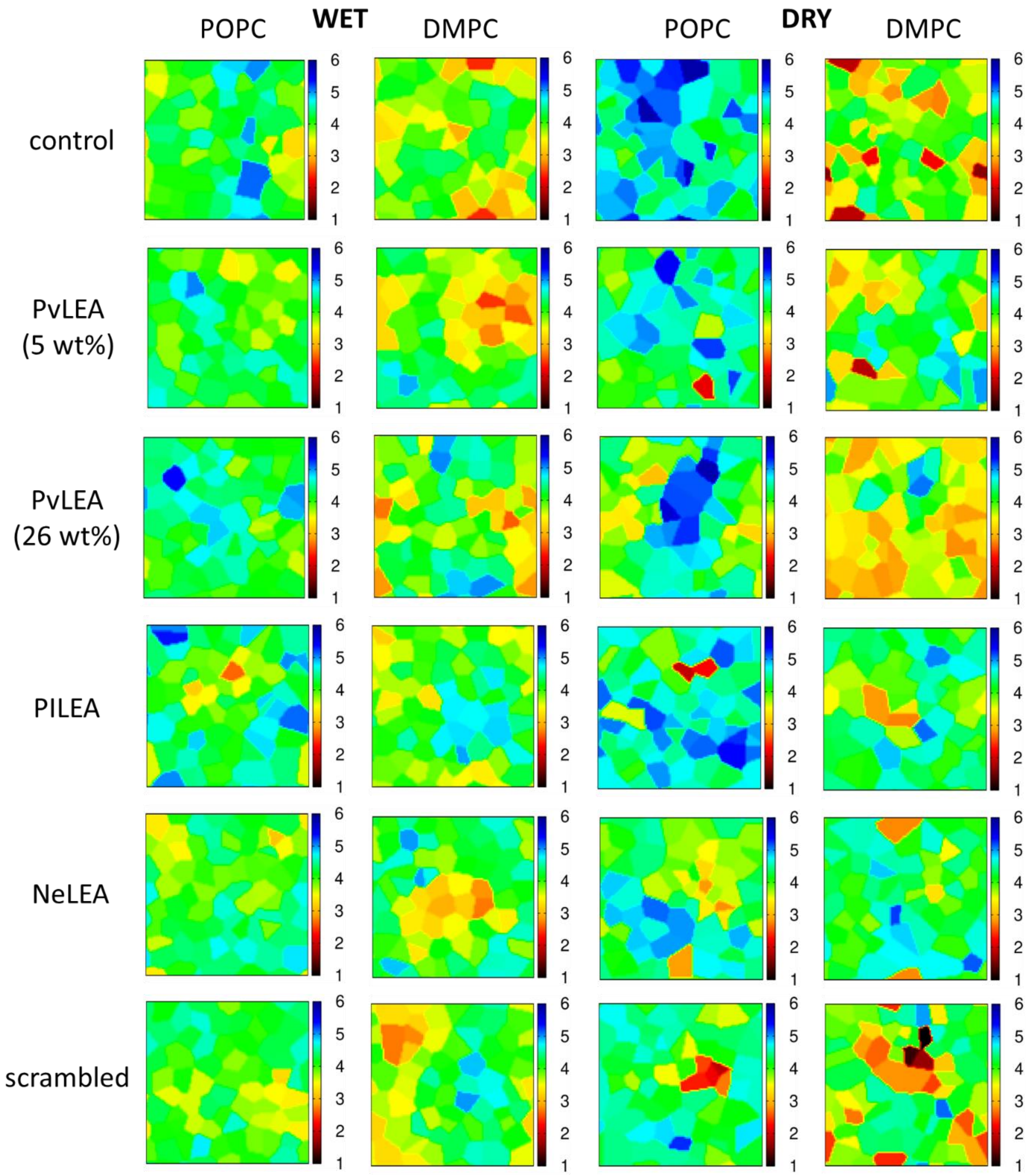

Figure S18. 2-D maps along the membrane section of the averaged bilayer thickness (in nm, as indicated in the color scale) for the last frame of the MD simulations performed at T=300K for the POPC and DMPC membranes embedded in pure water (control) and solvated LEA protein environments (from the top to the bottom), within their respective full solvated (wet, right part) and desiccated (dr, right part) scenarios.

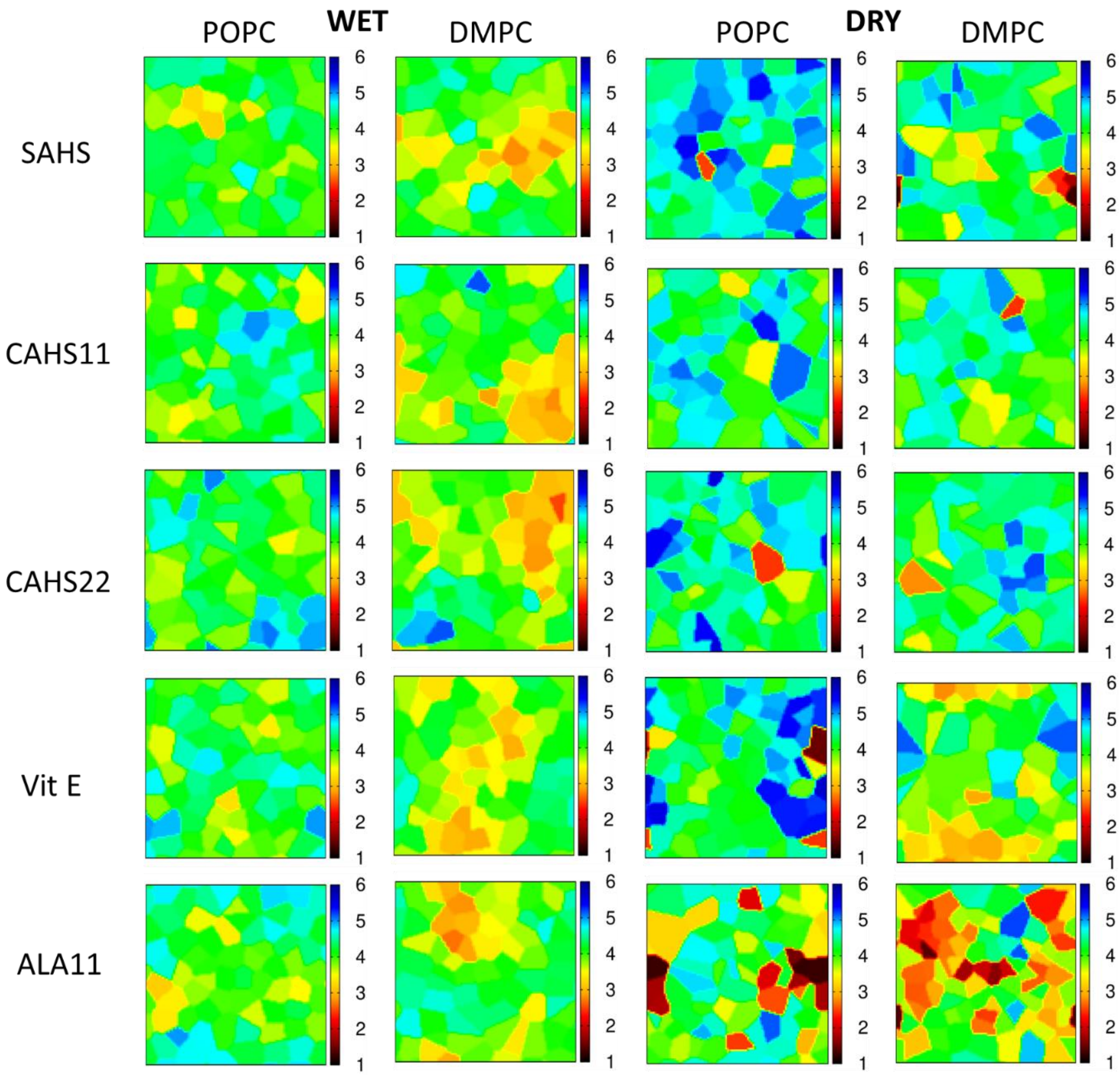

Figure S19. 2-D maps along the membrane section of the averaged bilayer thickness (in nm, as indicated in the color scale) for the last frame of the MD simulations performed at T=300K for the POPC and DMPC membranes embedded in pure water (control) and solvated TDPs, vitamin E and reference apolar protein (ALA11) environments (from the top to the bottom), within their respective full solvated (wet, right part) and desiccated (dry, right part) scenarios.

Table S3. Mechanical properties extracted from the MD simulations of the POPC and DMPC cell membranes embedded in solvated LEA proteins, TDPs, vitamin E, reference apolar protein (ALA11) and pure water (control) environments, within the full solvated (wet) and desiccated (dry) scenarios: lateral surface tension (γ), and compressibility modulus (K_A).

		POPC				DMPC			
		wet		dry		wet		dry	
Xero	wt%	γ_z (N/m)	K_A(J/m^2)	γ_z (N/m)	K_A(J/m^2)	γ_z (N/m)	K_A(J/m^2)	γ_z (N/m)	K_A(J/m^2)
PvLEA	5.5	0.78	0.38	0.30	0.75	-2.05	0.21	3.03	3.36
PvLEA	25.9	-1.99	0.28	2.67	4.21	0.18	0.18	2.80	1.08
PlLEA	5.1	4.33	0.15	-3.58	0.56	-3.11	0.18	1.83	0.70
NeLEA	5.4	-2.08	0.19	-5.43	2.33	0.99	0.28	-2.73	2.37
scrambled	7.2	-0.91	0.35	1.03	1.31	6.71	0.25	-4.61	4.06
SAHS	6.0	-3.00	0.16	0.66	3.43	1.01	0.21	-1.41	4.61
CAHS11	6.6	0.28	0.24	-4.28	4.67	3.77	0.10	1.00	3.51
CAHS22	8.5	-1.52	0.24	-3.60	1.73	0.88	0.24	0.62	1.19
Vitamin E	5.6	-0.61	0.16	-0.70	2.39	-2.68	0.32	1.85	6.84
ALA11	4.0	-3.68	0.36	5.01	0.41	-2.03	0.26	-2.34	0.70
control	0	0.36	0.20	-1.00	0.15	2.65	0.17	1.96	0.64

Table S4. Standard deviations for the structural properties of the LEA peptides and TDPs in the solvated interfaces with the POPC and DMPC lipid bilayers within the full solvated (wat) and desiccated (dry) environments, as presented in Figure 4 of the main text: root-mean-square displacement (RMSD) in Å, radius of gyration (R_g) in Å, and number of H-bonds formed within the peptide structure.

	POPC						DMPC					
	wat			dry			wat			dry		
Xero	RMSD	R_g	H_b	RMSD	R_g	H_b	RMSD	R_g	H_b	RMSD	R_g	H_b
PvLEA	1.15	0.89	18	0.15	0.10	12	0.83	0.84	18	0.21	0.07	23
PlLEA	1.10	1.01	21	0.17	0.10	11	0.67	0.67	15	0.13	0.08	19
NeLEA	0.97	0.64	19	0.18	0.07	12	1.10	0.98	18	0.15	0.06	23
scrambled	1.27	0.55	32	0.19	0.10	16	1.25	0.69	35	0.11	0.08	18
SAHS	0.91	0.54	17	0.20	0.10	11	0.95	0.67	18	0.24	0.13	22
CAHS11	0.85	0.34	19	0.13	0.06	14	0.74	0.49	19	0.16	0.08	29
CAHS22	0.93	0.38	27	0.21	0.11	19	0.64	0.23	23	0.14	0.10	17
ALA11	0.98	1.04	14	0.14	0.11	13	0.85	0.87	14	0.24	0.17	9

Table S5. Standard deviations for the aggregation properties of the LEA peptides, TDPs, vitamin E xeroprotectants in the solvated interfaces with the POPC and DMPC lipid bilayers within the full solvated (wat) and desiccated (dry) environments, as presented in Table 5 of the main text: number of clusters (N_{clust}), averaged (N_{avg}) and maximum (N_{max}) cluster size in terms of number of molecules integrating the cluster.

	POPC						DMPC					
	wat			dry			wat			dry		
Xero	N_{clust}	N_{avg}	N_{max}	N_{clust}	N_{avg}	N_{max}	N_{clust}	N_{avg}	N_{max}	N_{clust}	N_{avg}	N_{max}
PvLEA	1.06	1.76	1.20	0.48	0.24	0.48	1.09	1.60	1.28	0.53	0.15	0.53
PlLEA	1.18	1.09	1.16	0.18	0.05	0.18	1.17	1.34	1.22	0.06	0.02	0.06
NeLEA	1.01	1.59	1.22	0.62	0.26	0.46	1.07	1.07	1.15	0.36	0.19	0.20
scrambled	0.68	0.91	0.71	0.73	0.40	0.73	0.80	1.06	0.82	0.40	0.76	0.48
SAHS	1.04	1.43	1.24	0.58	0.19	0.55	1.07	1.59	1.34	1.05	0.40	0.74
CAHS11	1.19	1.76	1.41	0.10	0.02	0.10	1.19	1.66	1.28	0.41	0.17	0.32
CAHS22	0.81	0.90	0.83	0.44	0.30	0.12	1.04	1.24	1.06	0.35	0.63	0.62
Vitamin E	0.21	0.98	0.56	0.32	2.53	1.89	0.55	1.83	1.03	0.28	1.60	1.14
ALA11	1.05	0.54	1.02	0.51	0.11	0.45	1.03	0.56	0.90	0.95	0.77	0.74

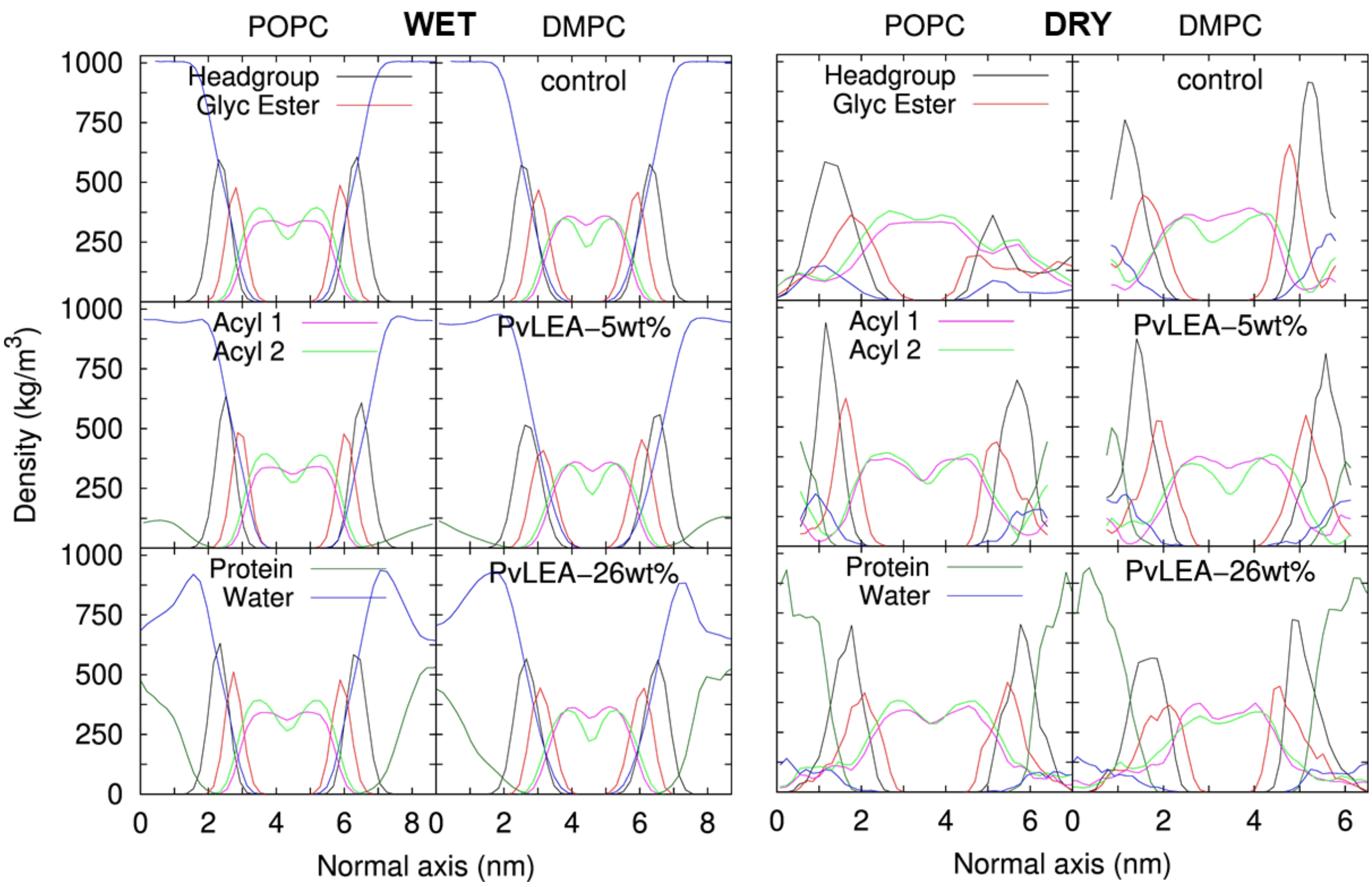

Figure S20. Average density profiles along the normal direction to membrane plane of the POPC and DMPC bilipid layer (phospholipid headgroups, glycerol ester, two acyl chains) and water solvent of the membranes embedded in pure water (control) and solvated PvLEA in low (5 wt%) and high (>25 wt%) concentrations, within their respective full solvated (left) and dry (right) scenarios. All simulations were performed at room temperature (T=300K).

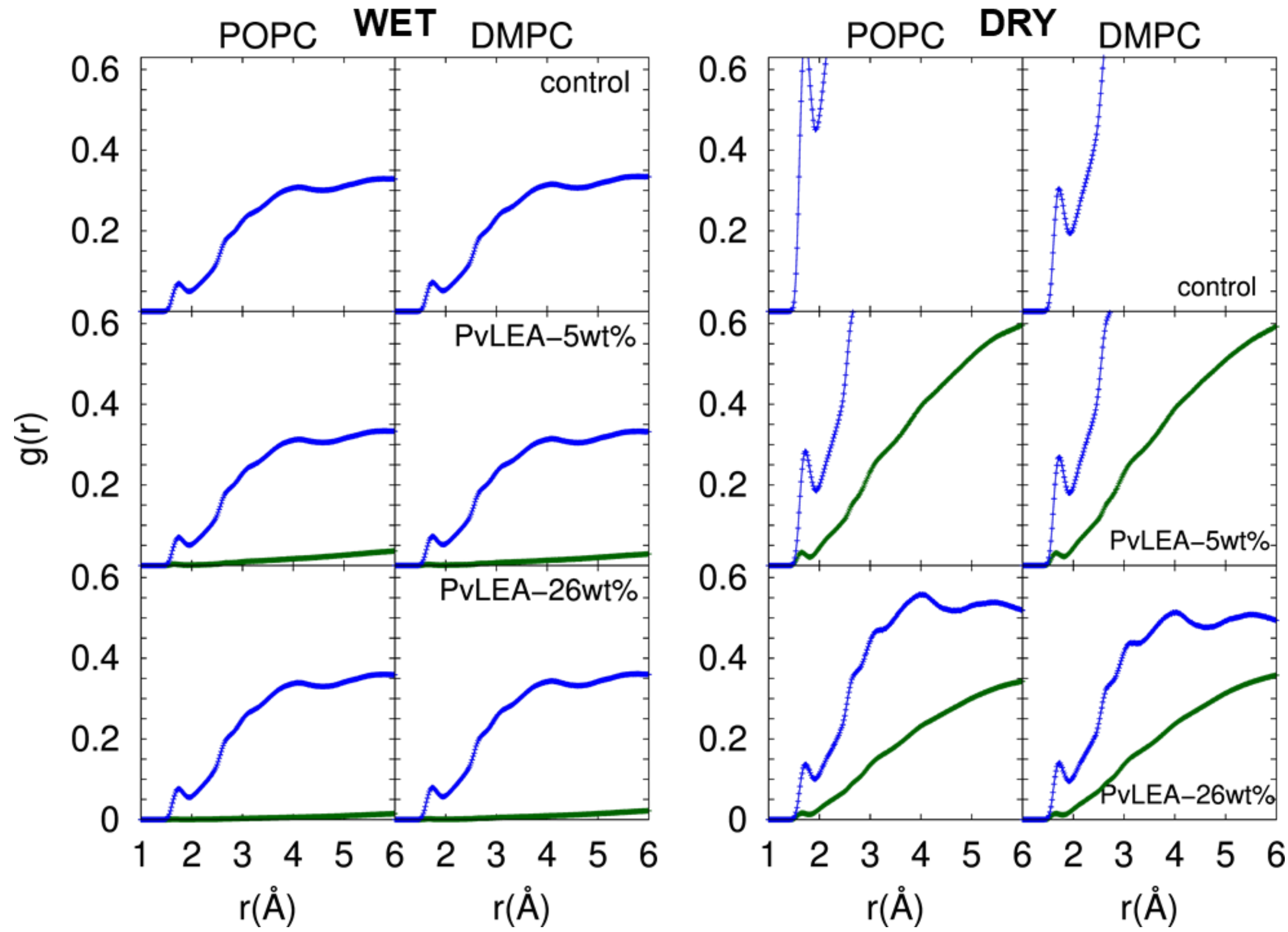

Figure S21. Radial distribution function (RDF) plots between the water solvent molecules (blue) and the TDPs (green), with respect to the atoms from the POPC and DMPC bilipid layers for the membranes embedded in pure water (control) and solvated PvLEA in low (5 wt%) and high (>25 wt%) concentrations, within their respective full solvated (left) and dry (right) scenarios. All simulations were performed at room temperature (T=300K). Only distances from the membrane atoms lower than 6Å were plotted here.

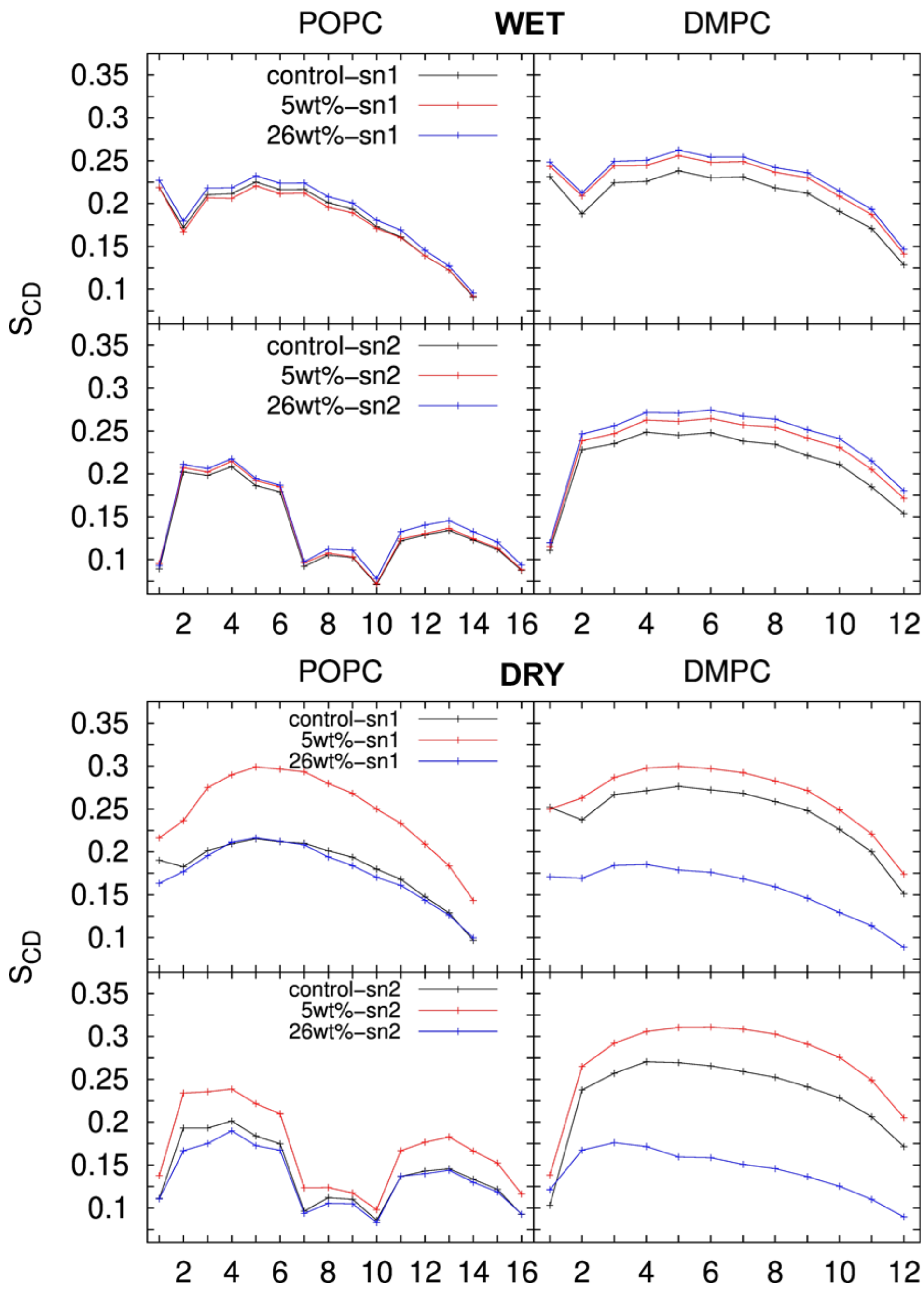

Figure S22. Deuterium order parameters S_{CD} of the carbon atoms belonging to two lipid acyl chains (sn1 and sn2 for top and bottom graphs, respectively) from the POPC and DMPC bilipid layers integrating the membrane cell models embedded in pure water (control) and solvated PvLEA in low (5 wt%) and high (26 wt%) concentrations, within their respective full solvated (wet, top part) and desiccated (dry, bottom part) scenarios. All these simulations were carried out at room temperature (T=300K).

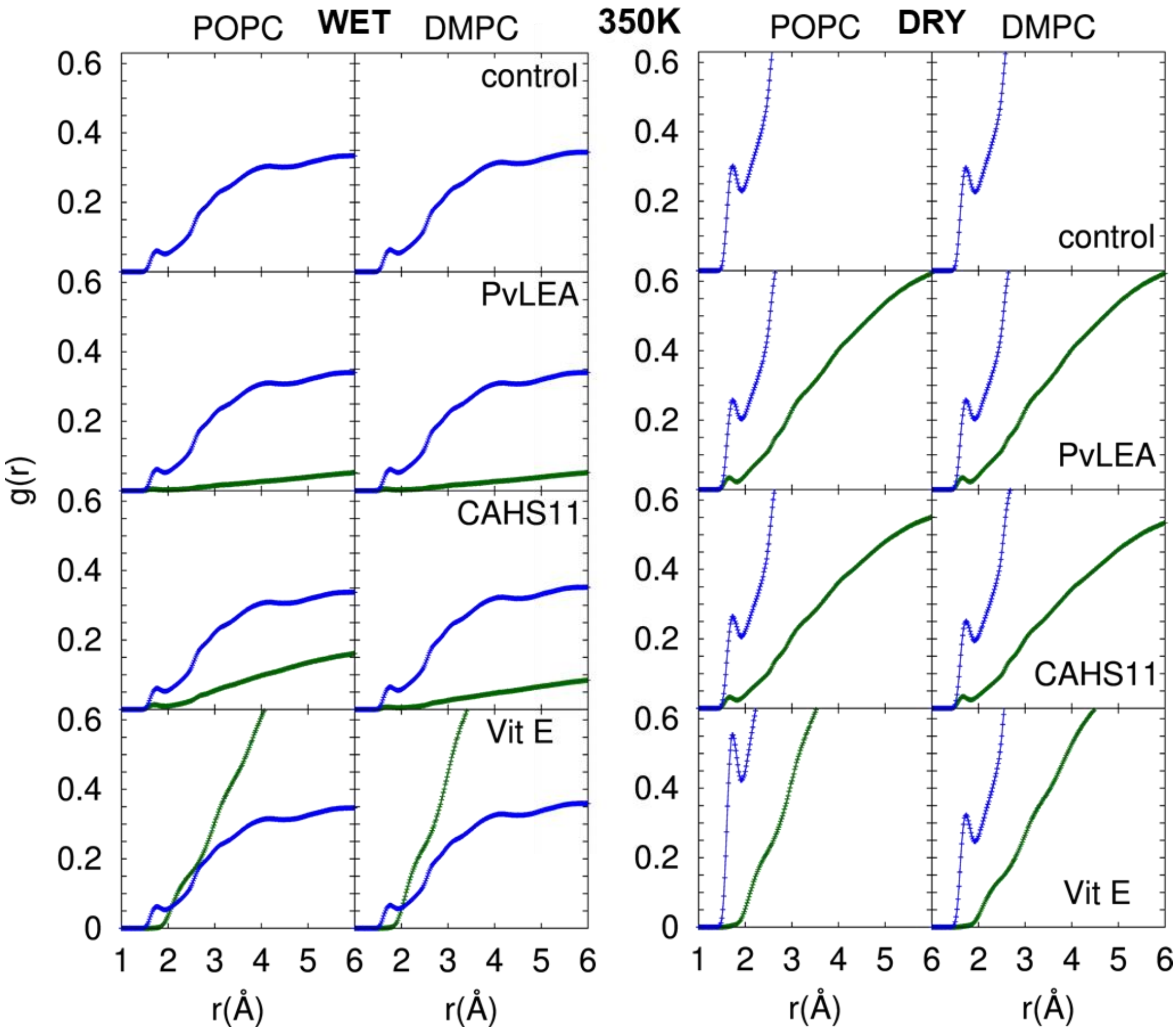

Figure S23. Radial distribution function (RDF) plots between the water solvent molecules (blue) and the TDPs (green), with respect to the atoms from the POPC and DMPC bilipid layers for the membranes embedded in pure water (control) and solvated PvLEA, CAHS11, and vitamin E environments, within their respective full solvated (left) and dry (right) scenarios, and for the starting high temperature (T=350K) of the cooling quench process. Only distances from the membrane atoms lower than 6Å were plotted here.

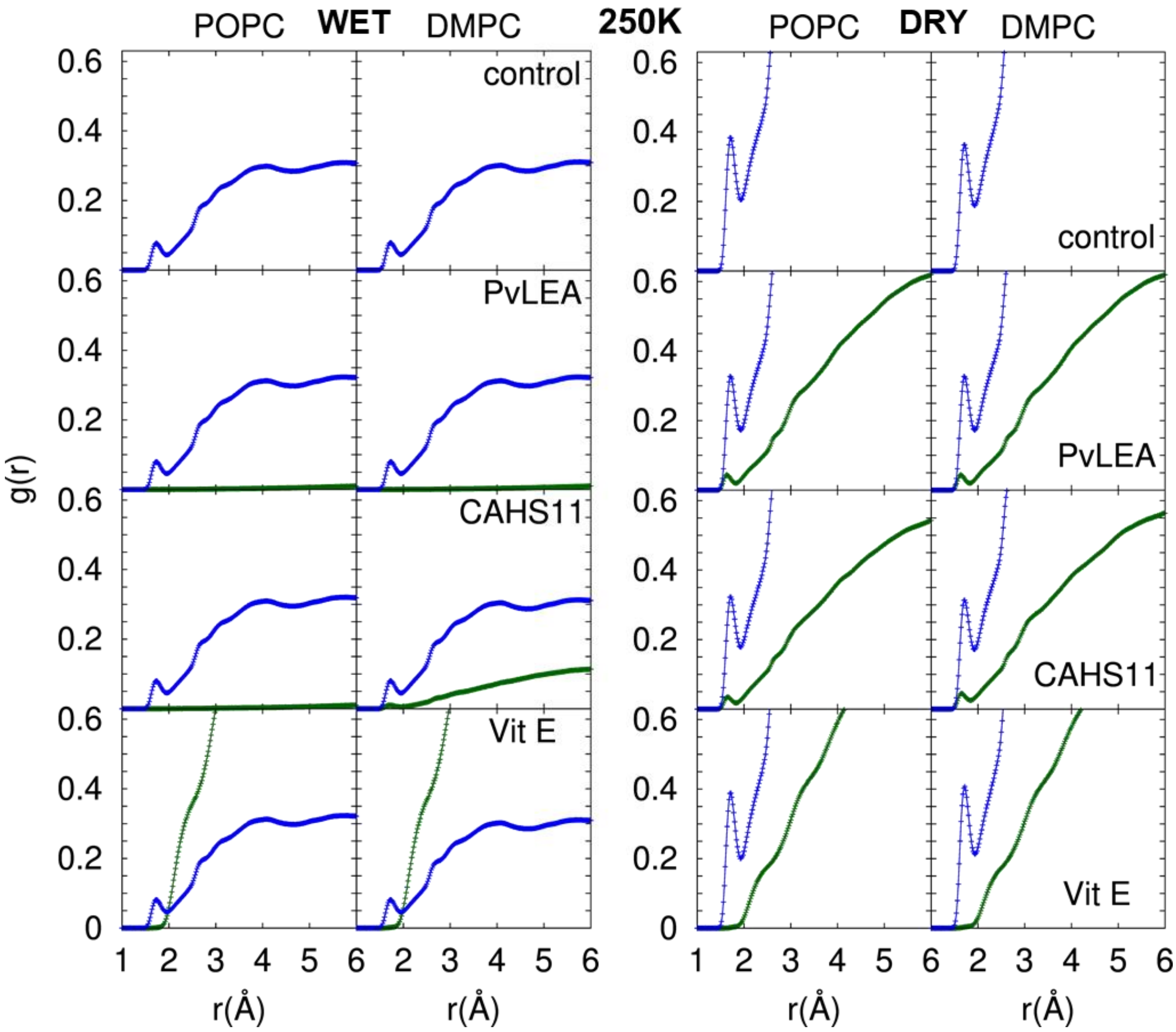

Figure S24. Radial distribution function (RDF) plots between the water solvent molecules (blue) and the TDPs (green), with respect to the atoms from the POPC and DMPC bilipid layers for the membranes embedded in pure water (control) and solvated PvLEA, CAHS11, and vitamin E environments, within their respective full solvated (left) and dry (right) scenarios, and for the final low temperature (T=250K) of the cooling quench process. Only distances from the membrane atoms lower than 6Å were plotted here.

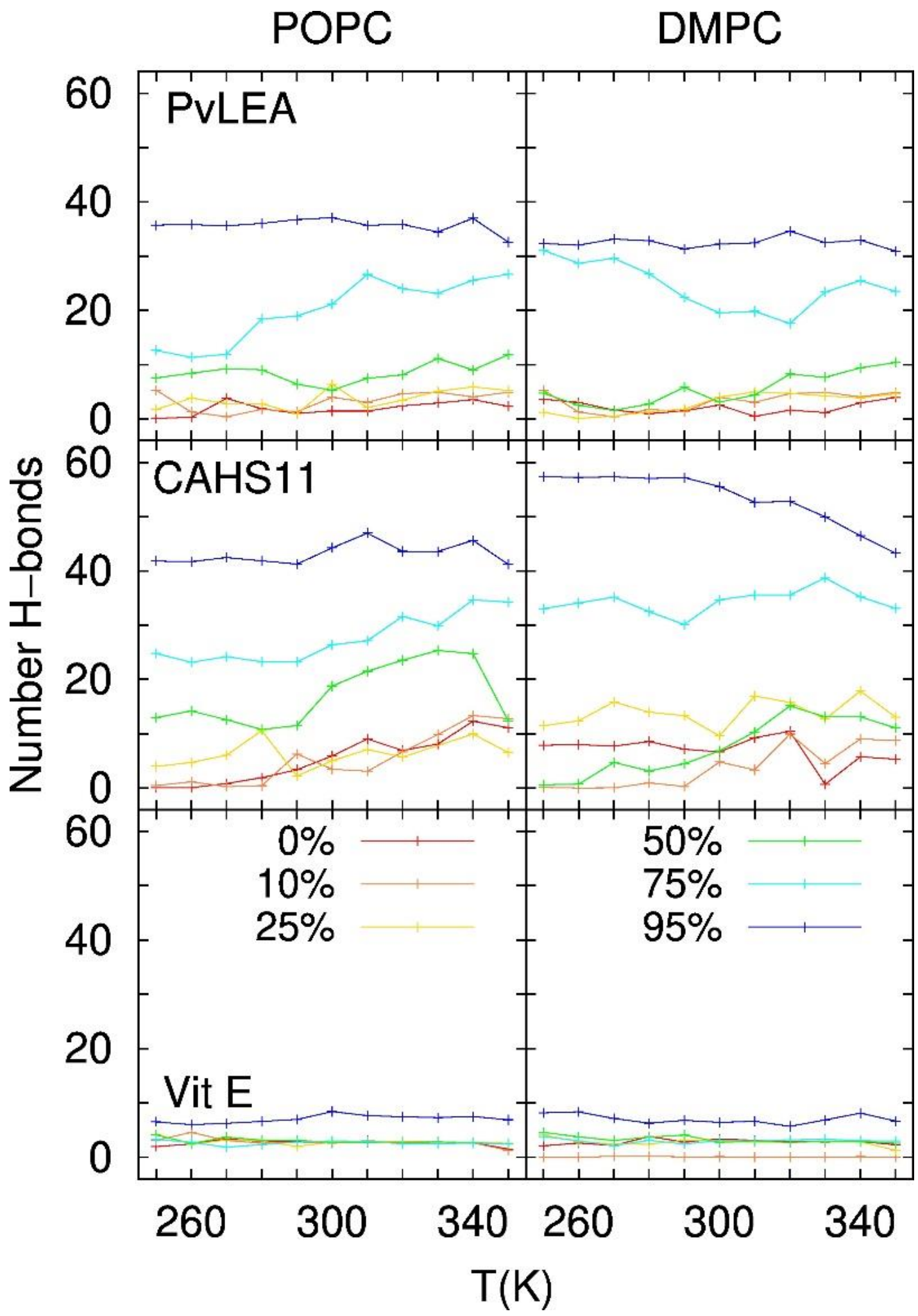

Figure S25. Number of H-bonds formed between the PvLEA, CAHS11, and vitamin E xeroprotectants (from the top to the bottom) and the atoms from the POPC and DMPC bilipid layers, by considering desiccated interfaces where 0% (red), 10% (orange), 25% (yellow), 50% (green), 75% (cyan) and 95% (dark blue) from the initial water molecules were removed, as a function of the temperature of the cooling quench process.

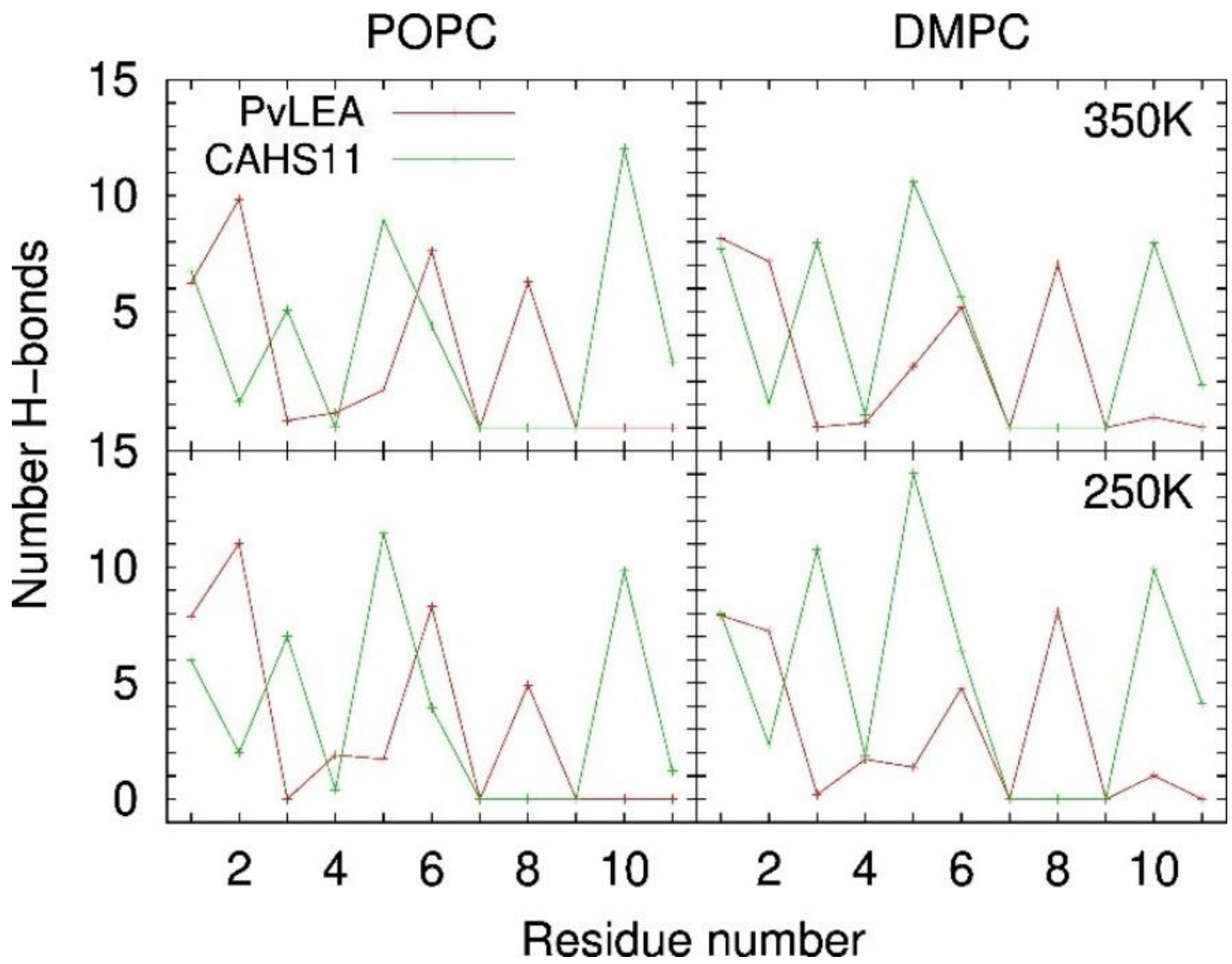

Figure S26. Number of H-bonds formed between the POPC (left) and DMPC (right) bilipid layer atoms and the amino acid residues conforming the 11-mer model peptide proteins PvLEA and CAHS11, for the two extreme temperatures from the quench simulations: 350K (top) and 250K (bottom panels).

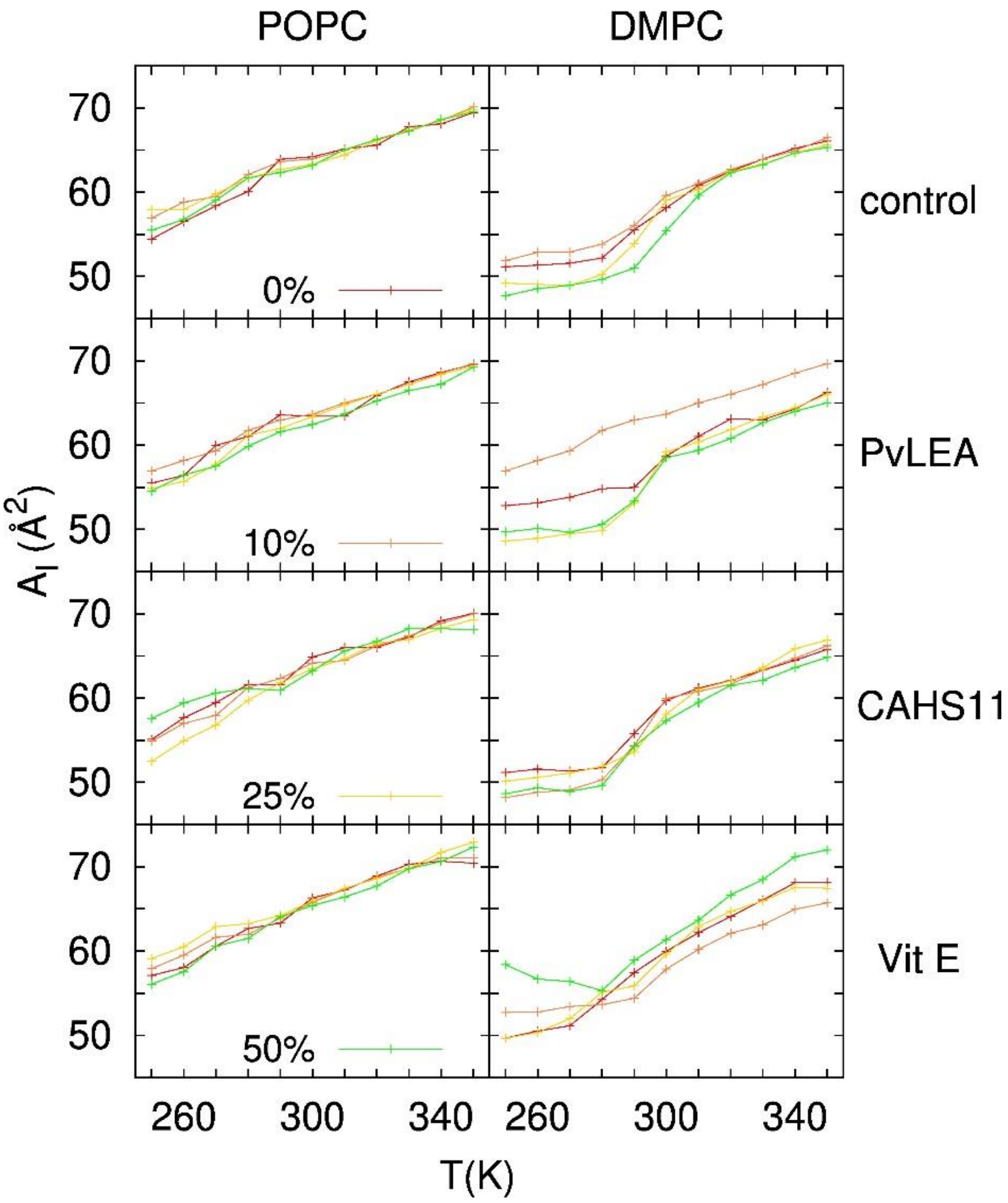

Figure S27. Area per lipid in Å^2 for the POPC and DMPC bilipid layers embedded in pure water (control) and solvated PvLEA, CAHS11, and vitamin E environments (from the top to the bottom), built by considering desiccated interfaces where 0% (red), 10% (orange), 25% (yellow) and 50% (green) from the initial water molecules were removed, as a function of the temperature of the cooling quench process. Note that areas from the interfaces with high dehydration ratios (75% and 95%) were not included here since the instability of the bilipid layers hindered the estimation of reliable lipid areas.

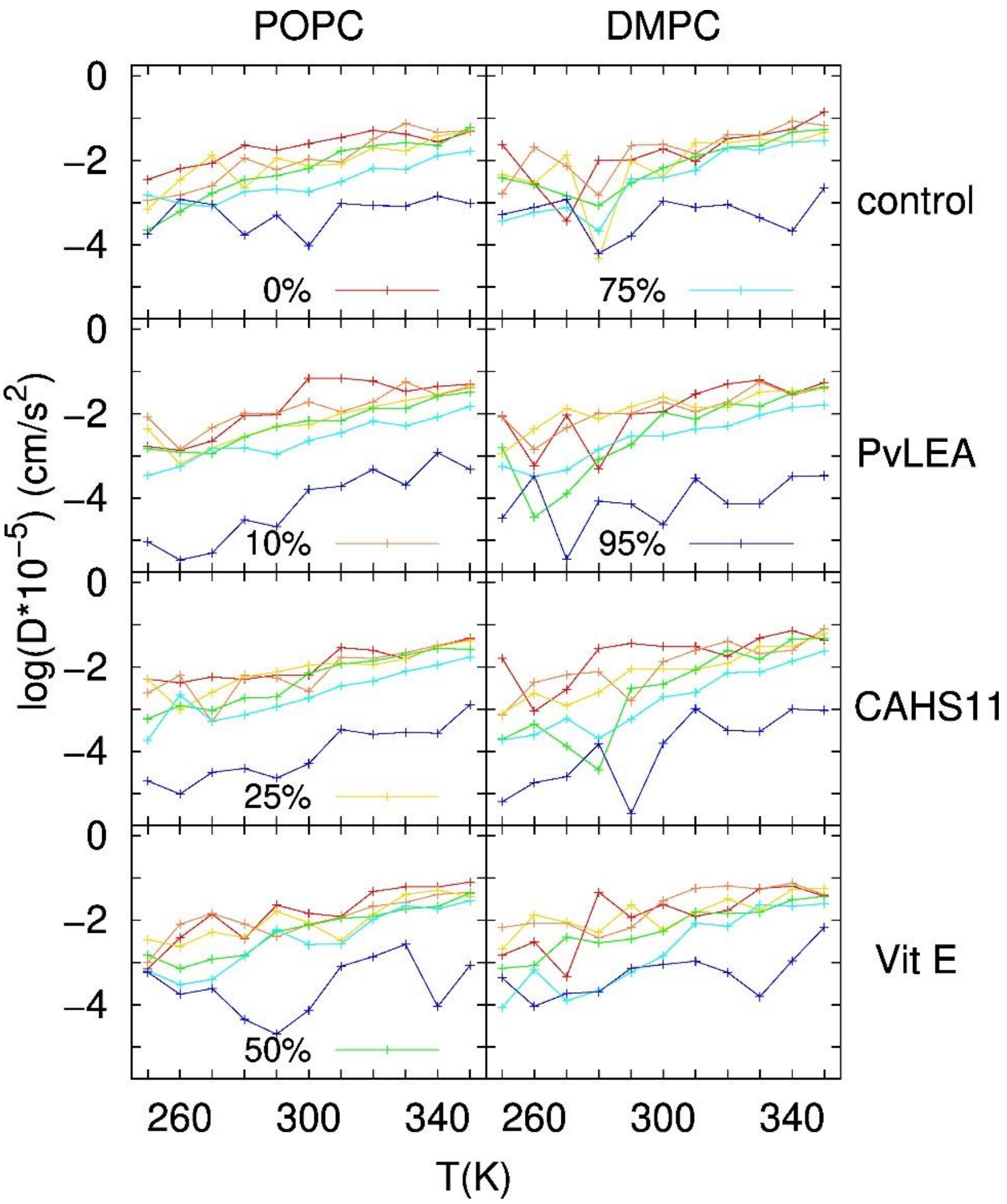

Figure S28. Lateral diffusivity of the P atoms from the lipid headgroups in logarithmic scale for the POPC and DMPC bilipid layers embedded in pure water (control) and solvated PvLEA, CAHS11, and vitamin E environments (from the top to the bottom), built by considering desiccated interfaces where 0% (red), 10% (orange), 25% (yellow), 50% (green), 75% (cyan) and 95% (dark blue) from the initial water molecules were removed, as a function of the temperature of the cooling quench process.

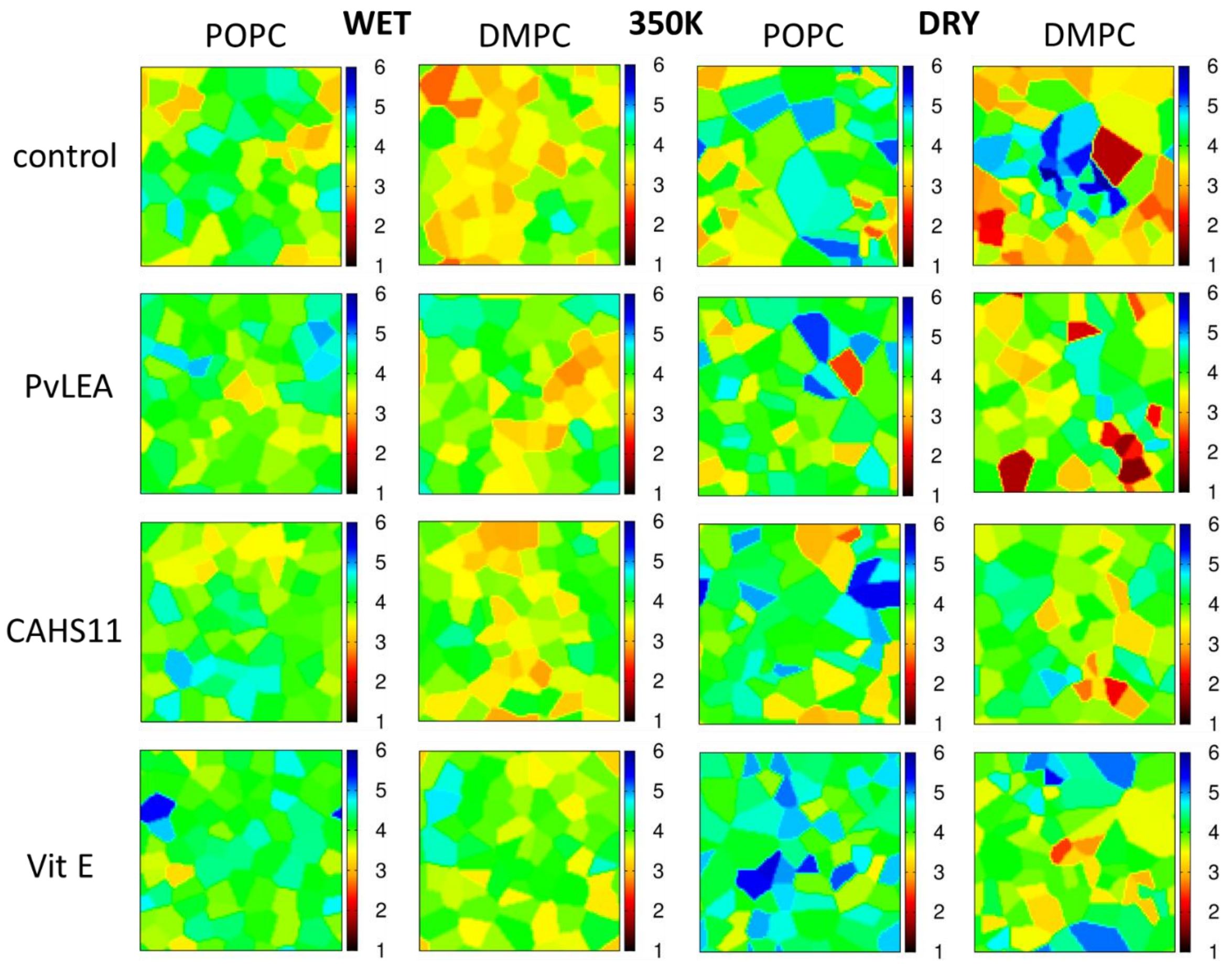

Figure S29. 2-D maps along the membrane section of the averaged bilayer thickness (in nm, as indicated in the color scale) for the last frame of the MD simulations performed at starting high temperature (T=350K) of the cooling quenching process for the POPC and DMPC membranes embedded in pure water (control) and solvated PvLEA, CAHS11, and vitamin E environments (from the top to the bottom), within their respective full solvated (wet, right part) and desiccated (dry, right part) scenarios.

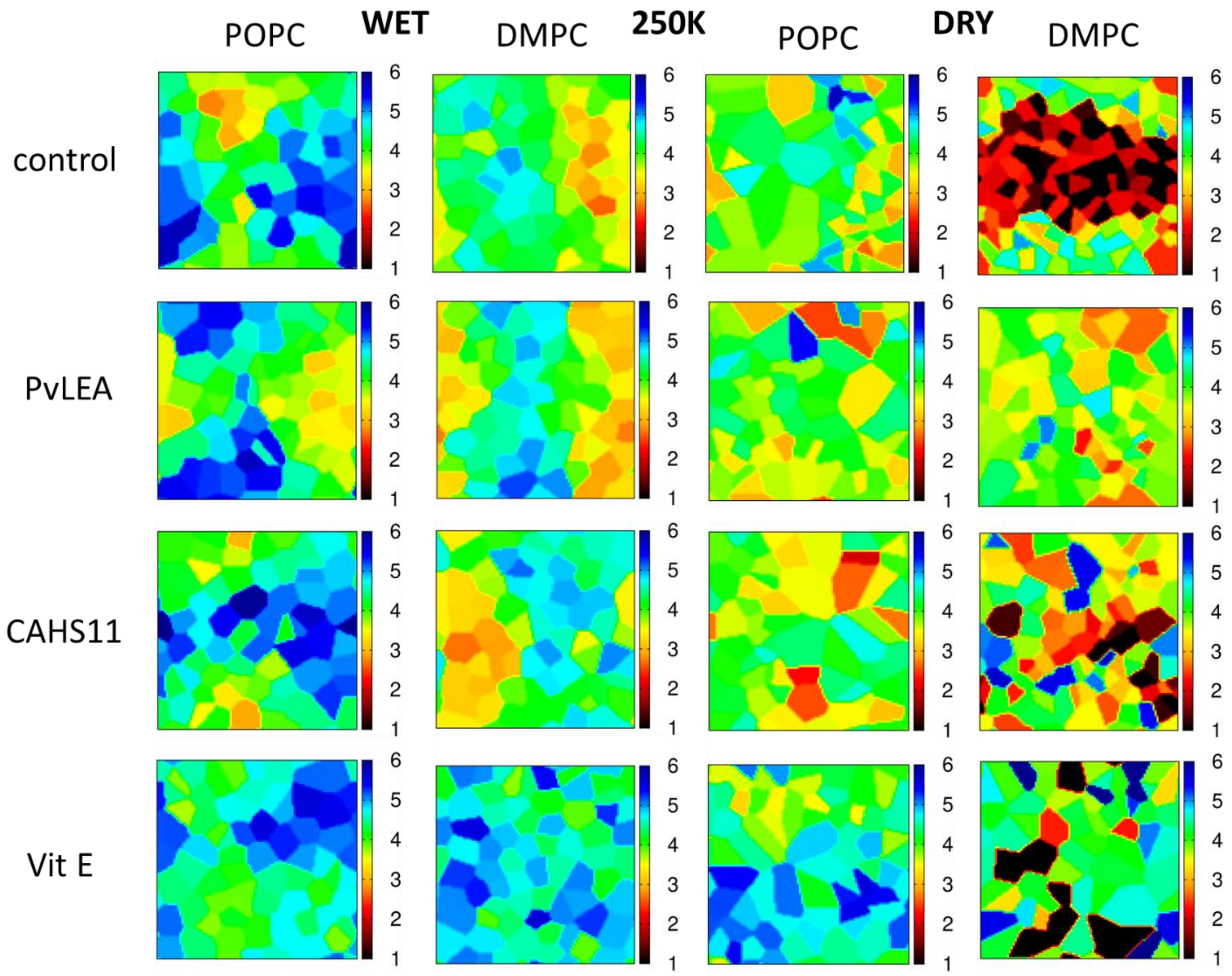

Figure S30. 2-D maps along the membrane section of the averaged bilayer thickness (in nm, as indicated in the color scale) for the last frame of the MD simulations performed at the final low temperature (T=250K) of the cooling quenching process for the POPC and DMPC membranes embedded in pure water (control) and solvated PvLEA, CAHS11, and vitamin E environments (from the top to the bottom), within their respective full solvated (wet, right part) and desiccated (dry, right part) scenarios.

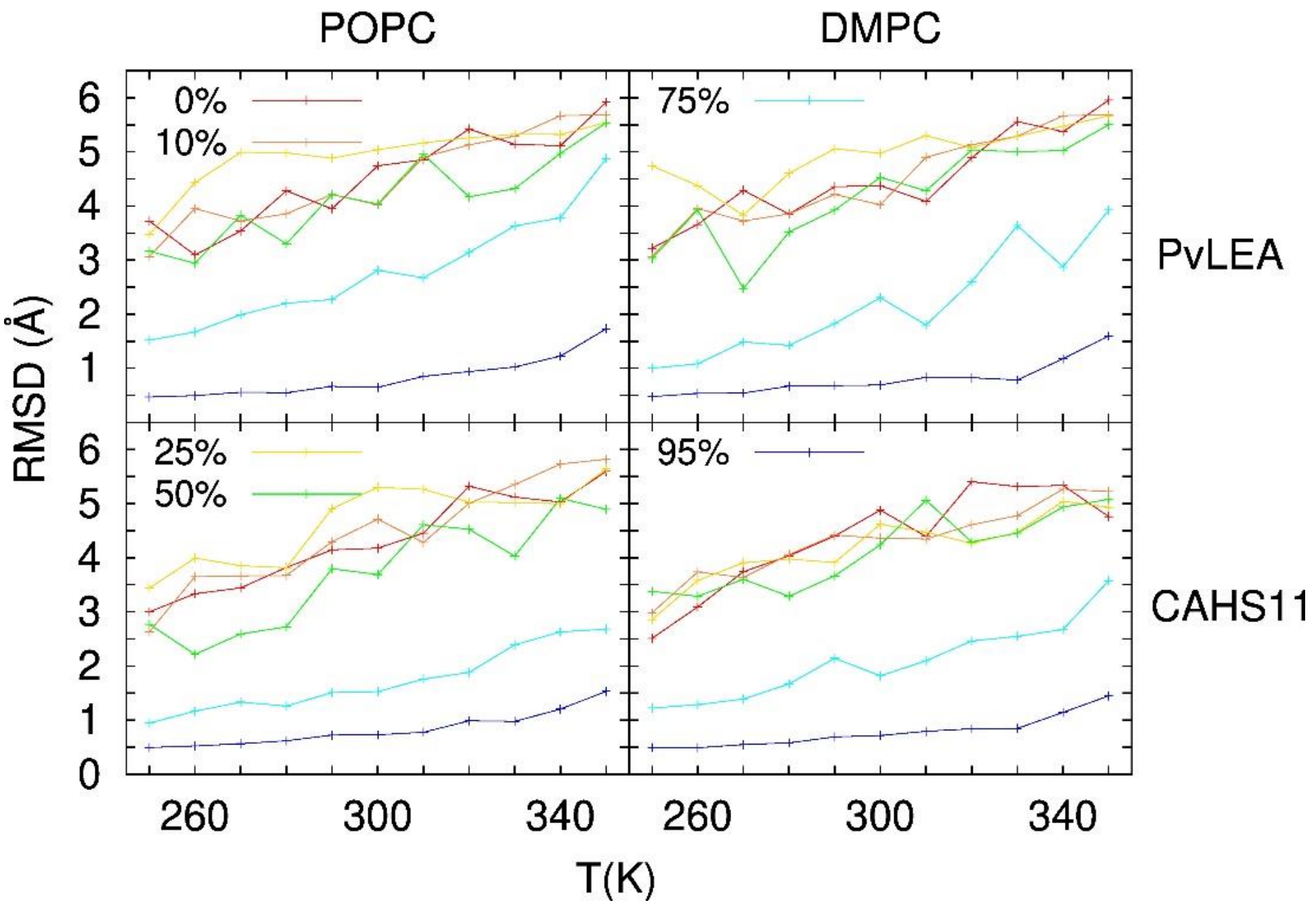

Figure S31. Root-mean square displacements (RMSDs) in Å for the PvLEA and CAHS11 proteins at their solvated interfaces with the POPC and DMPC bilipid layers, where 0% (red), 10% (orange), 25% (yellow), 50% (green), 75% (cyan) and 95% (dark blue) from the initial water molecules were removed, as a function of the temperature of the cooling quench process.

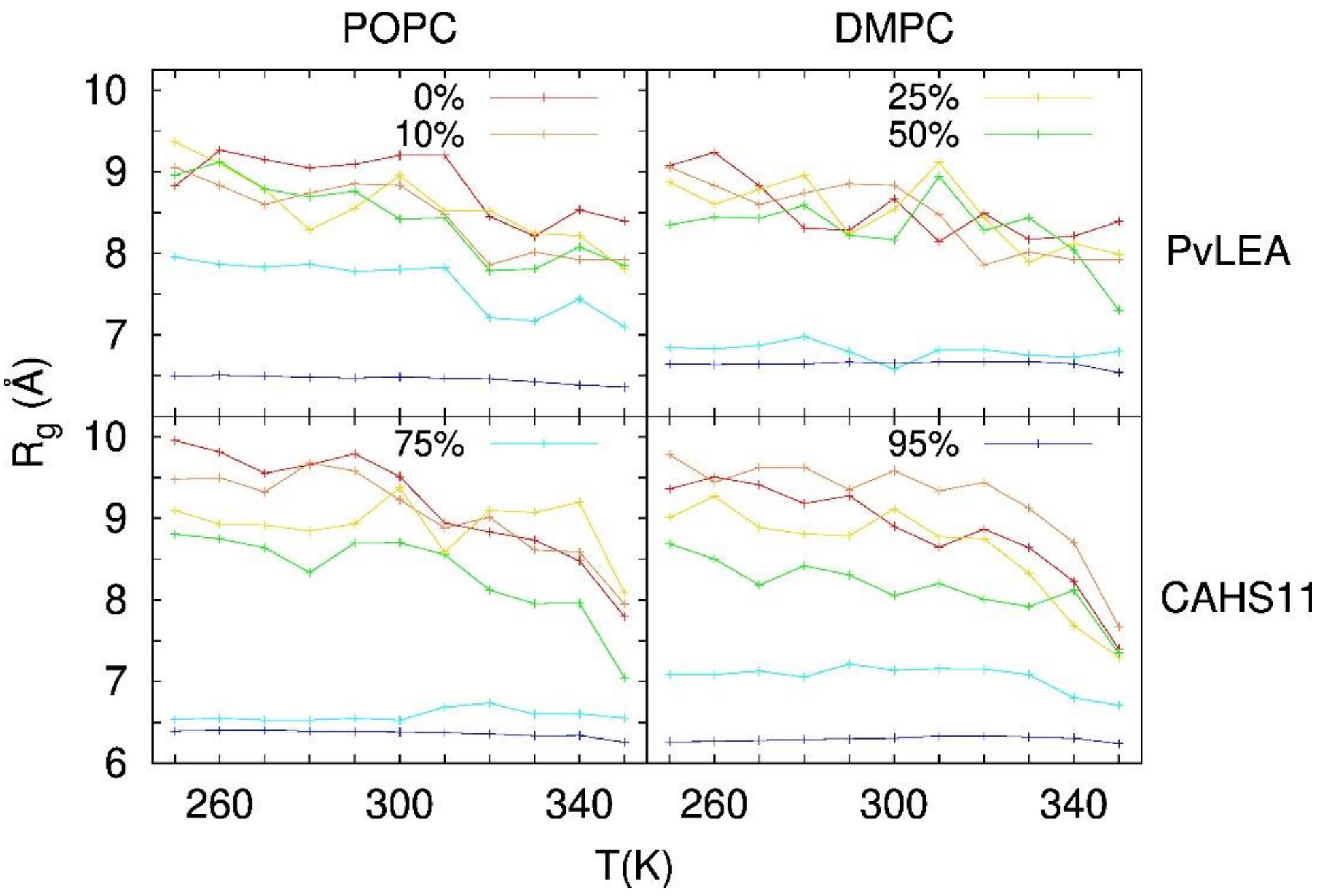

Figure S32. Radius of gyration (R_g) in Å for the PvLEA and CAHS11 proteins at their solvated interfaces with the POPC and DMPC bilipid layers, where 0% (red), 10% (orange), 25% (yellow), 50% (green), 75% (cyan) and 95% (dark blue) from the initial water molecules were removed, as a function of the temperature of the cooling quench process.

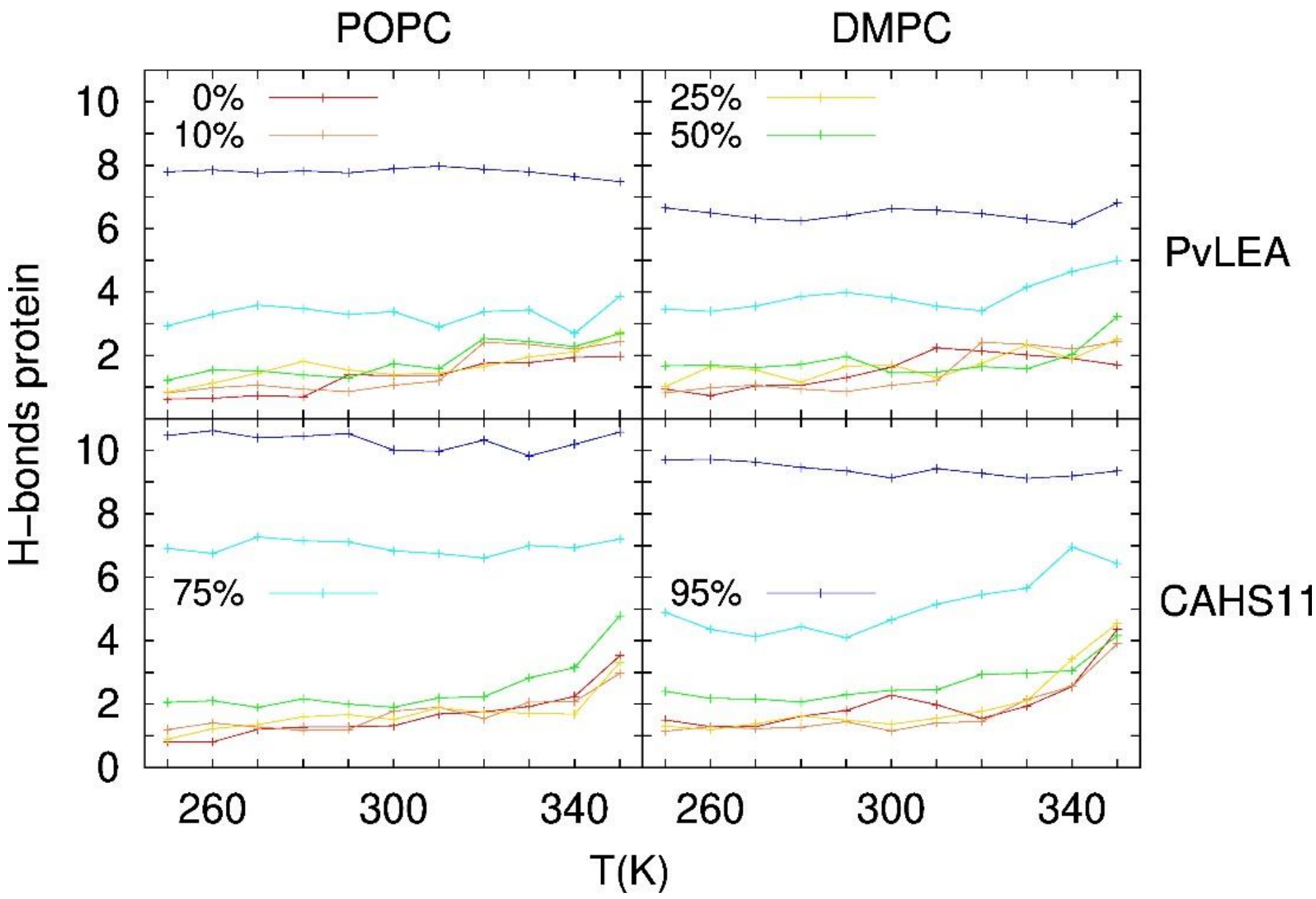

Figure S33. Number of H-bonds formed within the PvLEA and CAHS11 protein structures at their solvated interfaces with the POPC and DMPC bilipid layers, where 0% (red), 10% (orange), 25% (yellow), 50% (green), 75% (cyan) and 95% (dark blue) from the initial water molecules were removed, as a function of the temperature of the cooling quench process.

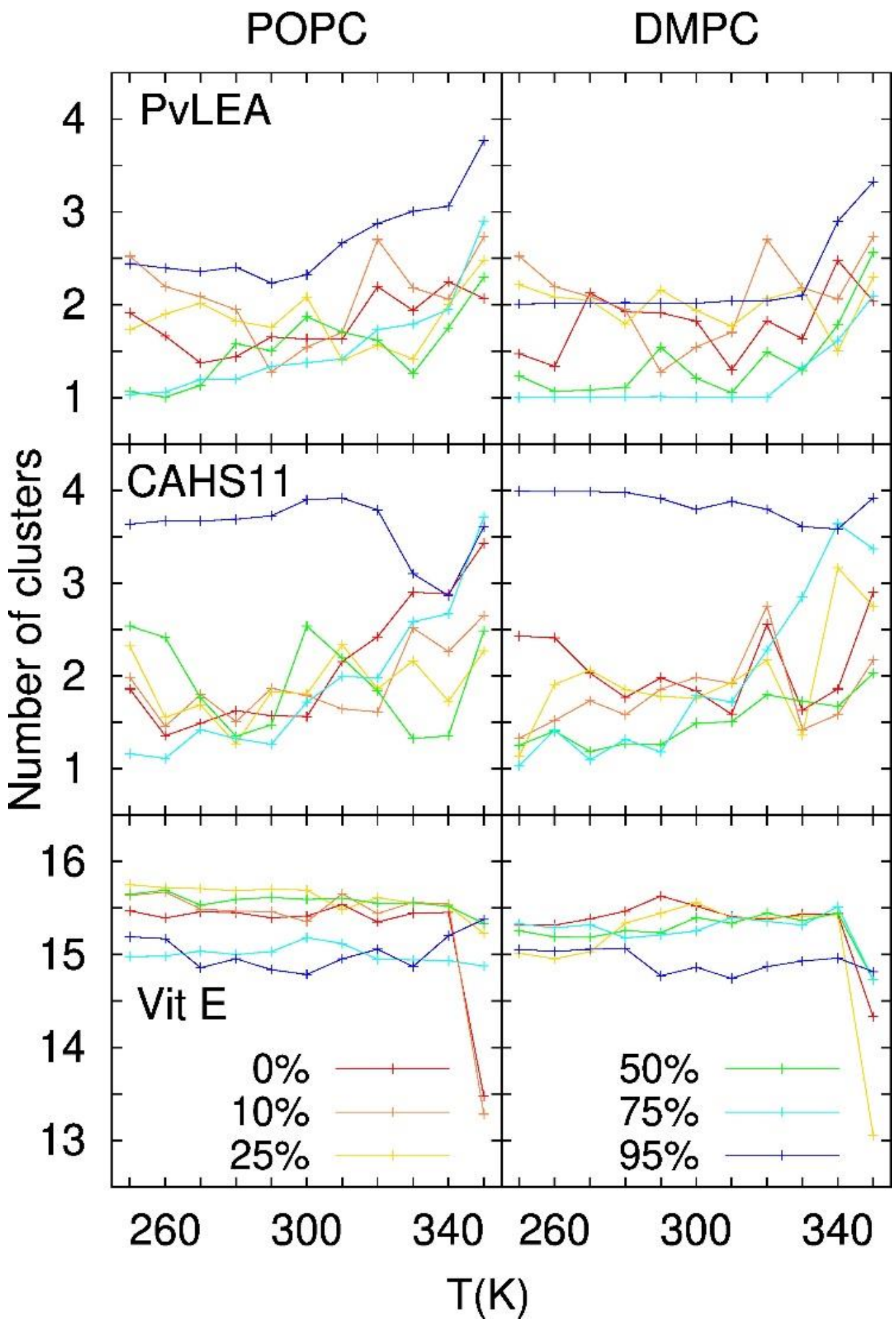

Figure S34. Number of clusters formed by PvLEA, CAHS11 and vitamin E molecular systems at their solvated interfaces with the POPC and DMPC bilipid layers, where 0% (red), 10% (orange), 25% (yellow), 50% (green), 75% (cyan) and 95% (dark blue) from the initial water molecules were removed, as a function of the temperature of the cooling quench process.

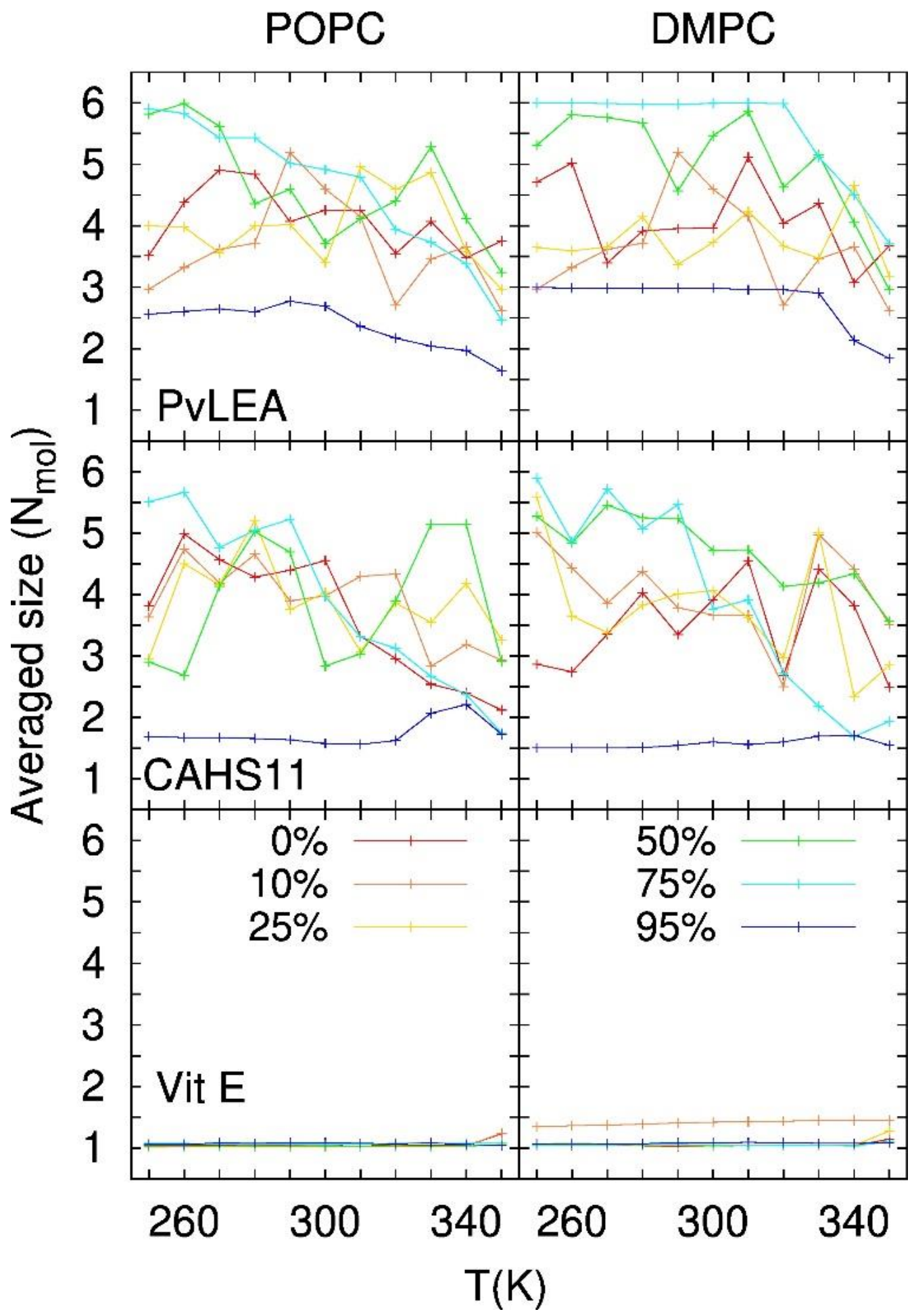

Figure S35. Averaged cluster size in number of molecules/cluster formed by PvLEA, CAHS11 and vitamin E molecular systems at their solvated interfaces with the POPC and DMPC bilipid layers, where 0% (red), 10% (orange), 25% (yellow), 50% (green), 75% (cyan) and 95% (dark blue) from the initial water molecules were removed, as a function of the temperature of the cooling quench process.

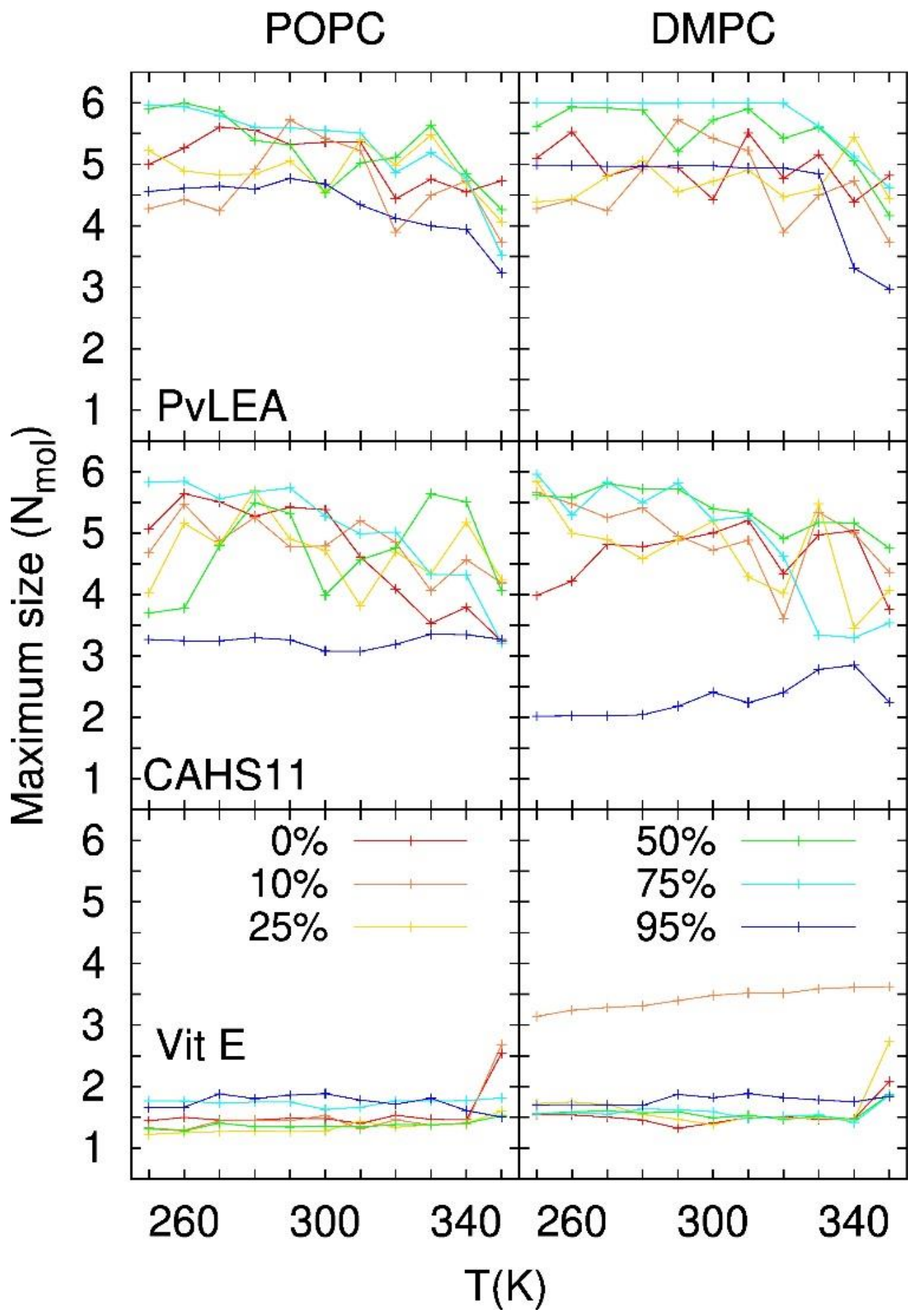

Figure S36. Maximum cluster size in number of molecules/cluster formed by PvLEA, CAHS11 and vitamin E molecular systems at their solvated interfaces with the POPC and DMPC bilipid layers, where 0% (red), 10% (orange), 25% (yellow), 50% (green), 75% (cyan) and 95% (dark blue) from the initial water molecules were removed, as a function of the temperature of the cooling quench process.
